# BubbleTree: an intuitive visualization to elucidate tumoral aneuploidy and clonality using next generation sequencing data

**DOI:** 10.1093/nar/gkv1102

**Published:** 2015-11-17

**Authors:** Wei Zhu, Michael Kuziora, Todd Creasy, Zhongwu Lai, Christopher Morehouse, Xiang Guo, Yinong Sebastian, Dong Shen, Jiaqi Huang, Jonathan R. Dry, Feng Xue, Liyan Jiang, Yihong Yao, Brandon W. Higgs

**Affiliations:** 1 Translational Bioinformatics, MedImmune, Gaithersburg, MD 20878, USA; 2 Oncology iMed, AstraZeneca, Waltham, MA 02451, USA; 3 Clinical Biomarkers and Computational Biology, MedImmune, Gaithersburg, MD 20878, USA; 4 Department of Liver Surgery and Liver Transplantation, Renji Hospital, Shanghai Jiaotong University School of Medicine, Shanghai, China; 5 Department of Pulmonary, Shanghai Chest Hospital, Shanghai Jiao Tong University, Shanghai, China

## Abstract

Tumors are characterized by properties of genetic instability, heterogeneity, and significant oligoclonality. Elucidating this intratumoral heterogeneity is challenging but important. In this study, we propose a framework, BubbleTree, to characterize the tumor clonality using next generation sequencing (NGS) data. BubbleTree simultaneously elucidates the complexity of a tumor biopsy, estimating cancerous cell purity, tumor ploidy, allele-specific copy number, and clonality and represents this in an intuitive graph. We further developed a three-step heuristic method to automate the interpretation of the BubbleTree graph, using a divide-and-conquer strategy. In this study, we demonstrated the performance of BubbleTree with comparisons to similar commonly used tools such as THetA2, ABSOLUTE, AbsCN-seq and ASCAT, using both simulated and patient-derived data. BubbleTree outperformed these tools, particularly in identifying tumor subclonal populations and polyploidy. We further demonstrated BubbleTree's utility in tracking clonality changes from patients’ primary to metastatic tumor and dating somatic single nucleotide and copy number variants along the tumor clonal evolution. Overall, the BubbleTree graph and corresponding model is a powerful approach to provide a comprehensive spectrum of the heterogeneous tumor karyotype in human tumors. BubbleTree is R-based and freely available to the research community (https://www.bioconductor.org/packages/release/bioc/html/BubbleTree.html).

## INTRODUCTION

A common characteristic shared among malignant cancerous cells is impaired DNA repair, which in turn leads to genome instability (1–5). As a result of this instability, tumor cells progressively acquire additional DNA aberrations throughout the lifetime of a tumor. Analogous to Darwinian natural selection, cancer progression can be regarded as a process of clonal expansion (6,7). Specifically, each tumor cell is a single species or ‘clone’ characterized by a specific genetic makeup, and the tumor evolution is driven by competition for growth between clones within the adaptive tissue microenvironment (8–11). The resulting tumor therefore comprises a heterogeneous mixture of genetically distinct cell populations. This heterogeneity enables a tumor to adapt to differing selective pressures such as drug therapy to prolong tumor survival (12), hence it is of great importance to understanding the inherent tumor clonal structure.

Clonal heterogeneity can ideally be evaluated using single-cell technology (13–15), or alternative low-resolution but more cost-effective multi-cell approaches such as cell sorting with flow cytometry coupled to next generation sequencing (NGS) (16). So far, however, most genomic and genetic alterations are still measured from multi-cell specimens within mixed cell populations, due to limitations of precision in biopsy extraction, cellular localization, assay throughput and cost.

Accordingly, many bioinformatics tools have been developed to deconvolute the prevalence of tumor cells and clonality within multi-cell tumor masses (17). Of these tools, several are regularly cited in cancer literature - ASCAT (18), ABSOLUTE (19), AbsCN-seq (20) and THetA2 (21). ASCAT is a pioneer of the tools in this category and ABSOLUTE has been widely adopted in NGS data analyses. AbsCN-seq offers comparable performance to ABSOLUTE and ASCAT using a simpler less complicated algorithm (20). All three algorithms, however, focus on the problem of tumor purity (i.e. quantifying tumor cell content versus non-tumor cell ‘contamination’ in a biopsy). In doing so they assume that tumor cells are monogenomic (i.e., without subclonal tumor populations), and thereby underperform in cases where subclones constitute a significant proportion of the total tumor mass. In reality, intratumoral heterogeneity is common in most cancers, and is thus a necessary component for tumor cell prevalence estimates (9,22). In contrast to these three tools, THetA2 employs a sophisticated algorithm to determine tumor subpopulations using copy number ratio with an optional component to select the optimal solution using B-allele frequency (BAF) (19).

Though each tool has its own strength for characterizing the complex intratumoral heterogeneity, we aim to improve upon such methods for tumor clonal structure and ultimately better inform cancer diagnosis, prognosis and treatment decisions. In this study, we propose a novel visualization-based framework termed BubbleTree. Using somatic copy number alterations (SCNAs) and BAFs which are routinely obtained from NGS data, BubbleTree provides an intuitive graph for simple estimates of tumor purity, ploidy, and clonality. Additionally, a heuristic approach to mimic and automate the manual interpretation of the BubbleTree graph is provided, which can speed automated analyses for large patient studies. We demonstrate that BubbleTree can outperform its counterpart tools in our testing of both simulated and patient-derived datasets, suggesting this framework as an attractive approach for application in the characterization of tumor heterogeneity.

## MATERIALS AND METHODS

### BubbleTree model and diagram

The basic BubbleTree model is similar to that Bao *et al*. has described for AbsCN-seq (20): the heterogeneous tumor genome can generally be considered a chain of disjointed *homogeneous* segments. We use a three-tuple (*x*, *y*, *p*) to denote one homogeneous genomic segment in a diploid species, where *x* and *y* denote allele-specific copy number at a bi-allelic genomic locus (*x* ≤ *y*, without loss of generality), and *p* is the prevalence of the specific segment in the tumor sample. Then, we have:

\begin{eqnarray*} {\rm Expected}\,{\rm copy}\,{\rm number}\,{\rm overline}\, \\ {{\rm CN}} = 2 \times (1 - p) + (x + y) \times p \end{eqnarray*}

(1) \begin{equation*} {\rm Copy}\,{\rm number}\,{\rm ratio},R {=} \overline {{\rm CN}} /2 {=} (1{-}p) + \frac{{x + y}}{2}{\times}p \end{equation*}

\begin{equation*} B\,{\rm allele}\,{\rm frequency},{\rm BAF} = \frac{{y \times p + 1 \times (1 - p)}}{{(x + y) \times p + 2 \times (1 - p)}} \end{equation*}

and the heterozygous-deviation score (HDS),

(2) \begin{equation*} |{\rm BAF} - 0.5| = \frac{{p \times |y - x|}}{{2 \times [(x + y) \times p + 2 \times (1 - p)]}} \end{equation*}

**Figure 1.**
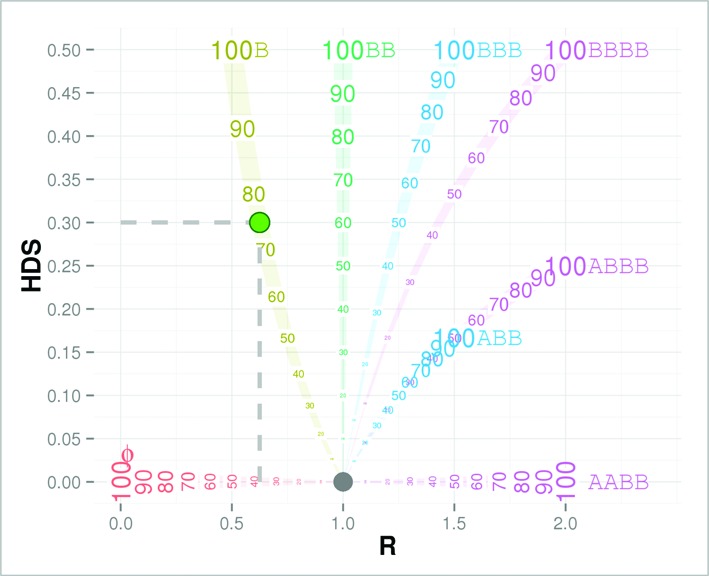
BubbleTree graph. In this ***R***-HDS plot, ***R*** score indicates the copy number ratio between the tumor and matched normal sample, and HDS, heterozygous-deviation score, is defined by |BAF-0.5|. The tree branches represent the integer ASCNs (allele-specific copy numbers) as calculated by equations (1) and (2), with the tick value marking the likely prevalence in position. The branches are labeled by the genotypes of the SCNAs, for instance, the empty set symbol Ø represents, for the homozygous deletion, B for one copy loss, AB for the normal disomy, BB for the cnLOH (colored in green), ABB and BBB for the copy gain (colored in blue), and so on. For example, the green filled circle represents the 3-tuple value (*x* = 0, *y* = 1, *P* = 0.75), that is, one copy loss (the branch B) with 75% prevalence. By equations 1 and 2, it has 0.625 and 0.3 for the ***R*** score and HDS, respectively (highlighted by the dotted lines).

Based on equations (1) and (2), we are able to calculate an expected ***R*** score (copy number ratio) and HDS for a segment (*x*, *y*, *p*) in the ***R***-HDS plot. For example, the segment with the 3-tuple value (0, 1, 0.75) (i.e. one copy loss with 75% prevalence) will have 0.625 and 0.3 for the ***R*** score and HDS, respectively. Accordingly, we construct the branches of the BubbleTree (Figure 1), that is, the prediction of our model for the integer allele-specific copy numbers (ASCNs). Here, we also provide the designations of A or B to indicate ASCNs of the SCNAs in a similar style as the allele genotype, where, without loss of generality, the copy number of A is always no more than that of B. For instance, a normal disomy state (*x* = 1, *y* = 1) is represented by AB. Other pre-calculated modes for the various SCNAs include a homozygous deletion (Ø; *x* = 0, *y* = 0), one-copy loss (B; *x* = 0, *y* = 1), copy-number neutral LOH (cnLOH; BB; *x* = 0, *y* = 2), one-copy gain (ABB; *x* = 1, *y* = 2 or BBB; *x* = 0, *y* = 3), etc., with different likely prevalence *p* (i.e. 10%, 20%, …, 100%), to construct the branches of the BubbleTree (Figure 1 and Supplementary Figure S1).

### Three-step approach to interpret the BubbleTree graph

The bubbles (i.e. the leaves) are represented on the ***R***-HDS plot with positions derived from NGS data and sizes proportional to the segment lengths. In most cases, the ASCN and prevalence (i.e. *x*, *y* and *p*) of the tumor clone or subclone, marked by one particular SCNV, can be inferred by its proximity to the particular branch.

The interpretation of the BubbleTree graph can generally be described by three steps: (i) Determine the tumor ploidy and adjust the copy ratio ***R*** accordingly. Notably, the measured copy ratio ***R’*** from a tumor is not the actually copy ratio score ***R***, and the former needs to be adjusted by the relative tumor sample ploidy τ_t_, such as:

\begin{equation*} \Phi = (\tau _{\rm t} \,p + 2(1 - p))/2\,{\rm and}\,R = R^\prime*\Phi \end{equation*}

**Figure 2.**
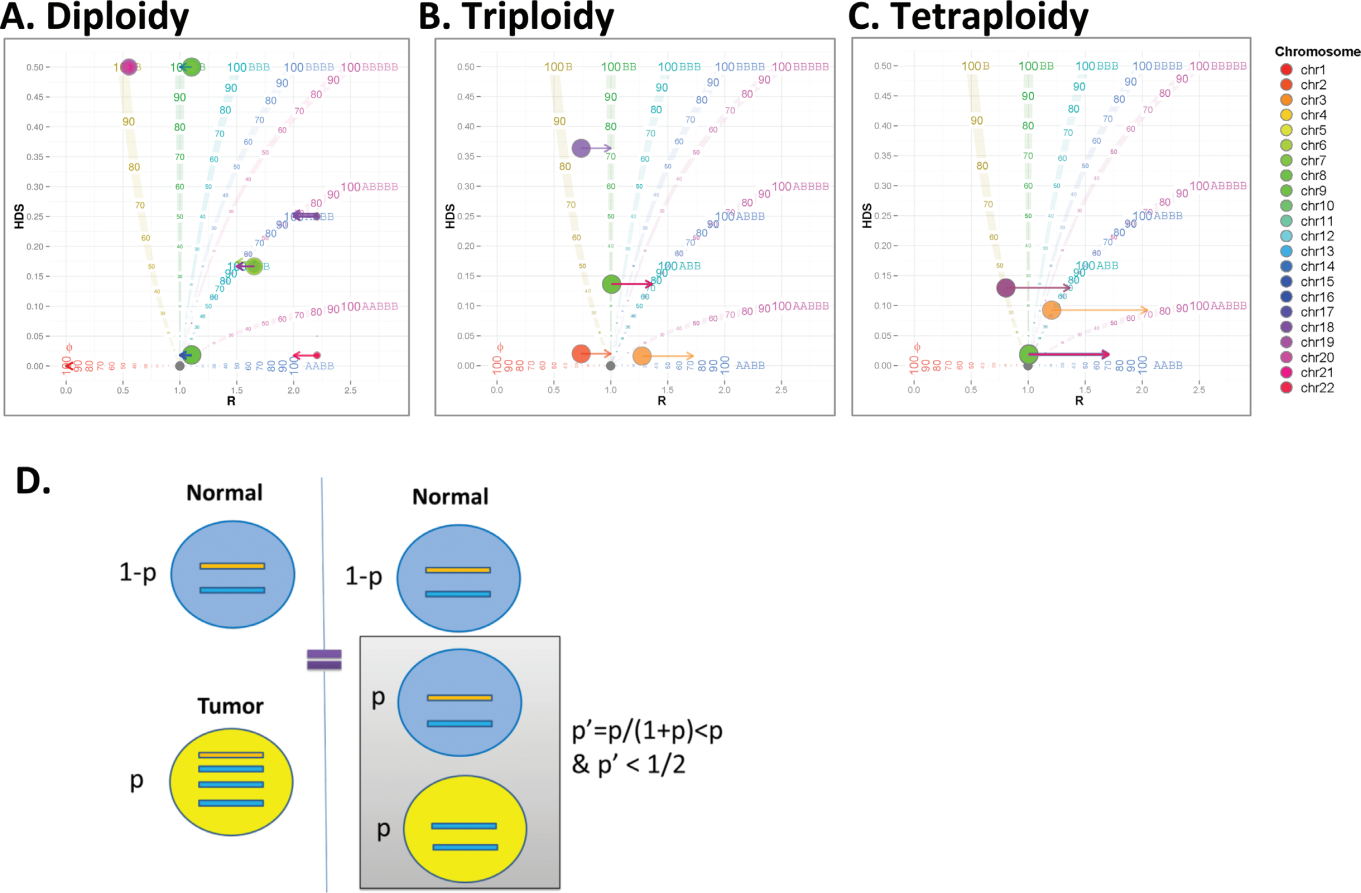
Copy ratio score adjustment and the lack of identification due to high tumor ploidy. The copy ratio score measured from the NGS data is not the actual copy ratio score, the adjustment *Φ* depends on the tumor purity and the tumor ploidy *Φ =* (*τ_t_p* + 2(1 – *p*))/2. In the plots, (**A**) the diploidy, (**B**) triploidy and (**C**) tetraploidy states are illustrated by the simulated data sets: sam2 (*P* = 100%), sam14 (*P* = 72.3%) and sam15 (*P* = 66.7%), respectively. The bubbles represents the genomic segments positioned by the HDS scores and the raw ***R*** scores, with the color corresponding to each chromosome. The horizontal arrows indicate the shift of the bubbles following copy ratio adjustment. (**D**) One tetraploiy sample with purity *p* could yield the same HDS and ***R*** scores as a diploidy sample with the purity *p’* = *p*/(1 + *p*) < *p*, where *p’* ≤ 0.5. Specifically, it is difficult to distinguish the diploidy tumor with low purity from the tetraploidy tumor with a higher purity by the HDS and ***R*** scores alone.

We provide examples of this ploidy adjustment in the BubbleTree graph into three of the most common ploidy states: *τ_t_* ≈ 2, 3, and 4 in Figure 2. Figure 2A shows the most common scenario where the tumor ploidy state is *τ_t_* ≈ 2. If *τ_t_* < 2, the observed copy ratio ***R’*** is overestimated and all bubbles (segments) need to be horizontally shifted to the left accordingly; otherwise, vice versa. In Figure 2B, the tumor genome is approximately triploidy (*τ_t_* ≈ 3); this state can easily be distinguished from diploidy (or other even number ploidy states) by the extensive LOH: the bubbles are not centered around (1, 0) in the ***R***-HDS plot but is about (1, *h*) instead, where 0 << *h* ≤ 1/6. Again, the HDS score is not affected by the tumor ploidy adjustment, so the centered segments should be right-shifted to the ABB branch in the BubbleTree graph. In Figure 2C, there are no observed dramatic copy number variations in the simulated sample *sam15*, thus there is no difference in goodness-of-fit between the high purity tetraploidy prediction (*P* = 70%) and the low purity diploidy prediction (*p’* = *p*/(1 + *p*) = 41%) in terms of ***R*** and HDS scores alone (Figure 2C and D). In this case, extra tumor specimen information such as ploidy and/or purity is required to make the correct prediction. The monoploidy (*τ*_t_ = 1) and the other high ploidy states (e.g. *τ_t_* = 5, 6 or beyond) are less common and difficult to be determined without additional information, so they were not explored in this study.

**Figure 3.**
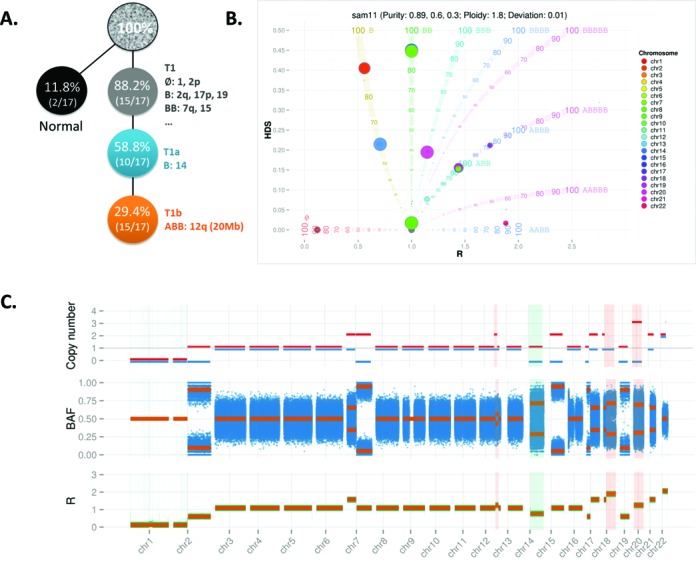
Automated BubbleTree prediction for simulated sample sam11. (**A**) Tumor sample sam11 was a mixture of the simulated normal cells, and tumor (sub)clones T1, T1a and T1b with a ratio of 2:5:5:5 (Table 2 and Supplementary Figure S2). As T1a and T1b are the subclones derived from the tumor clone T1, they share some SCNAs with T1, like the homozygous deletion (i.e. represented by the empty set symbol Ø here) in chr1 and 2*p*, one copy loss (i.e. the genotype B) of chr2q, 17p and 19 and the copy-number neutral LOH (i.e. the genotype BB) of chr7q and 15. T1a and T1b have the common marker in B:14, that is one copy loss in chr14, while T1b has one unique 20Mb copy gain in chr12. (**B**) BubbleTree graph after copy ratio score adjustment, where the genomic segments are located in the expected positions. (**C**) The details of the prediction are display in three tracks: allele-specific copy number (blue bar for the score *x* and red bar for *y*), the expected BAFs on top of the observed BAFs of the germline heterozygous loci, and the expected ***R*** scores on top of the adjusted copy ratio scores. The consistency between the prediction and the measured scores indicates the accuracy of the BubbleTree method for sam11. Unique SCNA markers for the subclones are highlighted in light blue (for the subclone with prevalence of 60%) and light orange (for the subclone with prevalence of 30%). Detailed text output is available in Supplementary Data File 2.

After ploidy adjustment (i.e. horizontal shift of bubbles to the branches in the BubbleTree graph), the next step is to (ii) Identify the tumor purity and subclonal populations. To reduce likely variation due to the small genomic segments (e.g. centromeres and low complexity regions), we focus on the large segments in this step. The segments with the highest prevalence indicate the dominant tumor clone, and thus this prevalence (*p*) represents the purity of the tumor. Those SCNV segments with distinctly lower prevalence indicate the existence of the tumor cell subpopulations (Figure 3).

The last step is to (iii) predict (*x, y, p*) scores for each segment. The prevalence of the tumor (sub)clone is detected in the second step, preferably using the large segments proximities to the unambiguous branches (e.g. ABB and ABBB; see Figure 1 and Supplementary Figure S1). It is probable that the number of tumor subclones in a sample is limited and most should be captured by the large segments in the second step. In this third step, we may deduce the most likely *x* and *y* scores for the remaining segments (including those on the ambiguous branches like, ABB, ABBB, etc.), using the grid search again but with the known prevalence scores.

To complement the manual estimates of the BubbleTree graph and speed automated interpretation, a heuristic model was developed in a similar stepwise fashion, as described in the Supplementary Methods.

### Samples and NGS sequencing

#### Matched primary and recurrent tumors in two patients with hepatocellular carcinoma (HCC)

Three fresh frozen specimens were procured from each of the two HCC patients who experienced tumor recurrence following orthotopic liver transplantation (OLT): peripheral blood, primary tumor and recurrent tumor. The peripheral blood sample served as the normal control of the other remaining samples for each patient (Table 1). Informed written consent was obtained from each patient and the study protocol conformed to the ethical guidelines of the 1975 Declaration of Helsinki as reflected in a priori approval by the Ethics Committee of Renji Hospital. Tumor purity was evaluated by pathology assessments for each tumor specimen.

**Table 1.** The patient and sample information

Patient ID	Gender	Age at diagnosis	Sample ID	Sample type	Tissue	Sample collection date	Sample description^a^	NGS^b^
HCC4	M	59	HCC4.Blood	Normal	Blood	NA		WES
			HCC4.Primary.Tumor	Hepatocellular carcinoma	Liver	Feb, 2012	Prior to OLT	WES
			HCC4.Recurrent.Tumor	Hepatocellular carcinoma	Liver	Sep, 2012	recurrent HCC post to OLT	WES
HCC11	F	57	HCC11.Blood	Normal	Blood	NA		WES
			HCC11.Primary.Tumor	Hepatocellular carcinoma	Liver	Dec, 2011	Piror to OLT	WES
			HCC11.Recurrent.Tumor	Hepatocellular carcinoma	Lung	April, 2013	Lung metastasis post of OLT	WES
DM	M	51	lung_normal	Adjacent normal	Lung	Mar, 2012		WES/WGS
			lung	Adenocarcinoma	Lung	Mar, 2012	Primary lung cancer	WES/WGS
			ovary_normal	Adjacent normal	Ovary	Aug, 2011		WES/WGS
			ovary	Ovarian serous papillary cystadenocarcinoma	Ovary	Aug, 2011	Primary ovarian cancer	WES/WGS

^a^OLT = orthotopic liver transplantation.

^b^NGS = next genearation sequencing; WES = whole exome sequencing; WGS = whole genome sequencing.

#### Matched primary lung and ovarian tumor specimens from a patient with synchronous double malignancy

The 51-year-old Chinese female never-smoker patient presented with synchronous double malignancy in both lung and ovary and was treated with first line chemotherapy and epidermal growth factor receptor – tyrosine kinase inhibitor (EGFR-TKI) targeted therapy. Adenocarcinoma lung and ovarian serous carcinoma tumor biopsies (fresh frozen) and the matched adjacent normal tissues were procured from this patient (Table 1). Tumor purity and pathology classification was evaluated and confirmed by pathologist assessments for each sample. The study protocol was approved by the Ethics Committee of Shanghai Chest Hospital. Informed consent in writing was obtained from the patients and the study protocol conformed to the ethical guidelines of the 1975 Declaration of Helsinki.

#### Simulated WGS data

A simulated set of tumors were produced by the following methodology: (i) Introduction of germline variants into to the human reference genome hg19 to generate a normal diploid genome (N), (ii) creation of tumor clones (T1 and T2), subclones (T1a and T1b), and polyploidy (T3, T4) by introducing somatic copy number variations of various sizes and genotypes (Supplementary Figure S2). Then, the program art_illumina (23) was used to generate simulated NGS read sets for each of the seven simulated normal/tumor genomes at a variety of depths. Finally, we constructed combinations the NGS read sets to create 15 simulated whole genome data sets of ∼30× depth (Table 2). For instance, the sample sam11 is an admixture of N (depth of 2), T1 (depth of 5), T1a (depth of 5) and T1b (depth of 5), with a collective total depth of 34 (2 × 2 + 5 × 2 + 5 × 2 + 5 × 2 = 34; as N, T1, T1a and T1b are all diploidy) by using haploid human genome hg19 as a reference sequence. Accordingly, the tumor purity of sam11 is expected to be 30/34 = 88.2% (Table 2).

**Table 2.** The simulated data sets and the benchmark results

Sample	N^a^ (normal)	T1^a^	T1a^a^	T1b^a^	T2^a^	T3^a^ (Triploidy)	T4^a^ (Tetraploidy)	Tumor cell purity (%)	BubbleTree^b^	THetA^b^	ABSOLUTE^b,c^	AbSeqCN^b^	ASCAT^b^
sam1	15x2							0					
sam2		15x2						100	1	1.00	1 (16th)	0.67	1
sam3	3x2	12x2						80	0.815, 0.2	0.80	0.8 (8th)	0.53	0.9
sam4	6x2	9x2						60	0.6	0.60	0.6 (8th)	0.59	0.45
sam5	9x2	6x2						40	0.406	0.60	0.4 (9th)	0.39	0.26
sam6	12x2	3x2						20	0.247	0.67	0.2 (8th)	0.25	NA
sam7	2x2	8x2	8x2					88.9, 44.4	0.896, 0.45	0.89	0.89 (3rd)	0.56	0.97
sam8	2x2	8x2	4x2	4x2				88.9, 44.4, 22.2	0.896, 0.444	0.89	0.89 (2nd)	0.54	0.96
sam9	2x2	8x2			8x2			44.4	0.453	0.51	0.67 (12th)	0.57	0.41
sam10	2x2	11x2			5x2			61.1, 27.8	0.615	0.66	0.61 (10th)	0.58	0.6
sam11	2x2	5x2	5x2	5x2				88.2, 58.8, 29.4	0.893, 0.6, 0.3	0.88	0.88 (5th)	0.54	0.89
sam12	2x2	8x2	4x2		4x2			66.7, 44.4, 22.2	0.672, 0.31	0.70	0.66 (11th)	0.58	0.5
sam13	2x2	8x2	5x2	3x2				88.9, 44.4, 27.7, 16.7	0.895, 0.45	0.89	0.89 (6th)	0.54	0.96
sam14	3x2					8x3		72.3	0.73	0.53	0.31 (10th)	0.29	0.62
sam15	3x2						6x4	66.7	0.696	1.00	0.67 (16th)	0.22	0.26

^a^(Coverage) x (Ploidy) = (Total coverage). The generation of the normal sample (*N*) and the tumor clones (i.e., T1, T1a, T1b, T2, T3 and T4) is described in Supplementary Figure S2.

^b^Tumor purities estimated by the programs. The prevalences of the tumoral subclones are also listed for BubbleTree (as seperated by commas).

^3^The number in parenthesis represents the rank of the selected prediction in the ABSOLUTE output.

#### TCGA dataset

The 20 matched TCGA WES bam files, which had been used in the THetA2 publication (21), were downloaded Cancer Genomics Hub (https://cghub.ucsc.edu/). The sample TCGA-06-0185-01A-01W-0254-08 was removed, due to its low read count (Supplementary Data File 1). The remaining 19 matched samples were processed to the subsequent germline variant calling and somatic copy number variation analyses.

### DNA sequence read mapping and variant calling

All human patient samples were sequenced by whole exome sequencing (WES) with a read depth of ∼100× and the samples from the double malignancy (DM) patients were additionally sequenced by WGS with a read depth of ∼30× (Supplementary Table S1). Paired-end WGS and WES reads (2 × 90) were generated by Beijing Genomics Institute (BGI) using the Illumina standard library preparation and sequencing protocols as described in (24). Detailed explanation of the subsequent somatic and germline variant calling is provided in the Supplementary Methods.

### Somatic copy number variation (SCNV) analysis

R packages *DNAcopy* (25) and *ExomeCNV* (26) were used to identify CNVs based on the read depth derived from the WGS and WES alignments using default parameters. Copy number ratios (***R*** scores) of CNVs were calculated across the whole exome or genome as the difference between the tumor and paired adjacent normal comparisons.

### Defining the *R* and HDS scores for each segment

The segments called from the previous SCNAs have similar ***R*** scores for the loci within each segment, but the HDS for loci may not necessarily be consistent within each segment. We calculated the median and standard deviation values of the tumor BAFs of the heterozygous germline loci for each segment and discarded those segments with high HDS variation (empirically, standard deviation > 0.2). The consequent genomic segments are *homogeneous* in terms of both HDS and ***R*** scores. The median values of ***R*** scores and HDS of each *homogeneous* segment define the X–Y coordinates of the segment (i.e. the bubble leave) in the BubbleTree diagram over the branches (i.e. the predictions of the integer ASCNs). The details of the procedure are available from the source code of the BubbleTree package distributed through Bioconductor (https://www.bioconductor.org/packages/release/bioc/html/BubbleTree.html). A refined solution to identify the homogeneous segment is proposed in the Discussion section.

## RESULTS

### Estimates of tumor purity with BubbleTree on simulated WGS data

To evaluate the performance of the BubbleTree model, we simulated 15 tumor samples under various frequencies and sizes of CNVs, including some with stepwise tumor clonal hierarchy, independent tumor origin, and polyploidization, most of which are admixtures with normal cells. The simulation process is described in Supplementary Figure S2 and Table 2 and a detailed example of simulation and the BubbleTree prediction is demonstrated in Figure 3. We further evaluated other programs such as ASCAT (27), AbsCN-seq (20), ABSOLUTE (19) and THetA2 (21) to predict tumor purity using the simulated data (Table 2).

**Figure 4.**
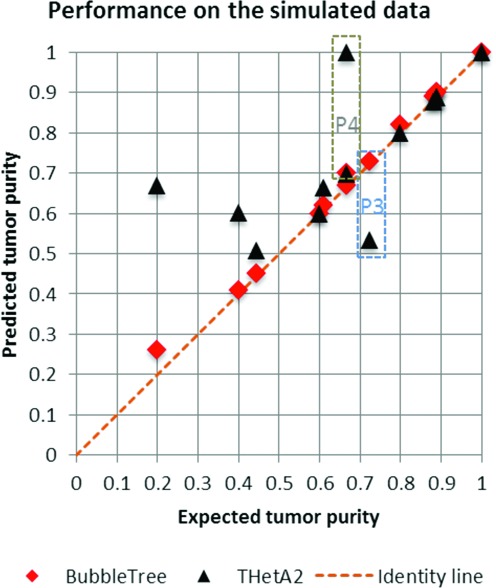
Performance of BubbleTree on a simulated WGS data set. The BubbleTree predictions are adjacent to the dotted identity line, suggesting the predicted tumor purities are close to the expected tumor purities (Table 2). The predictions from THetA2 are depicted by black triangles. It is noticeable that THetA2 failed to provide the proper predictions for the low purity (sam6 at the 20% purity), and high tumor ploidy outlined by the dotted rectangles P3 (triploidy sample sam14) and P4 (tetraploidy sample sam15).

Overall, BubbleTree exhibited the most accurate performance, followed by THetA2 and ABSOLUTE, when using simulated WGS data (Table 2). Low tumor purity, together with high ploidy state, is one of the challenging tumoral conditions for estimation, as shown in Figure 2D, where THetA2 failed to provide predictions (Figure 4). ABSOLUTE provided multiple estimates for each prediction, which did contain the right solution most of the time. However, out of the 14 simulated datasets, ABSOLUTE only ranked the correct solution twice within one of the top three of the multiple potential solutions provided (Table 2), imposing a challenge in practical application to large cohorts of patient tumor specimens, where each prediction cannot be efficiently interrogated amongst a panel of potential solutions.

### Evaluation of tumor heterogeneity in simulated WGS and The Cancer Genome Atlas (TCGA) WES data

Purity estimation indicates the prevalence of overall cancerous cells within a given patient tumor. Given the stepwise tumor clonal hierarchy, the tumor purity is simply the prevalence of the dominant tumor clonal population. However, understanding the entire tumor clonality is more challenging. The simulated WGS tumor samples sim7-sim13 are mixtures of two or more tumoral clones, each of which include specific SCNAs (Figure 3, Supplementary Figure S2 and Table 2). BubbleTree successfully predicted the tumor subclones in each of these simulated tumor samples (Table 2 and Figure 3). Detailed text and graph output are available in Supplementary Data Files 2–4.

**Figure 5.**
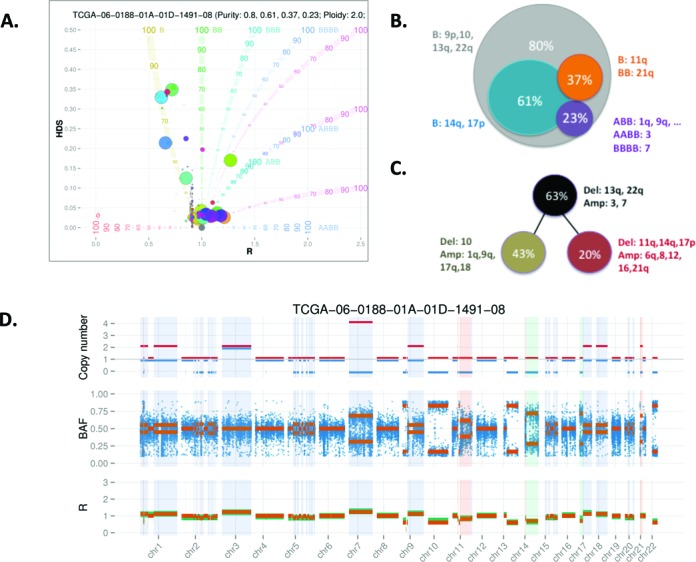
BubbleTree prediction for the TCGA WES data TCGA-06-0188. (**A**) BubbleTree prediction for the TCGA sample TCGA-06-0188 and, (**B**) the interpretation of the tumor clonality. We may infer that this sample harbors a dominant tumor clone with purity of 80%, marked by the DNA copy number loss on several chromosomes including chr10. There are three subclones with prevalence 61%, 37% and 23%, respectively; (**C**) prediction from the THetA2 publication, where tumor purity was predicted as 63% and the chr10 deletion, different from the BubbleTree prediction, was a marker of the subclone (*P* = 43%); (**D**) BubbleTree predictions are displayed in tracks in the same way as Figure 3C. In particular, the unique SCNAs for the smallest subclone (*P* = 23%) are highlighted by the light purple rectangles along the tracks. Due to low prevalence, the consequently predicted ***R*** and BAF scores are almost identical to the normal disomy (i.e. no SCNAs), with exception to the SCNA in chr7 with the predicted genotype BBBB (i.e. one copy of the parental chromosomes was lost and the other one was amplified to four copies). Noticeably, THetA2 reconstructs the tumor genome, so that chr10 deletion and chr14q deletion belong to two distinct tumor subclones. BubbleTreeassumes that all subclones share the markers carried by the dominant clone (*P* = 80% in this particular example), that is, one subclone (*P* = 61%) should have both chr10 and chr14q deletions and the remaining subclone (*P* = 80–61 = 19%) should have a deletion in chr10 but not in chr14q. We did not attempt to infer the interactions among the subclones.

THetA2 is one of the few programs that assess the prevalence of the tumor subclonal populations. To provide an unbiased comparison between THetA2 and BubbleTree, we used the same The Cancer Genome Atlas (TCGA) data used in the THetA2 publication (21). Specifically, we focused on the five TCGA WES tumor/normal matched samples where detailed subclonal predictions were fully described in the study (21): TCGA-06-0214, TCGA-56-1622, TCGA-06-0188, TCGA-06-0145 and TCGA-AO-A0JF. Results (Supplementary Data Files 2–4) indicated that BubbleTree provided similar predictions for the three TCGA samples TCGA-06-0214, TCGA-AO-AOJF and TCGA-06-0145, but showed significant difference in the predictions for TCGA-56-1622 and TCGA-06-0188. For TCGA-06-0188, BubbleTree predicted purity of 80%, with the dominant tumor clone marked by clonal deletions at chromosomes 9p, 10, 13q and 22q (Figure 5). In contrast, THetA2 predicted the copy losses at chromosomes 9p, 13q and 22q as clonal deletions (*P* = 63%) and the chromosome 10 loss as a subclonal deletion (*P* = 43%), contradicting the similar HDS score seen between chromosome 10 and chromosomes 9p, 13q and 22q (Figure 5C; and Figure 5 in (21)).

In the case of TCGA-56-1622, BubbleTree predicted the tumor genome to be triploidy (tumor ploidy = 3.1) and purity estimation of 88% is close to the 90% purity assessed by the pathologist. Our prediction successfully addressed the reason that no LOH resulted from the obvious ‘copy number loss’ in chromosome 1. That is, chromosome 1 in the sample TCGA-56-1622 actually has genotype AB, appearing to be ‘copy loss’ relative the triploidy tumor genome (Supplementary Data Files 2–4). In the THetA2 prediction, the tumor genome is approximated as diploidy and has purity of 68% (21).

In short, the tumor clonal/subclonal with the unique SCNAs can be readily identified in the BubbleTree graph and the heuristic model is able to extend the manual inspection to an automated process with accuracy.

### Changes in SCNAs between hepatocellular carcinoma patients’ matched primary and metastatic or recurrent tumors

**Figure 6.**
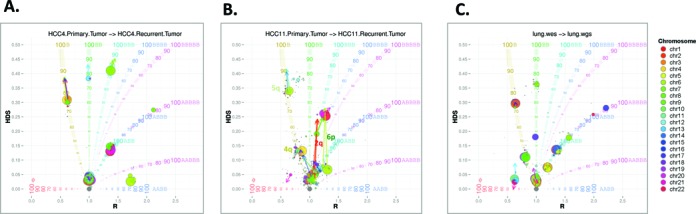
Tracking the alternations in clonality between primary and metastatic or recurrent tumors. Changes in the SCNA spectra from the primary tumor to the recurrent malignancy are illustrated by the arrows, for HCC patients (**A**) HCC4 and (**B**) HCC11; (A) shifts are all due to the change of the tumor purity from 76% to 86% and all SCNAs were conserved between these two tumor biopsies from patient HCC4. For patient HCC11, an interesting paradigm is displayed here: the dominant tumor clone (marked by the copy loss in chr5q) and subclone (marked by the copy loss in chr4q) are conserved between the primary tumor and the lung metastasis; many novel markers also occurred in the subclone (e.g. 2p and 6q). The exact copy number changes are more apparent in the genomic track plots (Supplementary Data File 4) and in Supplementary Data File 2; (**C**) The comparison between the DM-Lung WGS and WES data provides a good control for NGS data types of different coverage and depth, where few changes are highlighted (see Supplementary Data Files 2–4 for details).

Characterizing the tumor clonal composition over time can be a powerful method to better understand responses to treatment interventions, metastasis or recurrence, and general tumoral fitness. In this study, we procured specimens from hepatocellular carcinoma (HCC) patients experiencing tumor recurrence following orthotopic liver transplantation (OLT). One patient (HCC4) experienced tumor recurrence in the transplanted liver only seven months post-OLT, while another patient (HCC11) experienced tumor recurrence as a metastasis to the lung in 17 months following OLT. To evaluate the clonal evolution, we conducted WES on the primary and recurrent liver tumors as well as the peripheral blood of these two patients (Table 1). Shifts from primary to recurrent tumors in SCNAs are indicated with arrows pointing from the primary tumor to recurrent tumor in the BubbleTree graph (Figure 6). For HCC4, the arrows are all short and occur along the BubbleTree branches from 76% prevalence to 86% prevalence, suggesting that the SCNA spectra is identical between the primary and recurrent tumors and the minor differences in clonal spectrum are due to differences in the purities of the two tumor biopsies (Figure 6A). It is evident that the recurrent malignancy originated from the primary tumor of the recipient rather than from the donor, which was also confirmed by genetic comparison (data not shown).

For patient HCC11, the SCNA spectra between the primary and the lung metastasis are not so similar, as highlighted by the long arrows in the graph (Figure 6B). The two most significant alterations between the primary tumor and lung metastasis are LOH observed in chr2q and chr6p, resulting in two novel markers for the subclone with ∼40% prevalence (Figure 6B). This alteration in HDS values between the primary tumor and lung metastasis is more clearly illustrated in the genomic track plots (Supplementary Data File 4) and the text output (Supplementary Data File 2). It is noteworthy that both the clonal marker at chr5q and the subclonal marker at chr4q are remarkably conserved between the two longitudinal samples from HCC11. This suggests that the lung metastasis has a common ancestral origin and a *stable* subclonal structure as the primary neoplasm, where various novel SCNAs were acquired in the subclone through tumor progression. As a control, we found no significant difference between the double malignancy (DM)-Lung WGS and WES data (Figure 6C).

### Deconvoluting the chronology of acquired somatic single nucleotide variants (SNVs) or insertion/deletions (indels) and SCNAs along the clonal evolution

**Figure 7.**
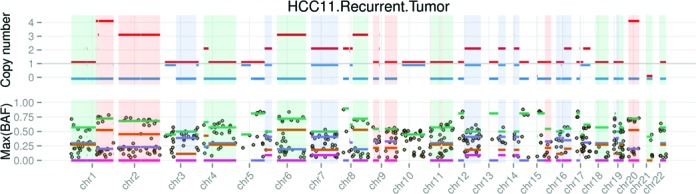
The allele frequencies of the somatic mutations can be explained by somatic copy number variations. The scheme for the first allele-specific copy number track is the same as Figure 3. In the second track, the horizontal bars indicate the expected somatic mutation frequencies calculated by the models described in Supplementary Figure S3, and dots represent detected somatic mutations. Assuming the parental allele B has more copy number than the parental allele A in a SCNA, the green bar represents the expect allele frequency of the somatic mutation occurring on the parental allele B prior to the dominant tumor clone (Supplementary Figure S3A) and the brown bar for the somatic mutations unique in the subclone only (Supplementary Figure S3B). In a similar way, the purple and red bars present the cases where the somatic mutation occurs to the parental allele A prior and post to the SCNA event, respectively (Supplementary Figure S3C and S3D). In the genomic segments marking for the dominant tumor clone, the subclone 1 does not exist and thus *p*_1_ = 0 (see Supplementary Figure S3). Consequently, the four bars are collapsed into two, that is, only the green and purple bars are visible. We might even further date a SCNA event. For example, few somatic mutations in chr2 were found to be subclone unique, suggesting that the temporal interval between the founding of the dominant tumor clone to that of the subclone be relatively short, and the SCNA in chr2 is not likely to be an recent event.

SCNAs which ubiquitously occur in solid tumors, not only alter the allele frequencies of the germline heterozygous loci, but also affect those somatic SNVs/indels. Alternations in the latter vary, depending on the order of the somatic event occurrence and copy number change. Given the allele-specific copy number predicted by BubbleTree, we can leverage clonal abundance and allele type to predict the likely BAF and determine the appropriate chronology in the clonal evolution for a somatic SNV (Supplementary Figure S3). In this study, the predicted BAF track for patient HCC11 clearly indicated that the BAFs of the somatic SNVs are not randomly distributed, but more or less affected by the SCNA events (Figure 7). Accordingly, we may infer that those somatic variations matching the maximum expected BAF are likely to occur prior to formation of the dominant tumor clone, and less BAF for those occurring late and existing only in the tumor subclonal populations (Supplementary Figure S3). This function could be valuable to determine the order of the occurrences the acquired somatic SNVs/indels, along with SCNA events, and distinguish the driver mutations from the recently acquired treatment resistant variants.

### BubbleTree consistency using WGS or WES data types

We evaluated the generalizability of BubbleTree for performance on both WES and WGS data types, as there is a clear difference in granularity in coverage between these two sequencing data types. The DM lung WGS and WES samples were used for this assessment (Table 1). Based on this assessment, we found no significant difference between the two samples in tumor purity, ploidy, or clonality estimates. Both were predicted to have purity of 74%, ploidy of 1.9 and a subclone with the prevalence of ∼40% (Supplementary Table S1; also see Figure 6C and the genomic track plots in Supplementary Data File 4). This consistency between NGS data of vastly different coverage and depth also confirms the robustness of BubbleTree.

It is noteworthy that the purity of the ovarian tumor sample from the DM patient is very low (∼30%), where the resolution on the SCNA spectra becomes low. As a result. we do not explore this further here.

## DISCUSSION

The BubbleTree model presented here is an intuitive representation of purity, allele-specific copy number, and clonality for human tumor specimens. This method displays the clonal composition within a tumor at the genomic segment level with allele-specific copy number – a granular quality that is not provided by other tools used in NGS data analysis. Further, these estimates can be obtained simply by manual inspection of the BubbleTree graph. For larger patient studies, we developed a heuristic model to automate the predictions and provide a more accurate estimate (than that provided by visual inspection). The robust performance of the BubbleTree framework is primarily attributed to the use of both ***R*** scores and BAFs of the heterozygous germline loci and the three-step implementation.

### BAFs of the heterozygous germline loci play a critical role with the copy ratio *R* scores

The programs ABSOLUTE (19) and AbsCN-seq (20) primarily rely on the ***R*** scores, with an optional component utilizing the BAFs of somatic mutations. As discussed previously, somatic mutation frequencies can be useful for purity estimates (Figure 7), though they are rare as compared to germline heterozygous loci, which can cause higher variation and lower accuracy. THetA2 does not rely on simultaneously BAFs and ***R*** scores, but implements a method to select the optimal prediction in the post-processing stage (21). ASCAT, primarily designed for single nucleotide polymorphism (SNP) array data, relies on both BAF and SCNA, but has no subclonal estimate considered in the model (18).

### The three-step implementation of BubbleTree is key to efficiency and robust estimates

The three-step approach solves the complexity in a divide-and-conquer process. *Step 1*, tumor ploidy and copy ratio adjustment initially occurs. This step is simple, but very important. After aligning the bubble leaves with the branches and predicting tumor ploidy, the computation can focus on segments close to the branches and ignore all others. Notably, THetA2 failed to predict high ploidy samples in both the simulated data sets and the TCGA sample TCGA-56-1622; performance might be improved with inclusion of this component. *Step 2*, the greatest source of computation lies in this second step, where the prevalence of the tumor (sub)clones is identified. In this step, we preferably chose large segments in the region B and weighted segments in other regions lower priority (Supplementary Figure S1). Most programs weight all segments equally in their algorithm, which would require significantly more CPU time and likely deviate to an incorrect estimate. *Step 3*, we extend and cover our prediction to all segments, which, again, saves significant time in the grid search. Consequently, BubbleTree search time on WGS data for various ploidy states up to decasomy is less than one minute, which is much more CPU-efficient compared to THetA2.

As described in Supplementary Methods, the number of subclones is determined by the unsupervised hierarchical cluster analysis on the prevalence values of the segments, with the default cutoff distance value of 0.2 (i.e. the minimum prevalence difference between any two of the subclones is 20%). With reduced cutoff distance value, the prevalence values are likely to be clustered into more groups and therefore more tumor subclones could be predicted, without taking extra computation cost.

### Tumor genome reconstruction

The recently published THetA2 outperformed ABSOLUTE, ASCAT and AbsCN-seq in our benchmark tests. In particular, THetA2 makes the unique prediction of the tumor genome reconstruction for each of the component tumor subclones. This is certainly a very useful, but also aggressive design, which might account for the extremely long CPU running time in our testing. In this study, we found that complex tumor clonality is not uncommon in the tumor samples tested, in particularly, low abundance tumor subclones, which are challenging to accurately identify. We strategically chose to only identify unique SCNA markers for the tumor clone and subclones rather than reconstruct the whole tumor genome. BubbleTree, however, predicts the allele-specific copy number, which is unavailable in THetA2.

### Application of BubbleTree in cancer research

NGS technology provides a powerful platform to capture a snapshot of the tumor evolution and explore tumor heterogeneity. The BubbleTree framework, presented here, provides an effective way to evaluate the tumor heterogeneity at different levels - from the tumor ploidy to the tumor sample purity to the prevalence of the subclone and allele specific copy numbers for a particular genomic segment. In particular, we demonstrate a method to track the changes in tumor clonality using longitudinal tumor biopsies. We also clearly illustrated that BAFs of the somatic SNVs are not randomly distributed and most could be explained by the SCNAs of the host genomic segments.

### Polyploidy imposes an identifiability issue

BAFs can improve the prediction, but is not able to address the identifiability issue caused by polyploidy, particular for the case of low tumor purity. In this case, additional sample information is required to make the proper prediction. BubbleTree relies on an implicit assumption that the tumor biopsy has high purity, and thereby seeks the alternative solution of ‘higher’ tumor purity with the high tumor ploidy. However, this default setting can be customized by users.

### Limitations and next steps for the BubbleTree framework

The BubbleTree model and graph were developed as an R package. The heuristic model demonstrates promising results in this benchmark study against other comparable tools. However, there is a limitation in our identification of *homologous* SCNAs - the CNVs were first identified by circular binary segmentation (CBS) using the ***R*** scores (by DNAcopy and ExomeCNV), then BAFs were used to discard segments with heterogeneous BAFs (see Materials and Methods). Ideally, homologous SCNV identification should be conducted using both BAFs and ***R*** scores simultaneously, as implemented in *PennCNV* (28). The resultant segments are thereby likely to be more homogeneous and more complete (as no heterogeneous regions would be removed). When multiple tumor subclones harbor different SCNAs over the same genomic segment, the segment may have allele-specific copy numbers as fractions rather than integers. In the BubbleTree graph, the segment may thus be positioned away from any branch. A more sophisticated algorithm may need to be developed to address this challenge. For example, coupling the BubbleTree prediction with the genome reconstruction from THetA2 might be a potential solution.

BubbleTree was demonstrated here using both WES and WGS data from matched tumor/normal biopsies, but this model could be also applicable to array comparative genomic hybridization (aCGH) and SNP array data as well. As many tumor samples have no matched normal samples, it is interesting to extend the BubbleTree algorithm to unpaired tumor samples using the algorithm developed in the software CNVnator (29). In principle, it can be further extended for the targeted sequencing data.

It may be also interesting to combine the BubbleTree framework with the algorithm proposed by Nik-Zainal *et al*. (22) to reconstruct the tumor evolution at a fine resolution, though this is beyond the scope of this study.

## CONCLUSIONS

BubbleTree is a simple but powerful method to characterize tumor clonal composition at high granularity and display results in a meaningful way. This approach improves upon current tools utilized for this purpose. The complicated tumor karyotype plays a critical role in the tumorigenesis and tumoral progression and is imperative to both prognosis and diagnosis of certain cancers. The BubbleTree approach shown here may help advance our understanding of this tumor karyotype and its significance in tumorigenesis.

The BubbleTree framework is freely available as an R package at Bioconductor. The datasets used in this study were also distributed together with the R package and all results presented in this paper and the supplementary data files are reproducible (https://www.bioconductor.org/packages/release/bioc/html/BubbleTree.html).

## SUPPLEMENTARY DATA

Supplementary Data are available at NAR Online.

SUPPLEMENTARY DATA
