# Supplementary material for: BubbleTree: an intuitive visualization to elucidate tumoral aneuploidy and clonality using next generation sequencing data
Source: Nucleic Acids Res. 2015 Nov 17;44(4):e38. doi: 10.1093/nar/gkv1102 (PMC4770205; doi:10.1093/nar/gkv1102)
Supplement: SUPPLEMENTARY DATA [file supp_44_4_e38__index.html]

BubbleTree: an intuitive visualization to elucidate tumoral aneuploidy and clonality using next generation sequencing data — SUPPLEMENTARY DATA 

# BubbleTree: an intuitive visualization to elucidate tumoral aneuploidy and clonality using next generation sequencing data

## SUPPLEMENTARY DATA

- SUPPLEMENTARY DATA
- SUPPLEMENTARY DATA
- SUPPLEMENTARY DATA
- SUPPLEMENTARY DATA
- SUPPLEMENTARY DATA
- SUPPLEMENTARY DATA
- SUPPLEMENTARY DATA
