# Supplementary material for: BubbleTree: an intuitive visualization to elucidate tumoral aneuploidy and clonality using next generation sequencing data
Source: Nucleic Acids Res. 2015 Nov 17;44(4):e38. doi: 10.1093/nar/gkv1102 (PMC4770205; doi:10.1093/nar/gkv1102)
Supplement: SUPPLEMENTARY DATA [file supp_gkv1102_nar-02609-met-n-2015-File011.docx]

**Supplementary Figure S1 - BubbleTree branches.** The plot scheme is similar as that of **Figure 1**. In this plot, all integer allele-specific copy numbers up to the decasomy are depicted as branches. The entire plot could be split into regions A-F, where region A represents the area around the root of the tree. Genomic segments will be plotted as bubble leaves according to the $\boldsymbol{R}$-HDS scores derived from the NGS data. The segments in the different regions bestow various power to the right prediction and accordingly we list the regions in the decreasing order as: B > D > F > C > E > A.

**Supplementary Figure S2 - The simulated normal genome and the tumor (sub)clones.**

**Supplementary Figure S3 - Expected BAF of the somatic mutations due to the specific SCNA.** Four scenarios A-D are described here, which are represented by the horizontal bars: green, brown, purple, and red, respectively, in **Figure 7.**

# Tables

**Supplementary Table S1 - Summary of the predictions of BubbleTree**

# Supplementary Data Files

## Supplementary Methods

## Supplementary Data File 1: The read counts of the NGS datasets used in this study

## Supplementary Data File 2: The text outputs of the automated BubbleTree prediction for all NGS datasets used in this study

## Supplementary Data File 3: The tree plots of the BubbleTree prediction for all the NGS datasets

## Supplementary Data File 4: The genomic track plots of the BubbleTree prediction for all NGS datasets used in this study
