# Supplementary material for: BubbleTree: an intuitive visualization to elucidate tumoral aneuploidy and clonality using next generation sequencing data
Source: Nucleic Acids Res. 2015 Nov 17;44(4):e38. doi: 10.1093/nar/gkv1102 (PMC4770205; doi:10.1093/nar/gkv1102)
Supplement: SUPPLEMENTARY DATA [file supp_gkv1102_nar-02609-met-n-2015-File016.docx]

**Supplementary Methods**

***DNA sequence read mapping and variant calling***

All sequence data were quality controlled for read counts, quality values, kmer usage, GC-content, and other relevant parameters within FastQC (v0.10.1). The reads were aligned to the human genome (UCSC hg19; Feb 2009 release; Genome Reference Consortium GRCh37) using Bowtie2 (v2.0.0-beta7). The alignments were further refined by the indel realignment and PCR duplicate removal using GATK (v2.3.4) and Picard (v1.85), respectively. SAMtools (v0.1.18; *3*) mpileup (Qphred>30 and mapping quality>30), coupled with VarScan2 (v2.3.2), was utilized to call both single nucleotide variants (SNVs) and insertion/deletions (indels) with parameters: variant minimum frequency>5%, VarScan2 p-value for variant calls (based on Fisher’s exact test) <0.01, minimum coverage=15, minimum reads=3, minimum average quality=20, minimum frequency for homozygote=0.8.

Variant calls were made for all samples in a batch call and exported as a Variant Call Format (VCF) (v4.1) file. Then variant call refinement was conducted to identify somatic SNVs and Indels, according to the following empirical rules:

1. Average Qphred of reference or variant (or both) >20 in normal and tumor,
2. P-value from Fisher’s exact test of read counts between normal/tumor and reference/variant<0.01,
3. Variant frequency (Vf) in normal<3%, and
4. Tumor Vf at least 5% greater than Vf of normal.

Variant effects on genes were then annotated using the program ANNOVAR (v2013-07-28).

***Automated BubbleTree approach***

## Similarly to the manual interpretation, the heuristic automated approach consists of three steps: 1) assign the tumor ploidy and adjust copy ratio score, 2) identify the prevalences of the tumor (sub)clones using the large genomic segments in the preferred region B (Supplementary Figure S1) and 3) predict allele-specific copy numbers and the prevalence to all segment in the end.

**Assigning the tumor ploidy and adjust copy ratio scores**

We start this step by the determination of “the central regions” around the copy ratio score of 1. If most of the central regions have significant LOH but HDS scores below 1/6, we will assign the sample as triploidy. Given *h* as the weighted median HDS score of the segments and the ABB state (i.e., x=1 and y=2), we can calculate the likely purity *p* from the equation (2) and then the consequent ***R*** score from the equation (1). For tetraploidy, there is no good way to distinct the tetraploidy from diploidy (**Figure 2D**), except the scenario that the diploidy sample has a high purity (e.g., *p* > 50%, as indicated by the high HDS score). By default, if most of the segments have the low HDS scores (the empirically cutoff value is 0.15), we will assign the sample tetraploidy; otherwise, diploidy. The minor adjustment is further calculated after the tumor ploidy assignment, so as to minimize the gaps between bubbles to branches. Then, the ***R*** score (not the HDS score) of each segment is updated accordingly (**Figure 2**).

**Determining the prevalences of the tumor clones using the large segments**

There are at least two advantages to focus on the large segments in this step: 1) the ***R*** and HDS scores of large segments are less sensible to measurement noise; 2) it will save much of the computation time compared to search all the segments, for the number of the small segments is typically much larger than that of the large segments. We normalize each segment by the total segment length so that the same setting could be applicable to both WGS and WES data. For example, we may select the large segments as above 0.1% of the total length. Then, we try to identify the best solution (*x*,*y*,*p*) for each segment by the grid search, where the *p* score steps by 1% from 15 to 100%. As mentioned in the **Method** section of the main text, we preferably select the large segments located the region B (**Supplementary Figure S1**), and then we group the derived *p* values by unsupervised hierarchical clustering to obtain the likely prevalence scores of the tumor (sub)clones, with the default cutoff distance value of 0.2. We further check those segments in the regions D and F (**Supplementary Figure S1**), and add the prevalence if it has not been covered.

**Assigning allele-specific copy numbers and the prevalence to each segment**

We make another round grid search to assign (*x*,*y*,*p*) to all the segments (including small segments) using the resultant prevalence scores from the previous step and all the likely values of x and y. We also calculate the projected ***R*** and HDS scores based on the values of *x*, *y*, and *p*. The deviation from the project scores to the measured scores may indicate out of fitness for the specific segment. The exact process of these three steps is available in the freely distributed R codes (https://www.bioconductor.org/packages/release/bioc/html/BubbleTree.html).

## 
