# Supplementary material for: BubbleTree: an intuitive visualization to elucidate tumoral aneuploidy and clonality using next generation sequencing data
Source: Nucleic Acids Res. 2015 Nov 17;44(4):e38. doi: 10.1093/nar/gkv1102 (PMC4770205; doi:10.1093/nar/gkv1102)
Supplement: SUPPLEMENTARY DATA [file supp_gkv1102_nar-02609-met-n-2015-File019.pdf]

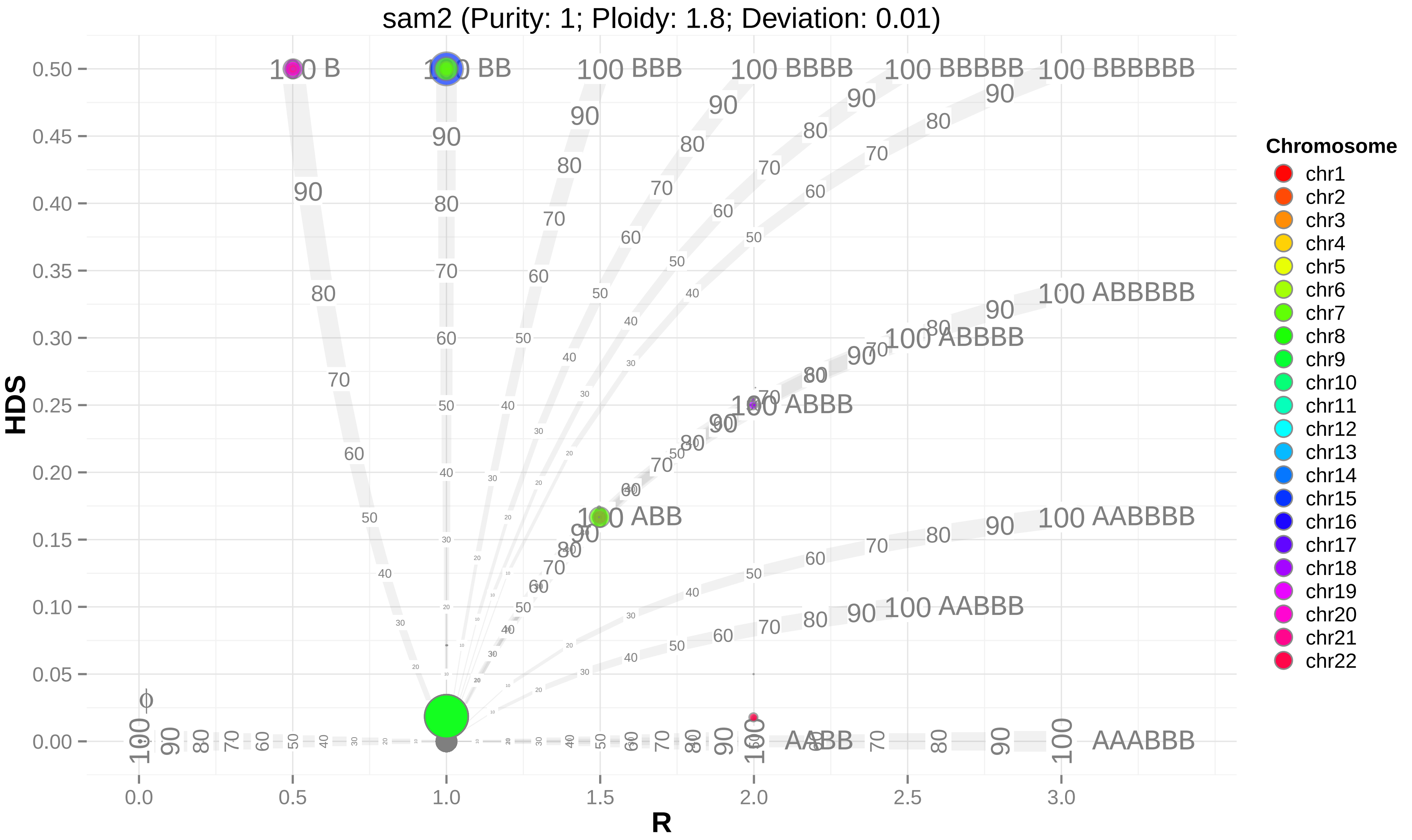
sam2 (Purity: 1; Ploidy: 1.8; Deviation: 0.01)
HDS
R
100 B
100 BB
100 BBB
100 BBBB
100 BBBBB
100 BBBBBB
100 ABBBBB
100 ABBBB
100 ABBB
100 ABB
100 AABBBB
100 AABBB
AABB
AAABBB
ϕ
Chromosome
chr1
chr2
chr3
chr4
chr5
chr6
chr7
chr8
chr9
chr10
chr11
chr12
chr13
chr14
chr15
chr16
chr17
chr18
chr19
chr20
chr21
chr22

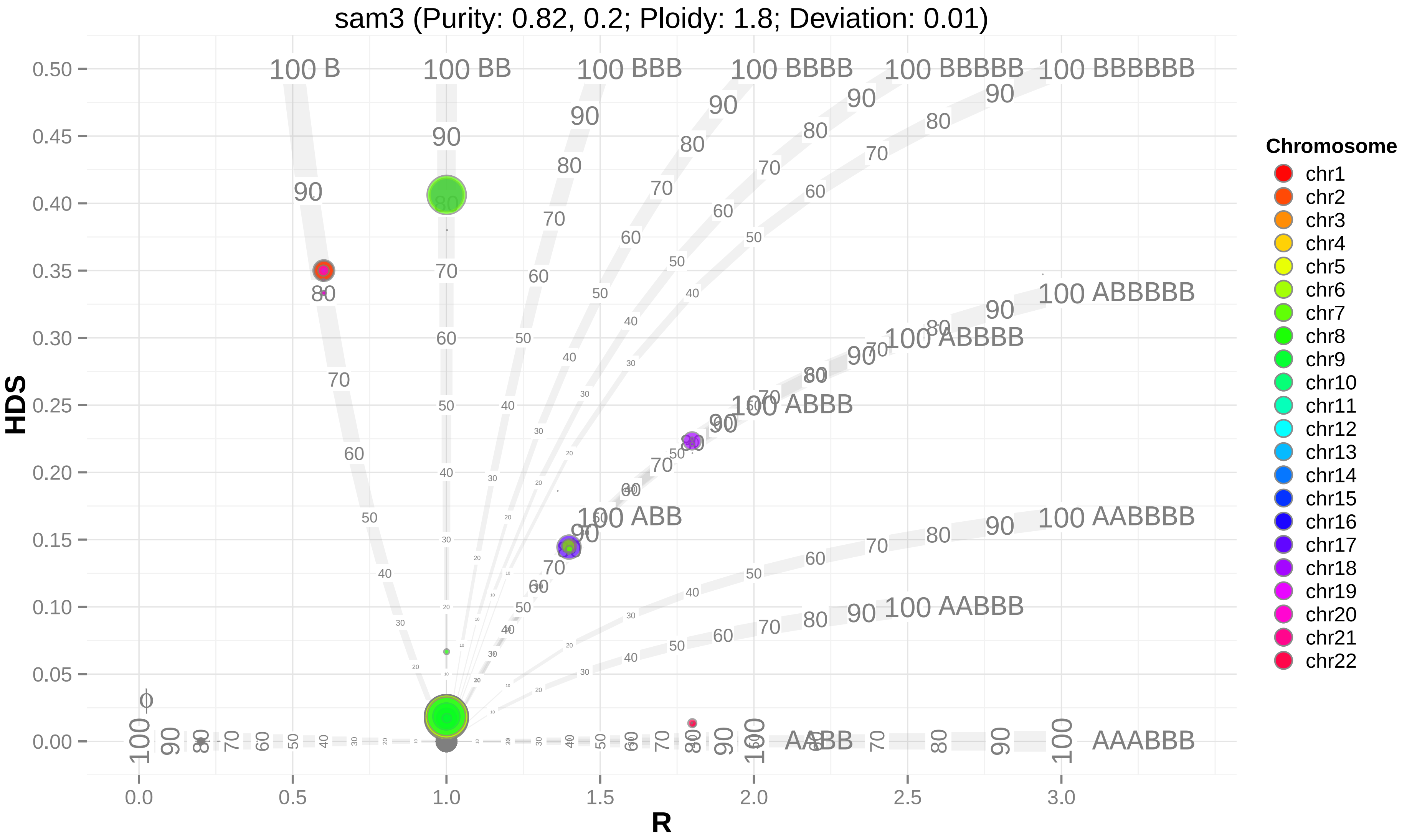
sam3 (Purity: 0.82, 0.2; Ploidy: 1.8; Deviation: 0.01)
HDS
R
100 B
100 BB
100 BBB
100 BBBB
100 BBBBB
100 BBBBBB
100 ABBBBB
100 ABBBB
100 ABBB
100 ABB
100 AABBBB
100 AABBB
AABB
AAABBB
Chromosome
chr1
chr2
chr3
chr4
chr5
chr6
chr7
chr8
chr9
chr10
chr11
chr12
chr13
chr14
chr15
chr16
chr17
chr18
chr19
chr20
chr21
chr22

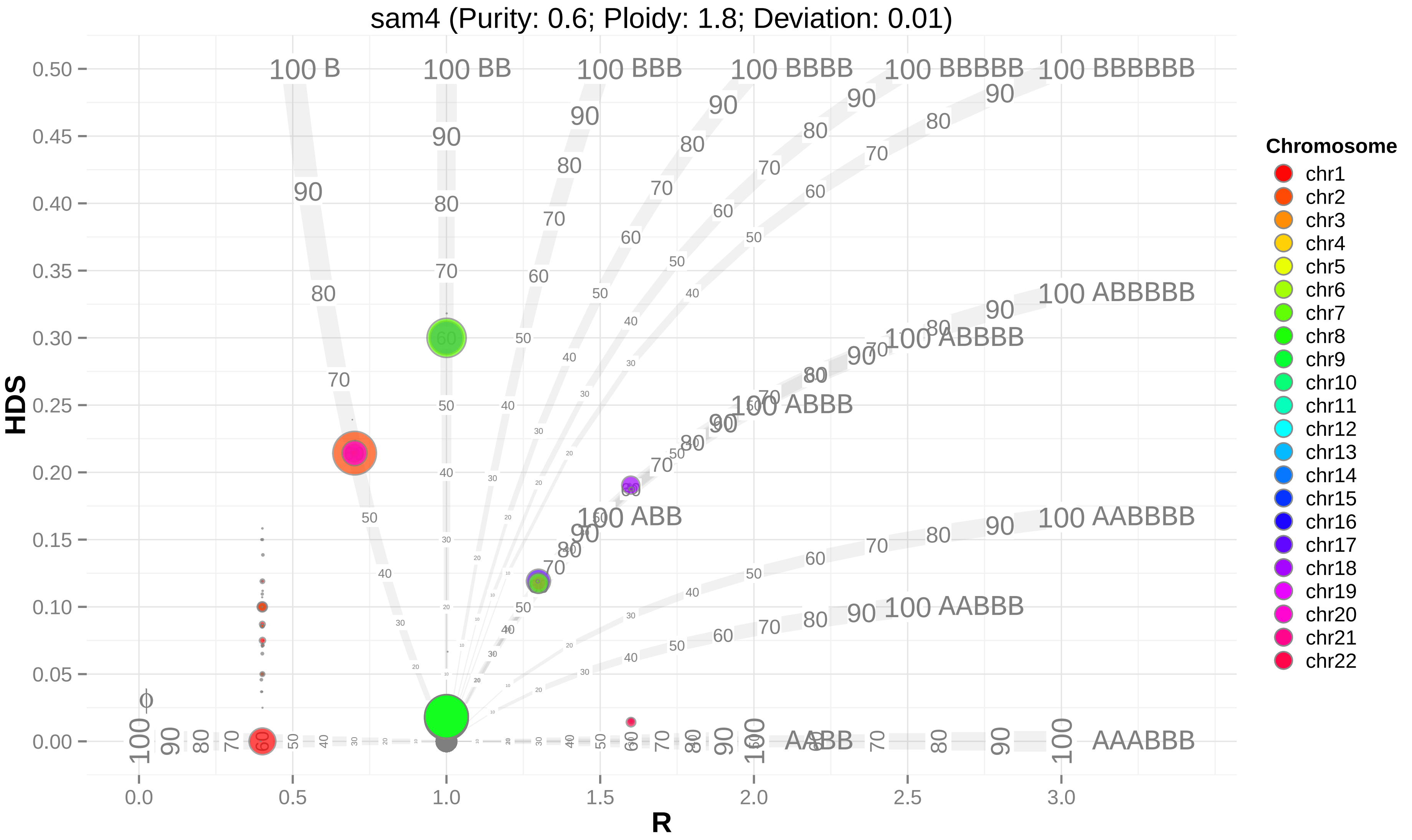
sam4 (Purity: 0.6; Ploidy: 1.8; Deviation: 0.01)
HDS
R
Chromosome
chr1
chr2
chr3
chr4
chr5
chr6
chr7
chr8
chr9
chr10
chr11
chr12
chr13
chr14
chr15
chr16
chr17
chr18
chr19
chr20
chr21
chr22
100 B
100 BB
100 BBB
100 BBBB
100 BBBBB
100 BBBBBB
100 ABBBBB
100 ABBBB
100 ABBB
100 ABB
100 AABBBB
100 AABBB
AABB
AAABBB
φ

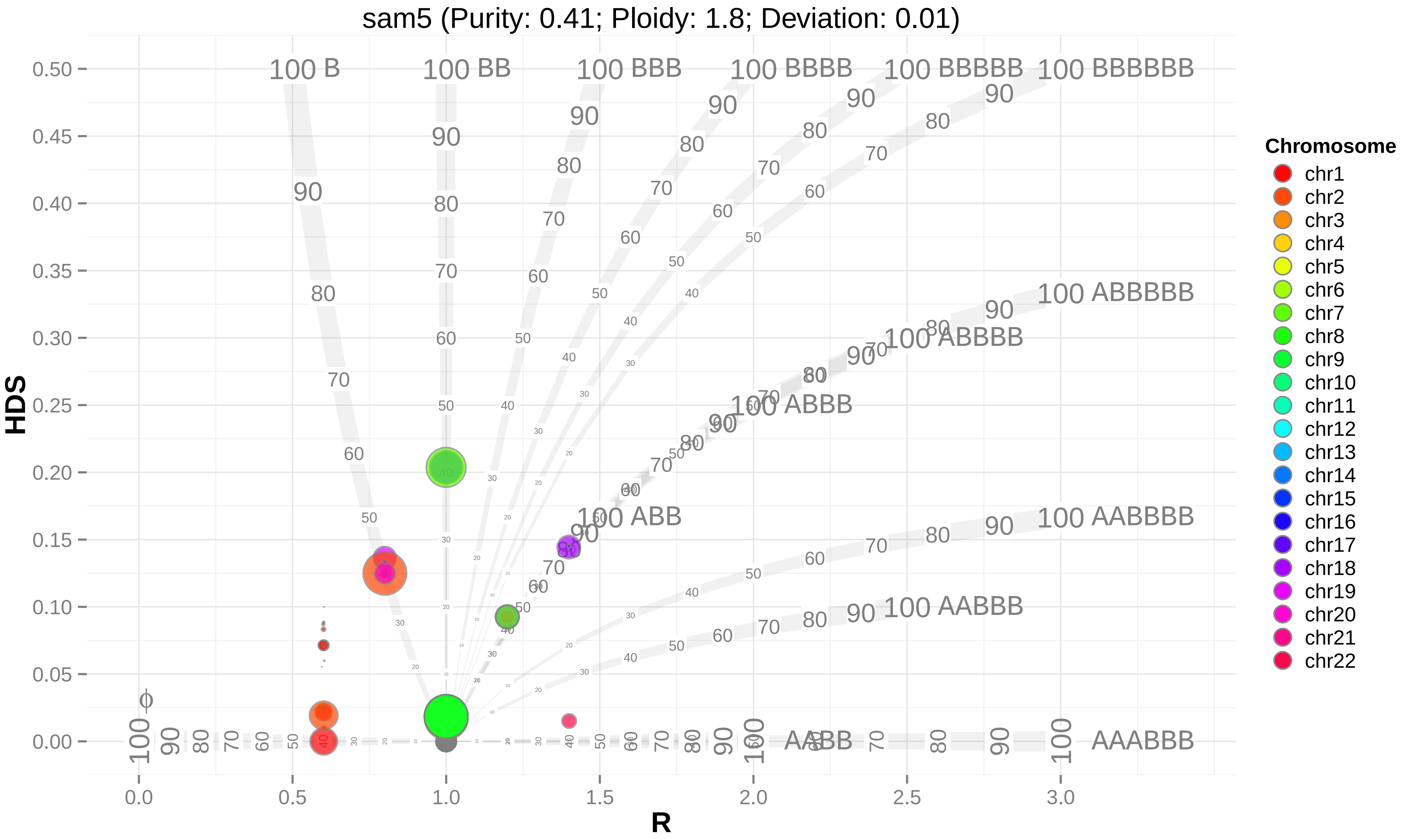
sam5 (Purity: 0.41; Ploidy: 1.8; Deviation: 0.01)
HDS
R
0.00
0.05
0.10
0.15
0.20
0.25
0.30
0.35
0.40
0.45
0.50
0.0
0.5
1.0
1.5
2.0
2.5
3.0
100 B
100 BB
100 BBB
100 BBBB
100 BBBBB
100 BBBBBB
100 ABBBBB
100 ABBBB
100 ABBB
100 ABB
100 AABBBB
100 AABBB
AABB
AAABBB
ϕ
Chromosome
chr1
chr2
chr3
chr4
chr5
chr6
chr7
chr8
chr9
chr10
chr11
chr12
chr13
chr14
chr15
chr16
chr17
chr18
chr19
chr20
chr21
chr22

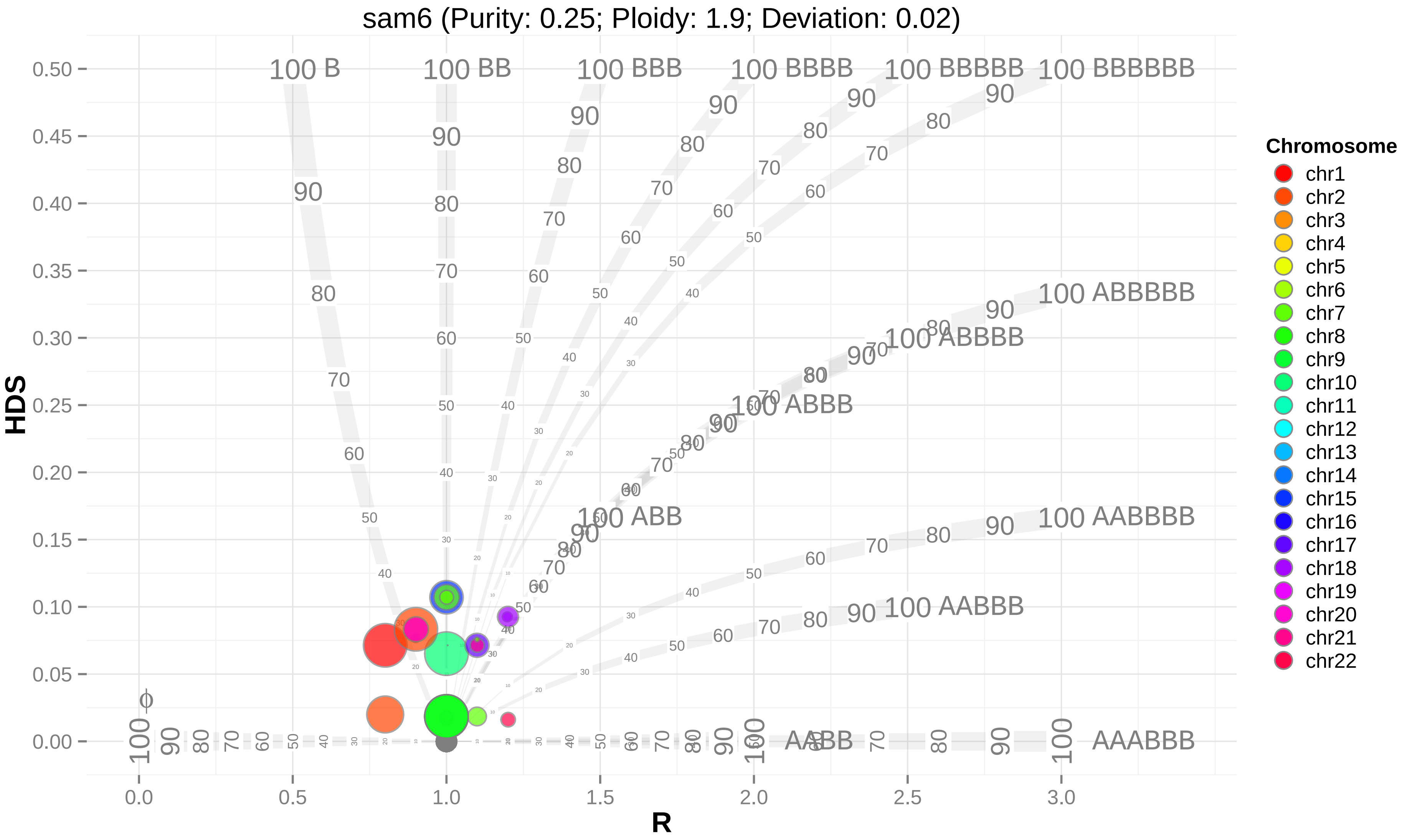

sam6 (Purity: 0.25; Ploidy: 1.9; Deviation: 0.02)
HDS
R
100 B
100 BB
100 BBB
100 BBBB
100 BBBBB
100 BBBBBB
100 ABBBBB
100 ABBBB
100 ABBB
100 ABB
100 AABBBB
100 AABBB
AABB
AAABBB
Chromosome
chr1
chr2
chr3
chr4
chr5
chr6
chr7
chr8
chr9
chr10
chr11
chr12
chr13
chr14
chr15
chr16
chr17
chr18
chr19
chr20
chr21
chr22

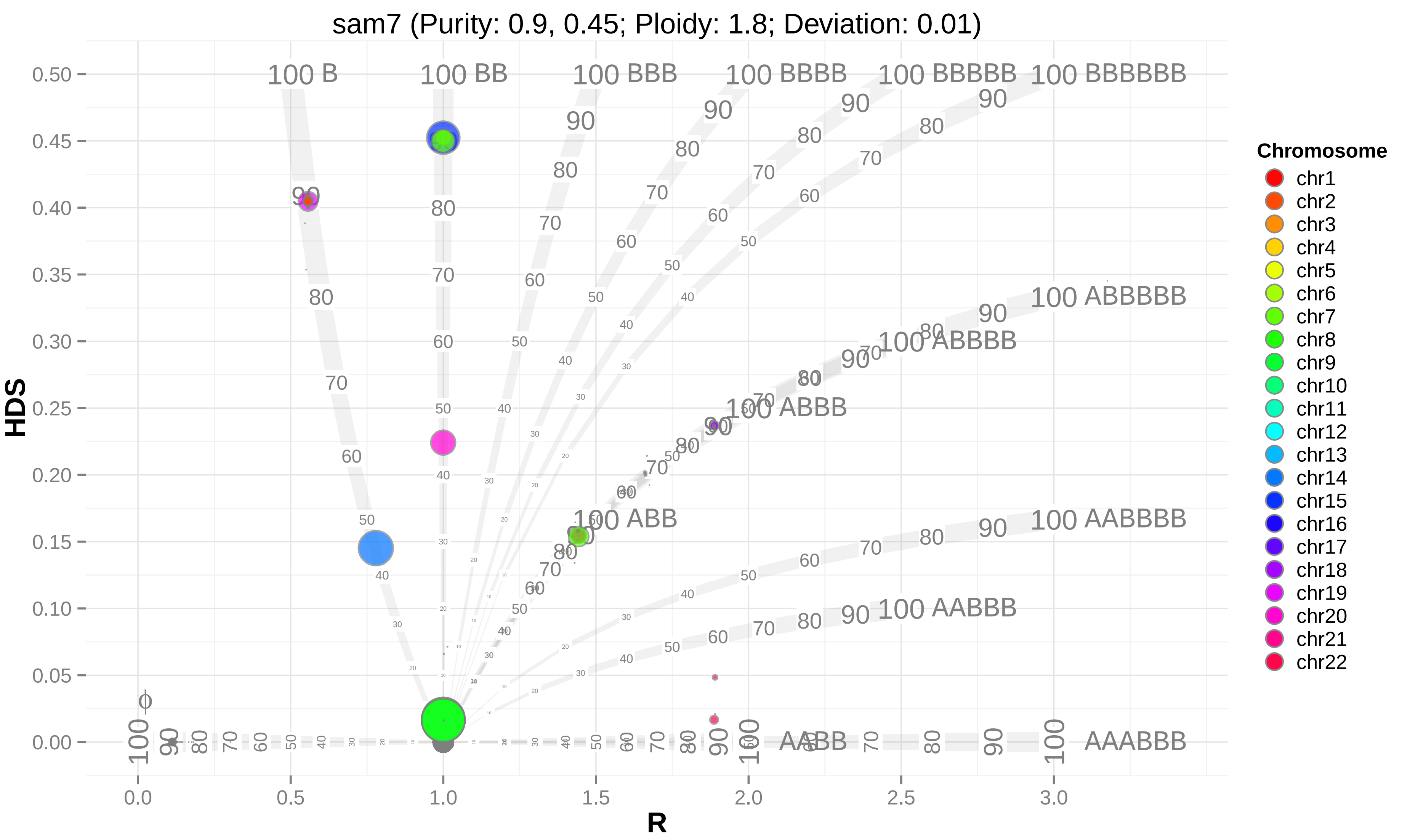
sam7 (Purity: 0.9, 0.45; Ploidy: 1.8; Deviation: 0.01)
HDS
R
Chromosome
chr1
chr2
chr3
chr4
chr5
chr6
chr7
chr8
chr9
chr10
chr11
chr12
chr13
chr14
chr15
chr16
chr17
chr18
chr19
chr20
chr21
chr22
100 B
100 BB
100 BBB
100 BBBB
100 BBBBB
100 BBBBBB
100 ABBBBB
100 ABBBB
100 ABBB
100 ABB
100 AABBBB
100 AABBB
AABB
AAABBB
ϕ

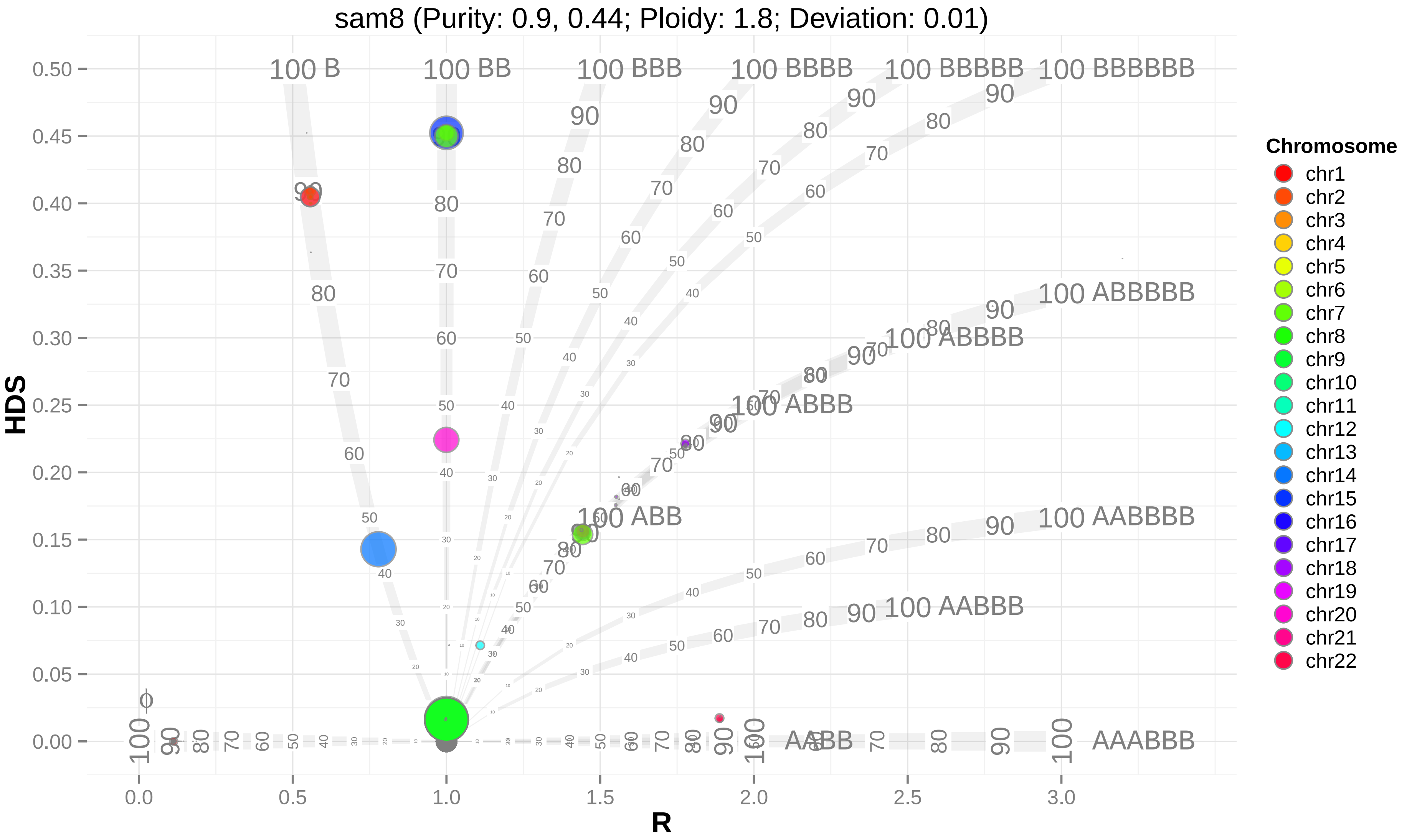

sam8 (Purity: 0.9, 0.44; Ploidy: 1.8; Deviation: 0.01)
HDS
R
100 B
100 BB
100 BBB
100 BBBB
100 BBBBB
100 BBBBBB
100 ABBBBB
100 ABBBB
100 ABBB
100 ABB
100 AABBBB
100 AABBB
AABB
AAABBB
ϕ
Chromosome
chr1
chr2
chr3
chr4
chr5
chr6
chr7
chr8
chr9
chr10
chr11
chr12
chr13
chr14
chr15
chr16
chr17
chr18
chr19
chr20
chr21
chr22

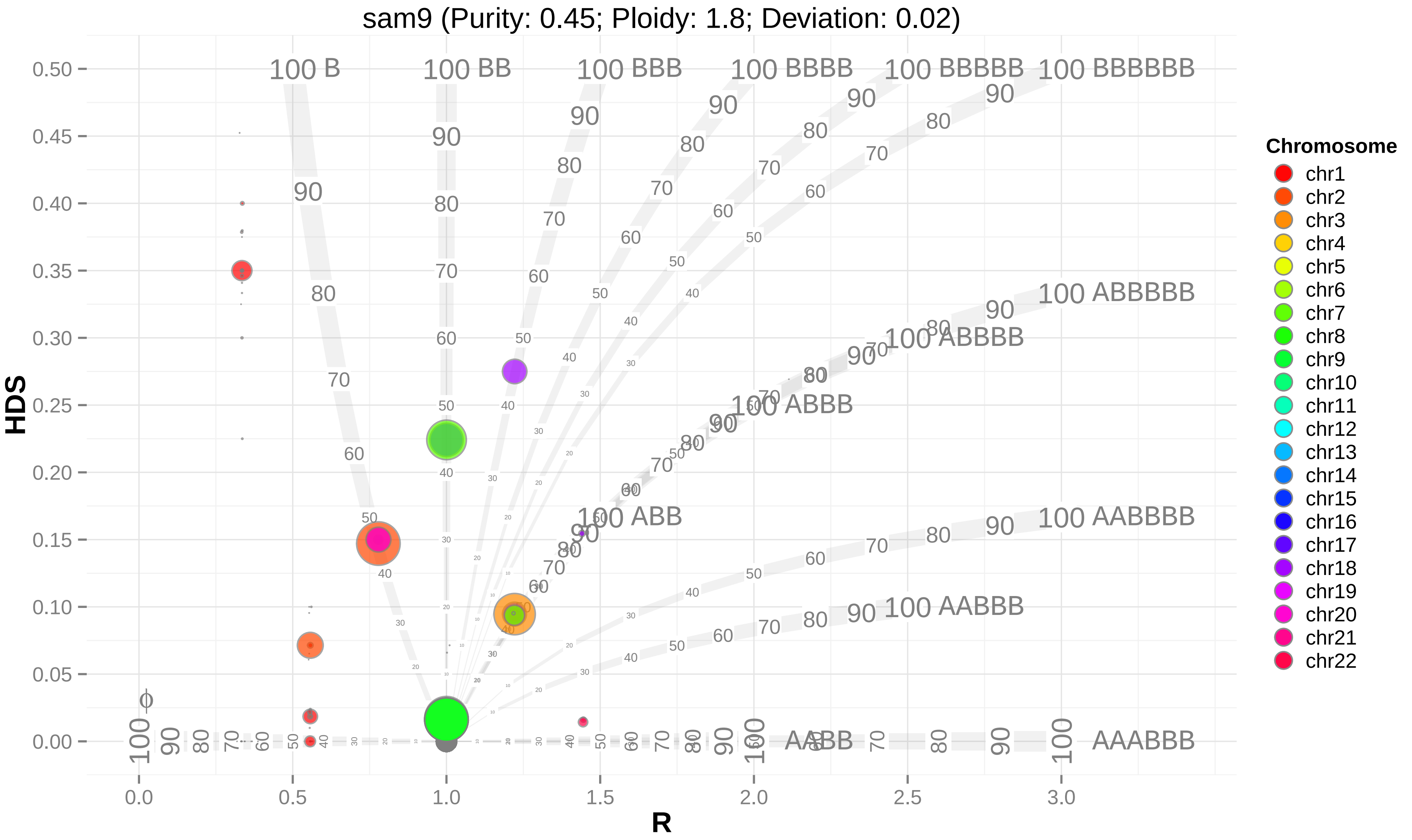
sam9 (Purity: 0.45; Ploidy: 1.8; Deviation: 0.02)
HDS
R
0.00
0.05
0.10
0.15
0.20
0.25
0.30
0.35
0.40
0.45
0.50
0.0
0.5
1.0
1.5
2.0
2.5
3.0
100 B
100 BB
100 BBB
100 BBBB
100 BBBBB
100 BBBBBB
100 ABBBBB
100 ABBBB
100 ABBB
100 ABB
100 AABBBB
100 AABBB
AABB
AAABBB
ϕ
Chromosome
chr1
chr2
chr3
chr4
chr5
chr6
chr7
chr8
chr9
chr10
chr11
chr12
chr13
chr14
chr15
chr16
chr17
chr18
chr19
chr20
chr21
chr22

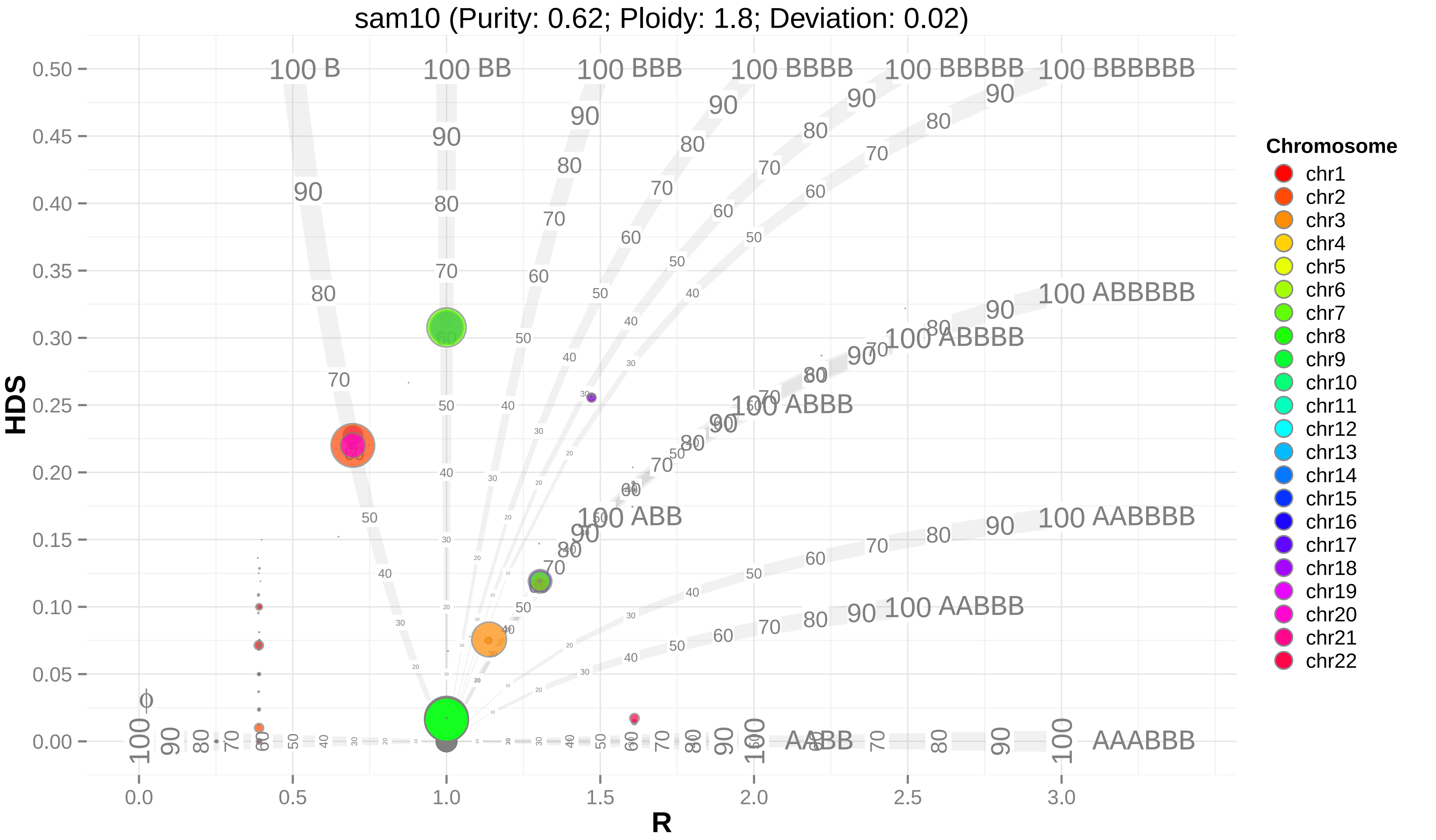
sam10 (Purity: 0.62; Ploidy: 1.8; Deviation: 0.02)
HDS
R
Chromosome
chr1
chr2
chr3
chr4
chr5
chr6
chr7
chr8
chr9
chr10
chr11
chr12
chr13
chr14
chr15
chr16
chr17
chr18
chr19
chr20
chr21
chr22
100 B
100 BB
100 BBB
100 BBBB
100 BBBBB
100 BBBBBB
100 ABBBBB
100 ABBBB
100 ABBB
100 ABB
100 AABBBB
100 AABBB
AABB
AAABBB

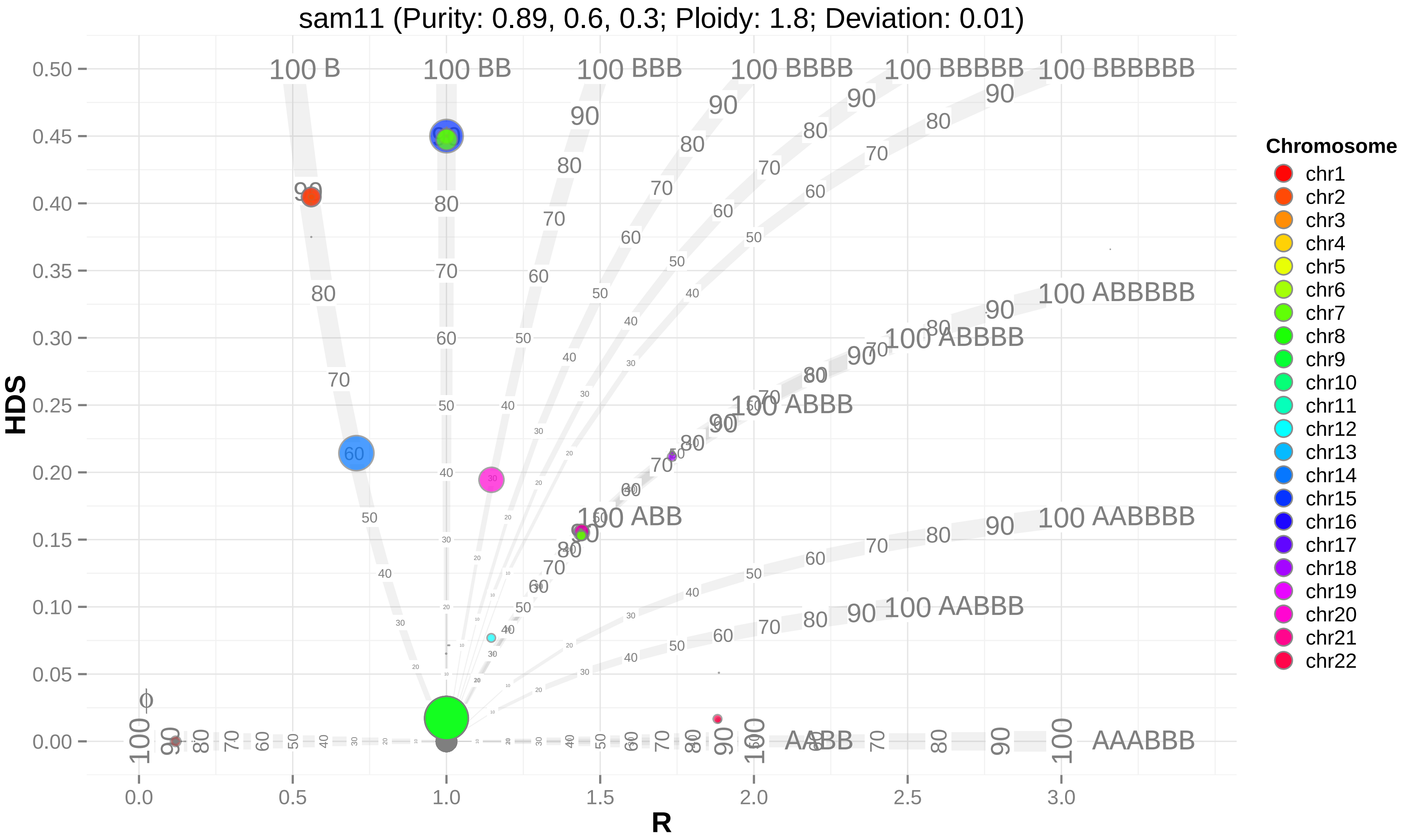
sam11 (Purity: 0.89, 0.6, 0.3; Ploidy: 1.8; Deviation: 0.01)
HDS
R
Chromosome
chr1
chr2
chr3
chr4
chr5
chr6
chr7
chr8
chr9
chr10
chr11
chr12
chr13
chr14
chr15
chr16
chr17
chr18
chr19
chr20
chr21
chr22
100 B
100 BB
100 BBB
100 BBBB
100 BBBBB
100 BBBBBB
100 ABBBBB
100 ABBBB
100 ABBB
100 ABB
100 AABBBB
100 AABBB
AABB
AAABBB

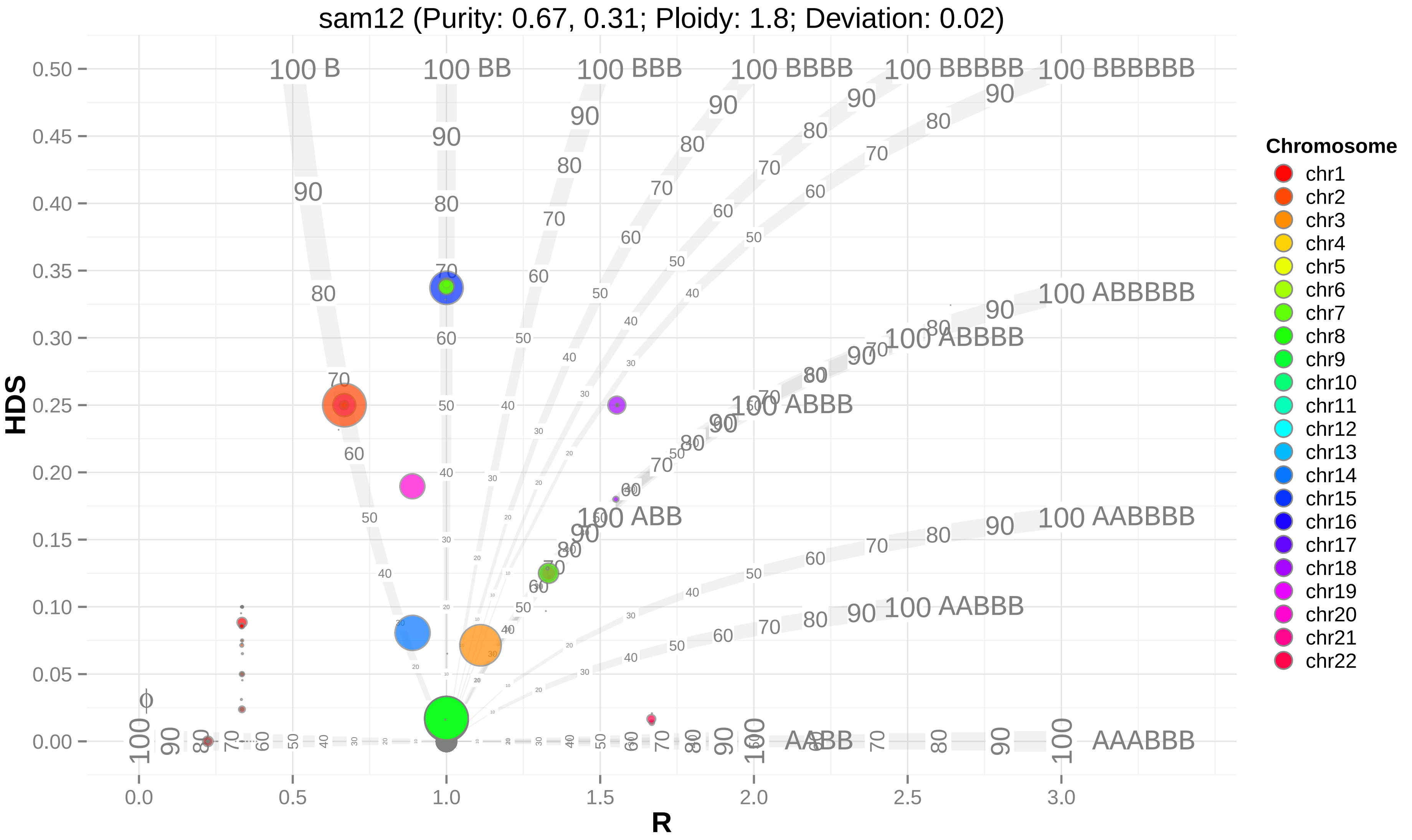
sam12 (Purity: 0.67, 0.31; Ploidy: 1.8; Deviation: 0.02)
HDS
R
Chromosome
chr1
chr2
chr3
chr4
chr5
chr6
chr7
chr8
chr9
chr10
chr11
chr12
chr13
chr14
chr15
chr16
chr17
chr18
chr19
chr20
chr21
chr22
100 B
100 BB
100 BBB
100 BBBB
100 BBBBB
100 BBBBBB
100 ABBBBB
100 ABBBB
100 ABBB
100 ABB
100 AABBBB
100 AABBB
AABB
AAABBB
ϕ

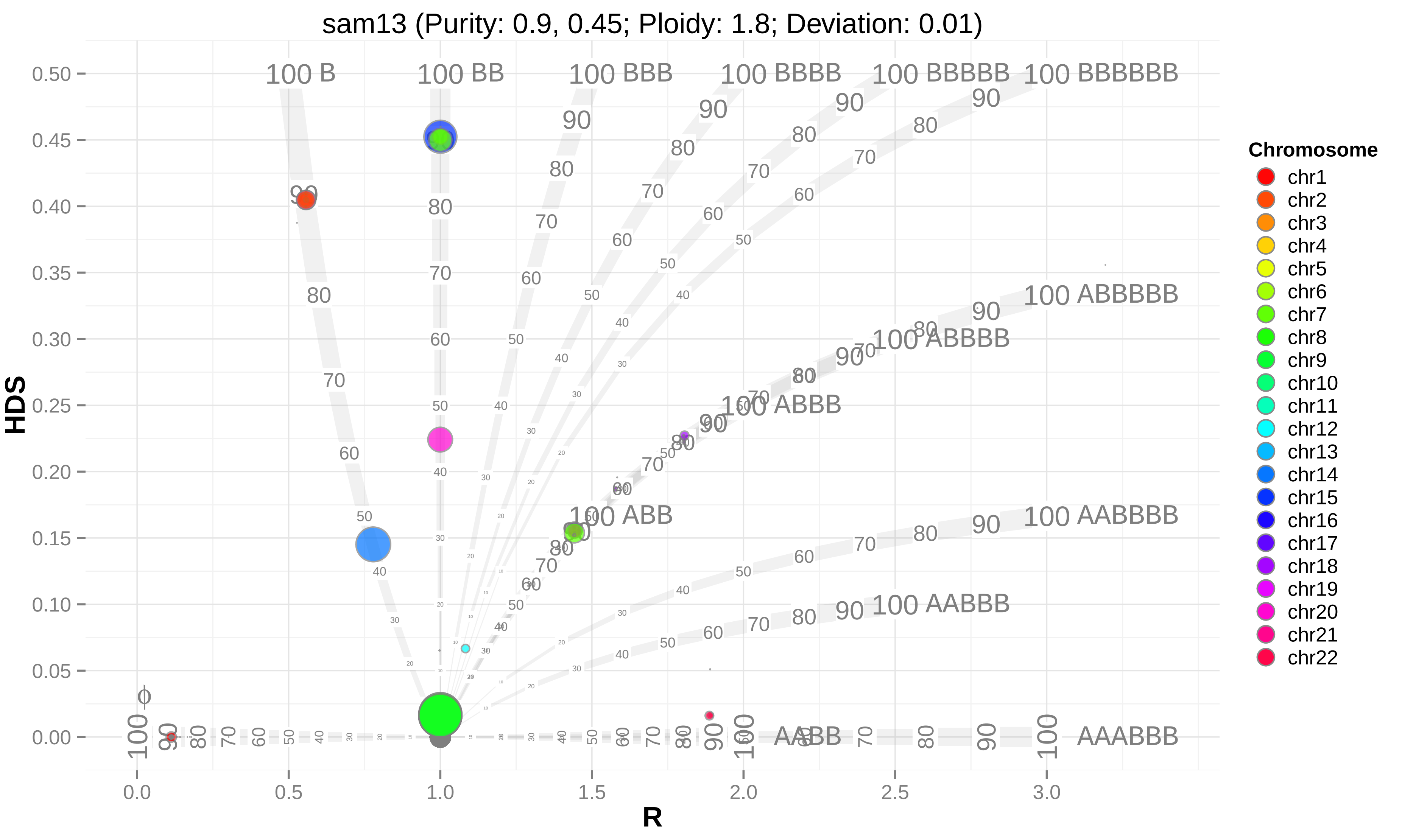
sam13 (Purity: 0.9, 0.45; Ploidy: 1.8; Deviation: 0.01)
HDS
R
100 B
100 BB
100 BBB
100 BBBB
100 BBBBB
100 BBBBBB
100 ABBBBB
100 ABBBB
100 ABBB
100 ABB
100 AABBBB
100 AABBB
AABB
AAABBB
ϕ
Chromosome
chr1
chr2
chr3
chr4
chr5
chr6
chr7
chr8
chr9
chr10
chr11
chr12
chr13
chr14
chr15
chr16
chr17
chr18
chr19
chr20
chr21
chr22

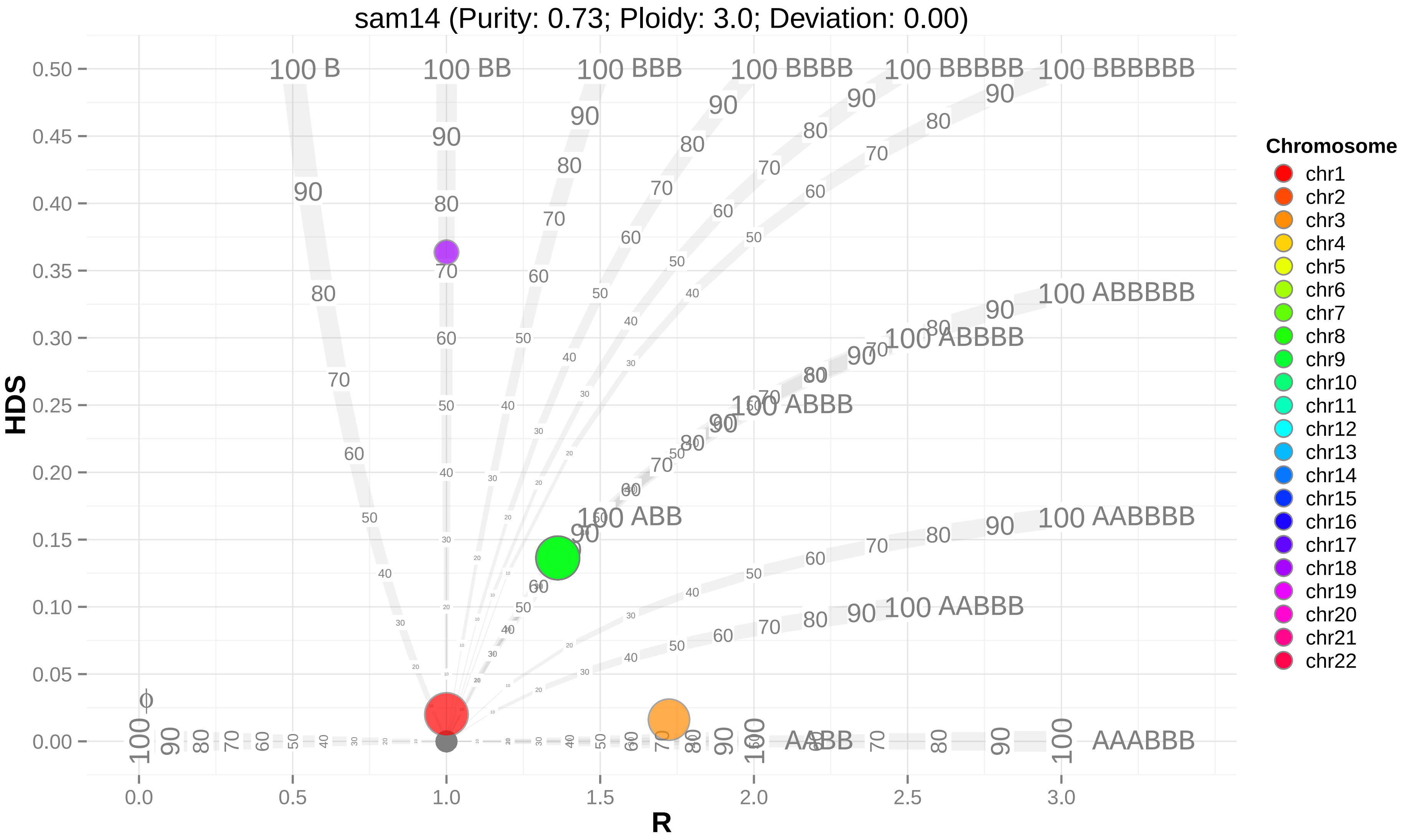

sam14 (Purity: 0.73; Ploidy: 3.0; Deviation: 0.00)
HDS
R
Chromosome
chr1
chr2
chr3
chr4
chr5
chr6
chr7
chr8
chr9
chr10
chr11
chr12
chr13
chr14
chr15
chr16
chr17
chr18
chr19
chr20
chr21
chr22
100 B
100 BB
100 BBB
100 BBBB
100 BBBBB
100 BBBBBB
100 ABBBBB
100 ABBBB
100 ABBB
100 ABB
100 AABBBB
100 AABBB
AABB
AAABBB

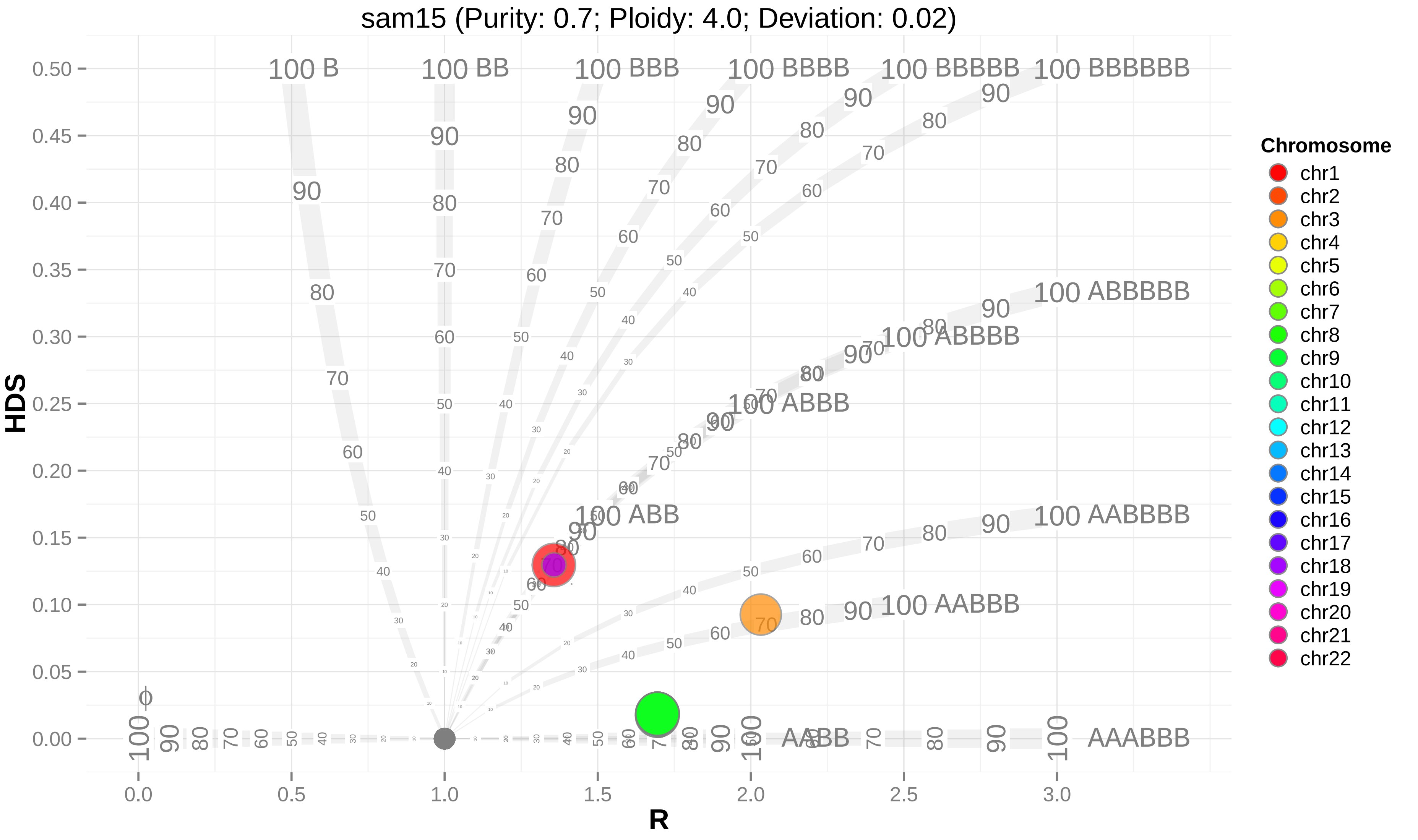
sam15 (Purity: 0.7; Ploidy: 4.0; Deviation: 0.02)
HDS
R
Chromosome
chr1
chr2
chr3
chr4
chr5
chr6
chr7
chr8
chr9
chr10
chr11
chr12
chr13
chr14
chr15
chr16
chr17
chr18
chr19
chr20
chr21
chr22
100 B
100 BB
100 BBB
100 BBBB
100 BBBBB
100 BBBBBB
100 ABBBBB
100 ABBBB
100 ABBB
100 ABB
100 AABBBB
100 AABBB
AABB
AAABBB

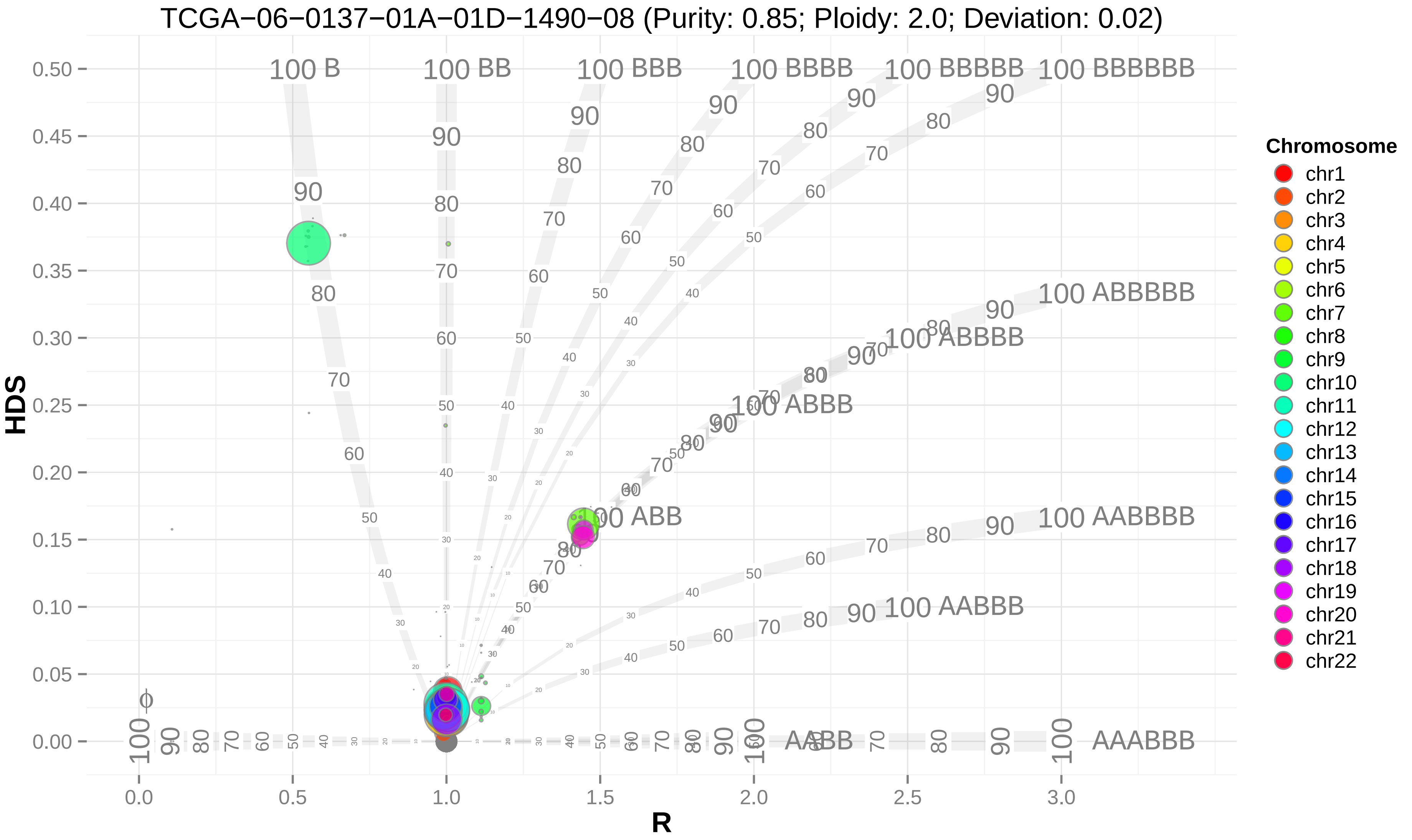

TCGA−06−0137−01A−01D−1490−08 (Purity: 0.85; Ploidy: 2.0; Deviation: 0.02)
HDS
R
Chromosome
chr1
chr2
chr3
chr4
chr5
chr6
chr7
chr8
chr9
chr10
chr11
chr12
chr13
chr14
chr15
chr16
chr17
chr18
chr19
chr20
chr21
chr22
100 B
100 BB
100 BBB
100 BBBB
100 BBBBB
100 BBBBBB
100 ABBBBB
100 ABBBB
100 ABBB
100 AABBBB
100 AABBB
AABB
AAABBB

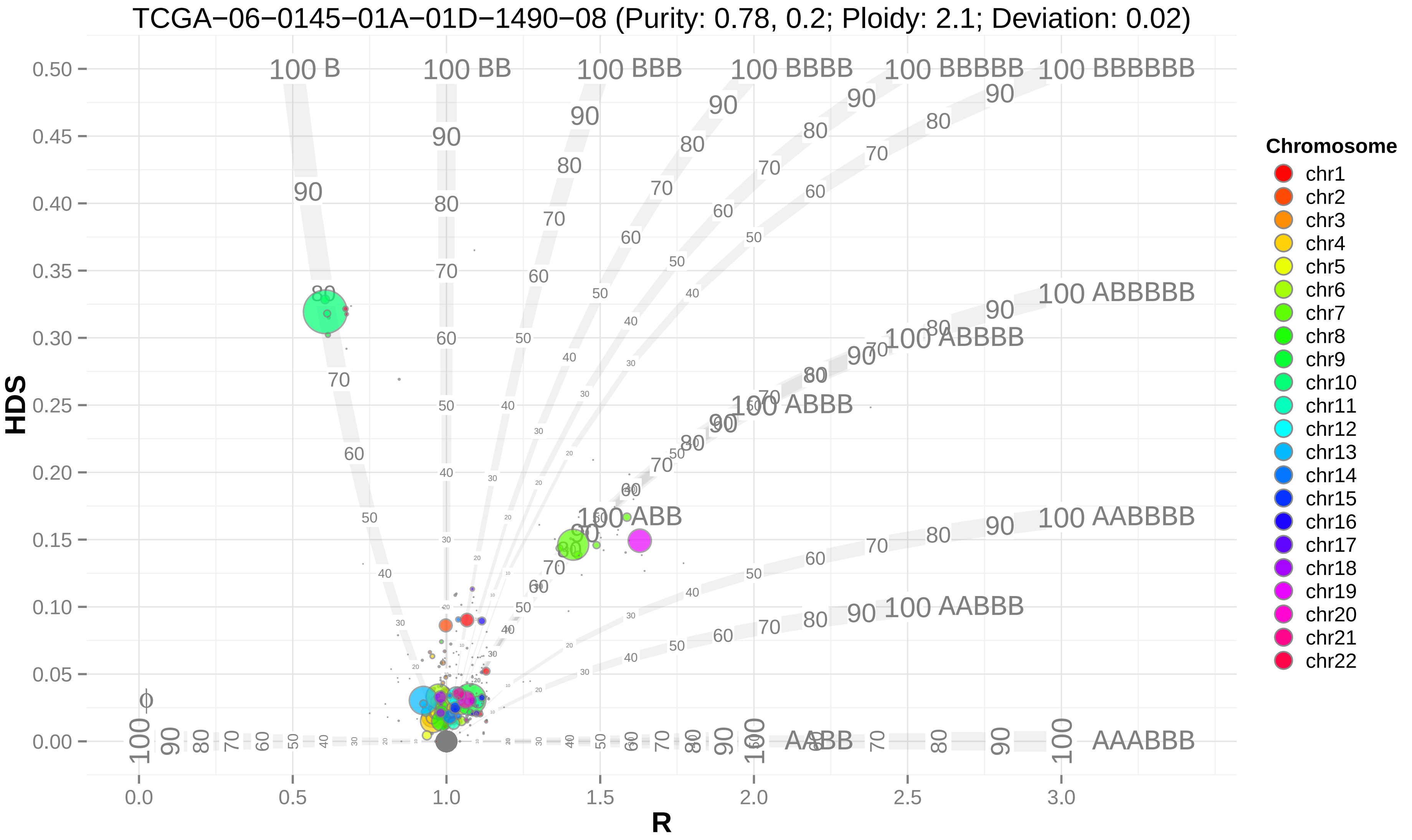
TCGA−06−0145−01A−01D−1490−08 (Purity: 0.78, 0.2; Ploidy: 2.1; Deviation: 0.02)
HDS
R
100 B
100 BB
100 BBB
100 BBBB
100 BBBBB
100 BBBBBB
100 ABBBBB
100 ABBBB
100 ABBB
100 ABB
100 AABBBB
100 AABBB
AABB
AAABBB
Chromosome
chr1
chr2
chr3
chr4
chr5
chr6
chr7
chr8
chr9
chr10
chr11
chr12
chr13
chr14
chr15
chr16
chr17
chr18
chr19
chr20
chr21
chr22

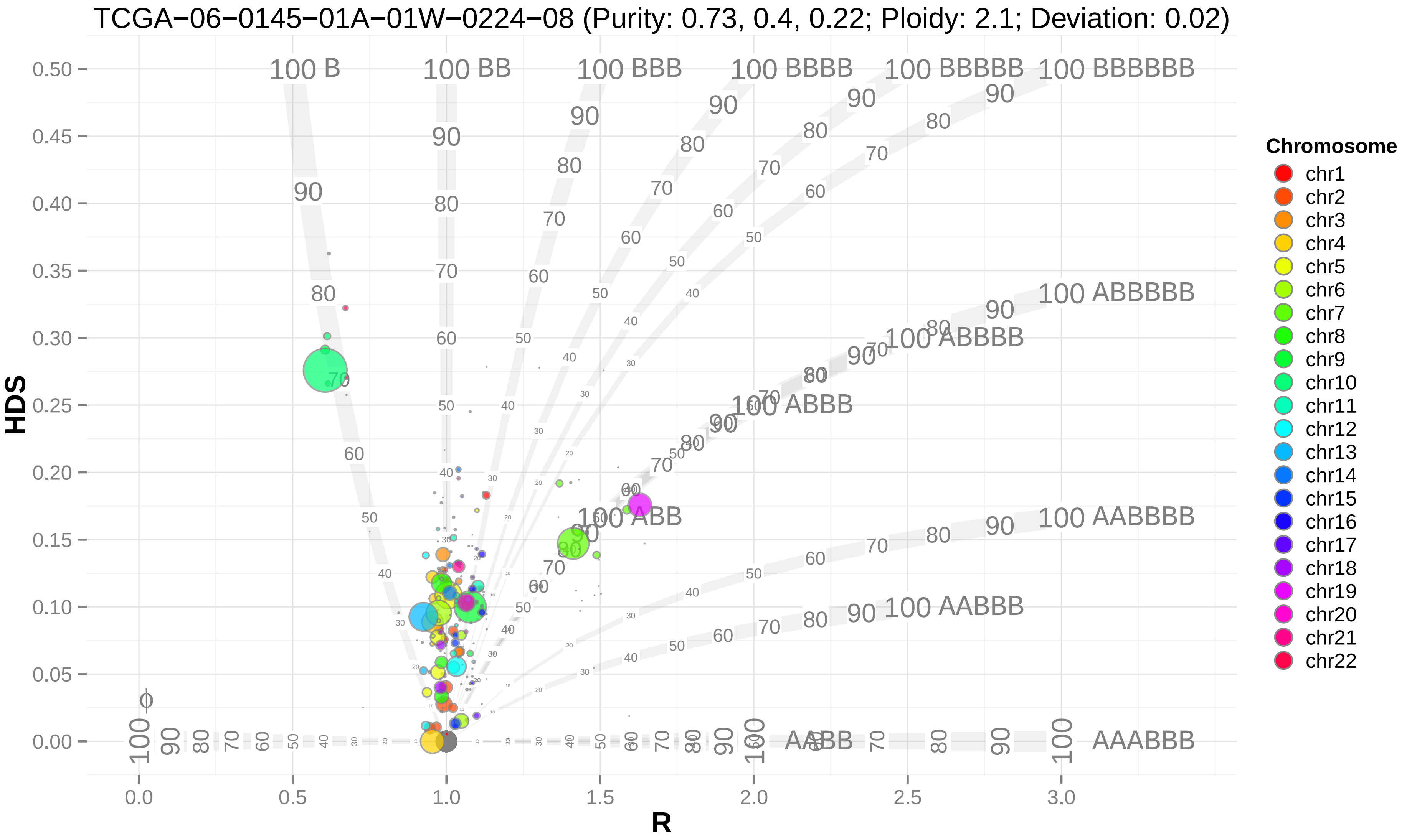

TCGA−06−0145−01A−01W−0224−08 (Purity: 0.73, 0.4, 0.22; Ploidy: 2.1; Deviation: 0.02)
HDS
R
Chromosome
chr1
chr2
chr3
chr4
chr5
chr6
chr7
chr8
chr9
chr10
chr11
chr12
chr13
chr14
chr15
chr16
chr17
chr18
chr19
chr20
chr21
chr22
100 B
100 BB
100 BBB
100 BBBB
100 BBBBB
100 BBBBBB
100 ABBBBB
100 ABBBB
100 ABBB
100 ABB
100 AABBBB
100 AABBB
AABB
AAABBB

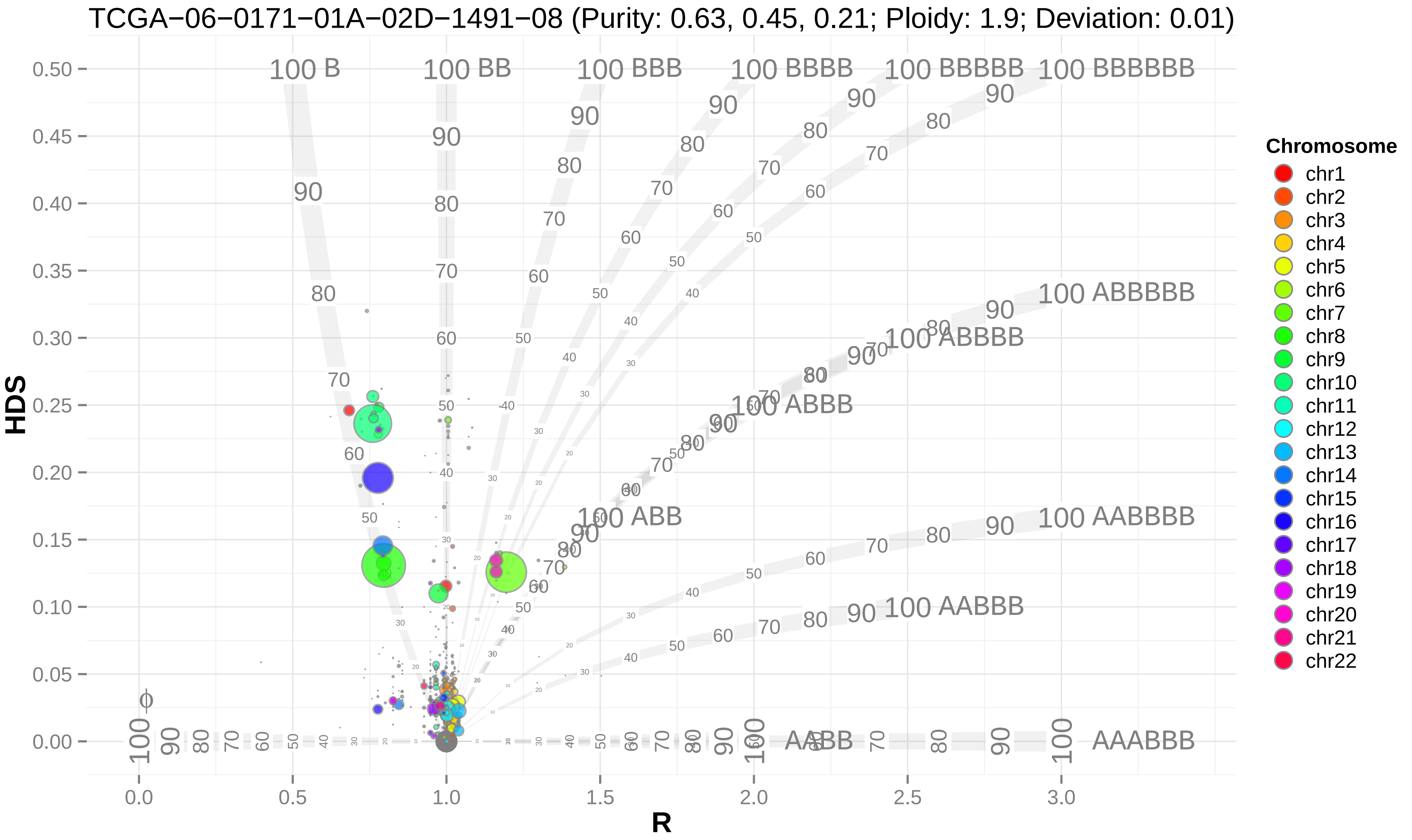
TCGA−06−0171−01A−02D−1491−08 (Purity: 0.63, 0.45, 0.21; Ploidy: 1.9; Deviation: 0.01)
HDS
R
Chromosome
chr1
chr2
chr3
chr4
chr5
chr6
chr7
chr8
chr9
chr10
chr11
chr12
chr13
chr14
chr15
chr16
chr17
chr18
chr19
chr20
chr21
chr22
100 B
100 BB
100 BBB
100 BBBB
100 BBBBB
100 BBBBBB
100 ABBBBB
100 ABBBB
100 ABBB
100 ABB
100 AABBBB
100 AABBB
AABB
AAABBB

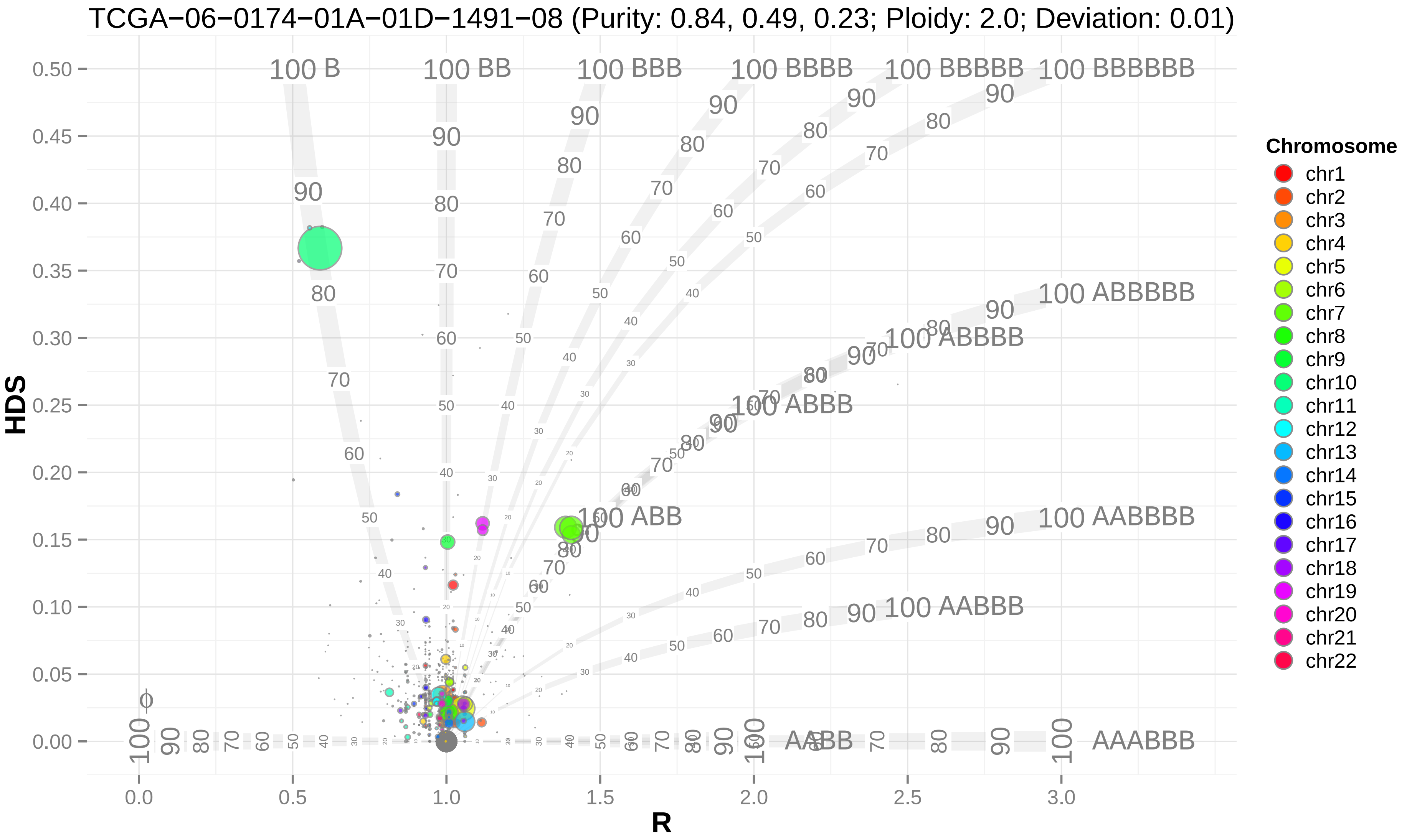
TCGA−06−0174−01A−01D−1491−08 (Purity: 0.84, 0.49, 0.23; Ploidy: 2.0; Deviation: 0.01)
HDS
R
100 B
100 BB
100 BBB
100 BBBB
100 BBBBB
100 BBBBBB
100 ABBBBB
100 ABBBB
100 ABBB
100 ABB
100 AABBBB
100 AABBB
AABB
AAABBB
Chromosome
chr1
chr2
chr3
chr4
chr5
chr6
chr7
chr8
chr9
chr10
chr11
chr12
chr13
chr14
chr15
chr16
chr17
chr18
chr19
chr20
chr21
chr22

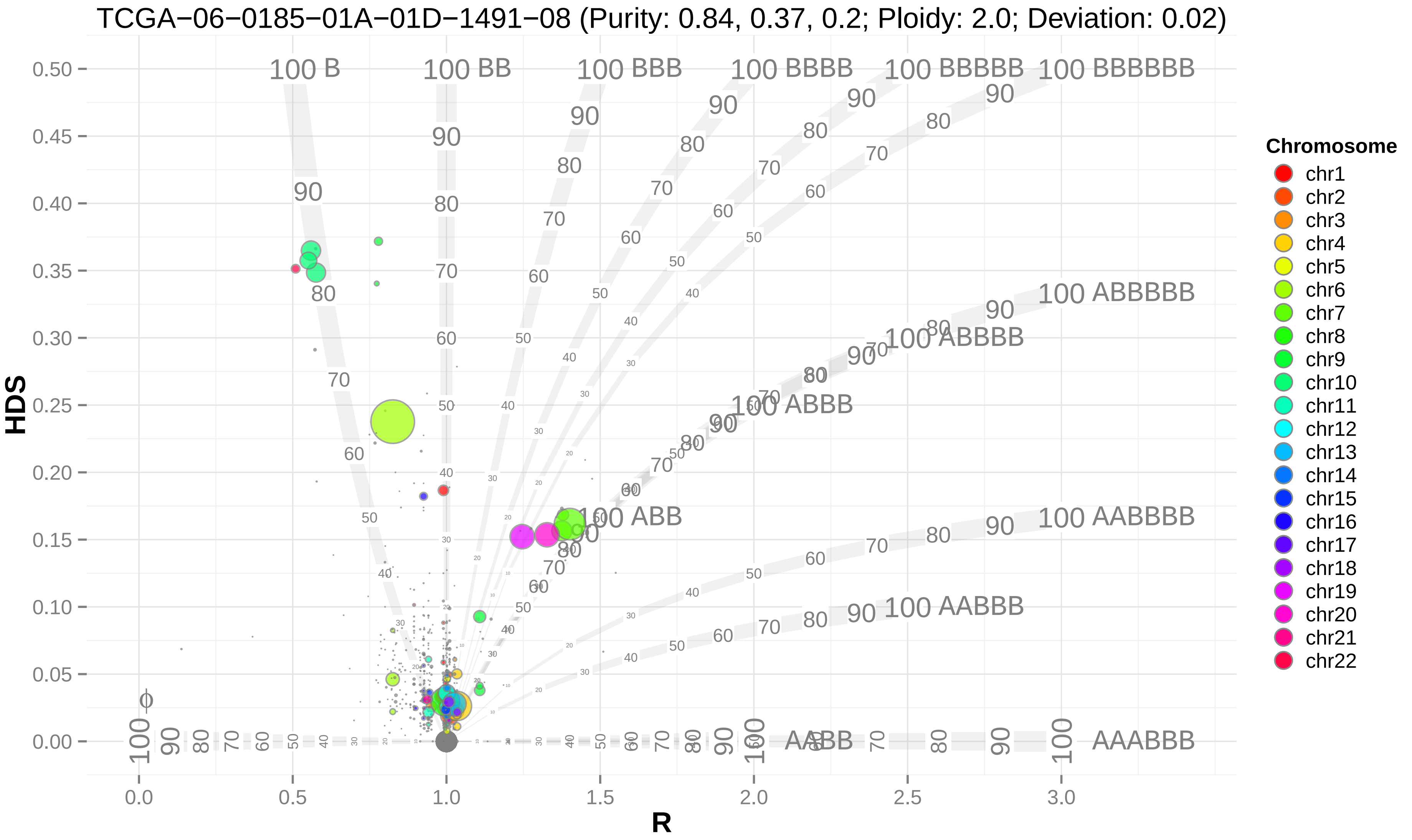
TCGA−06−0185−01A−01D−1491−08 (Purity: 0.84, 0.37, 0.2; Ploidy: 2.0; Deviation: 0.02)
HDS
R
100 B
100 BB
100 BBB
100 BBBB
100 BBBBB
100 BBBBBB
100 ABBBBB
100 ABBBB
100 ABBB
100 ABB
100 AABBBB
100 AABBB
AABB
AAABBB
Chromosome
chr1
chr2
chr3
chr4
chr5
chr6
chr7
chr8
chr9
chr10
chr11
chr12
chr13
chr14
chr15
chr16
chr17
chr18
chr19
chr20
chr21
chr22

TCGA−06−0188−01A−01D−1491−08 (Purity: 0.8, 0.61, 0.37, 0.23; Ploidy: 2.0; Deviation: 0.02)

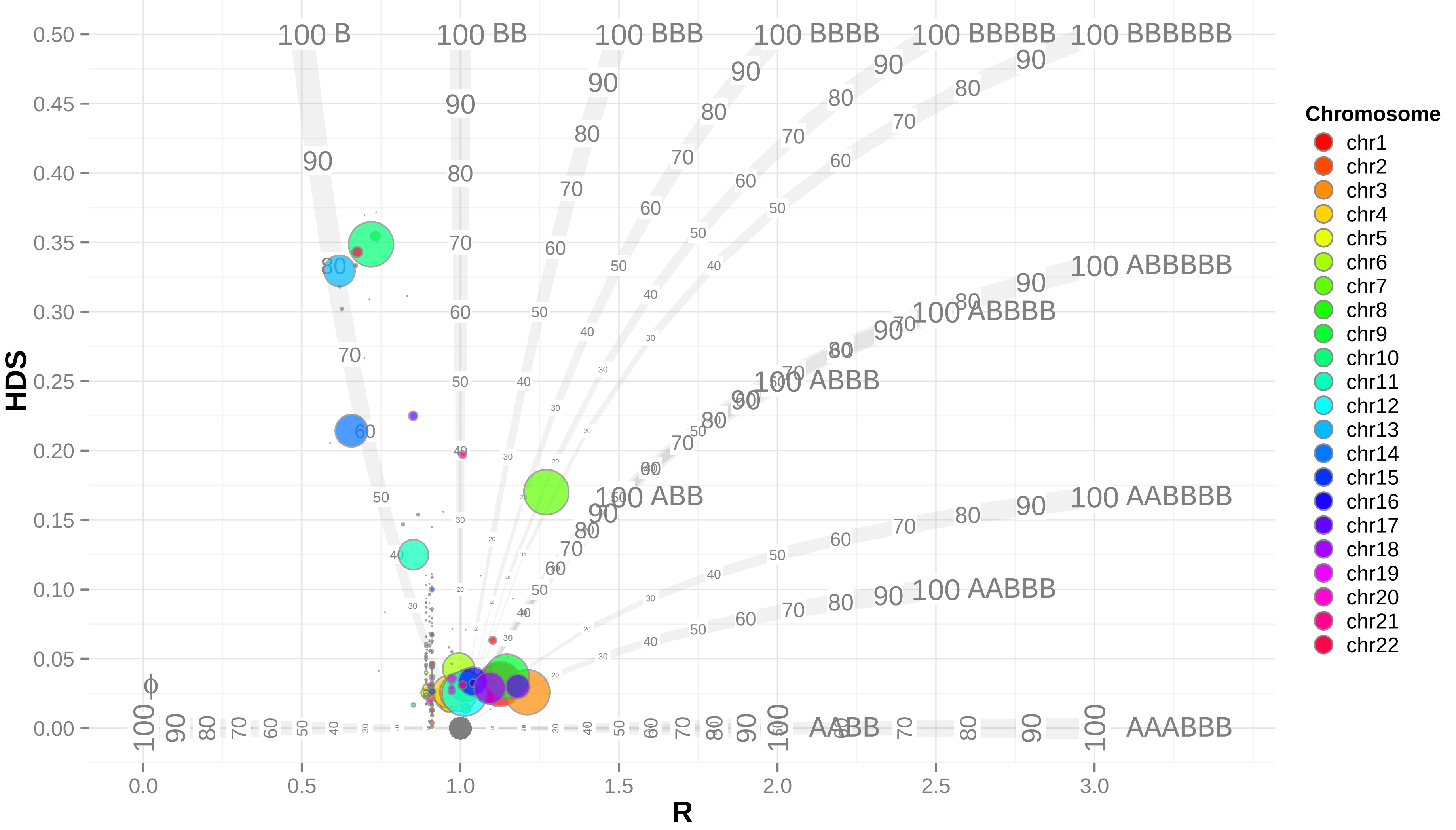

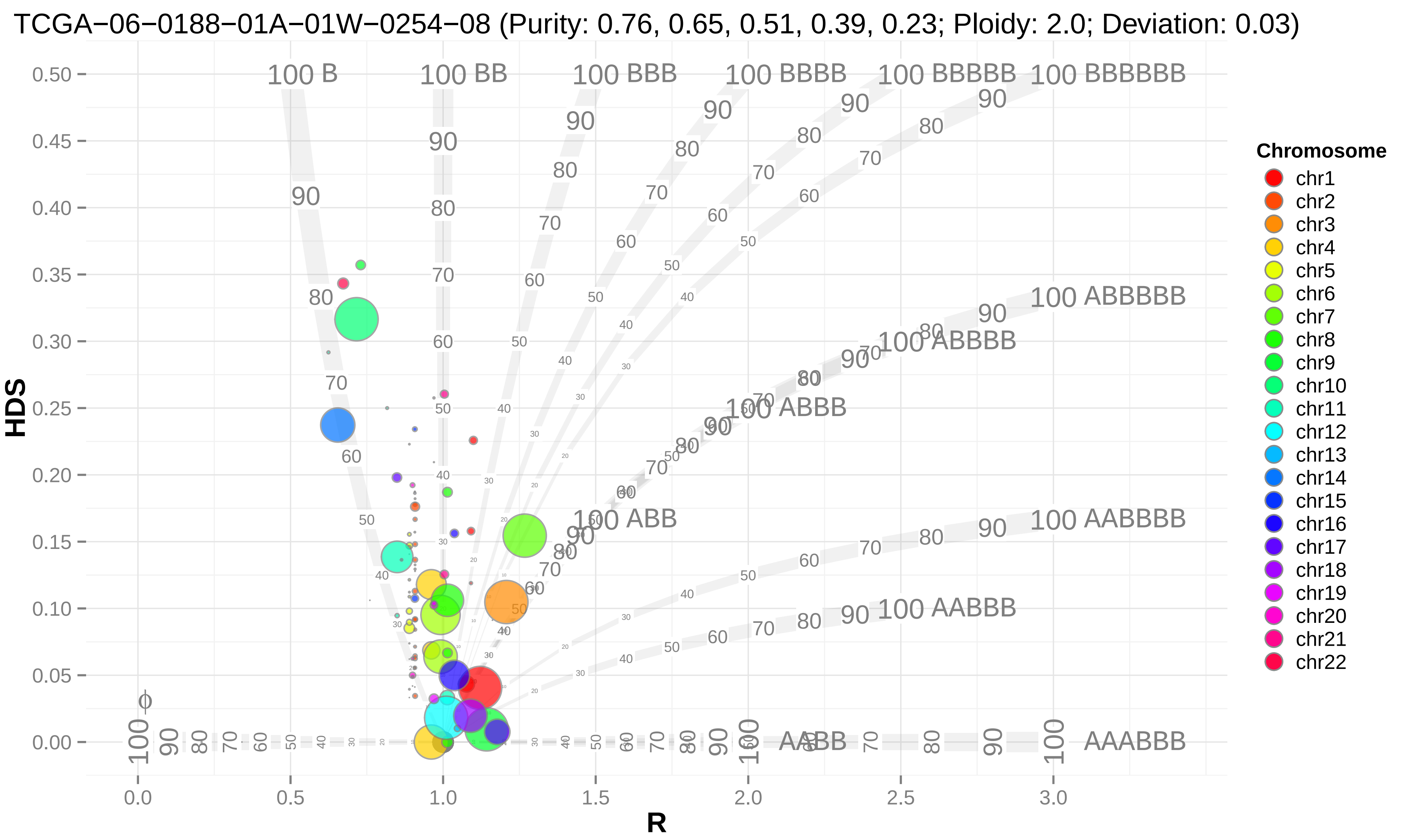
TCGA−06−0188−01A−01W−0254−08 (Purity: 0.76, 0.65, 0.51, 0.39, 0.23; Ploidy: 2.0; Deviation: 0.03)
HDS
R
Chromosome
chr1
chr2
chr3
chr4
chr5
chr6
chr7
chr8
chr9
chr10
chr11
chr12
chr13
chr14
chr15
chr16
chr17
chr18
chr19
chr20
chr21
chr22
100 B
100 BB
100 BBB
100 BBBB
100 BBBBB
100 BBBBBB
100 ABBBBB
100 ABBBB
100 ABBB
100 ABB
100 AABBBB
100 AABBB
AABB
AAABBB
ϕ

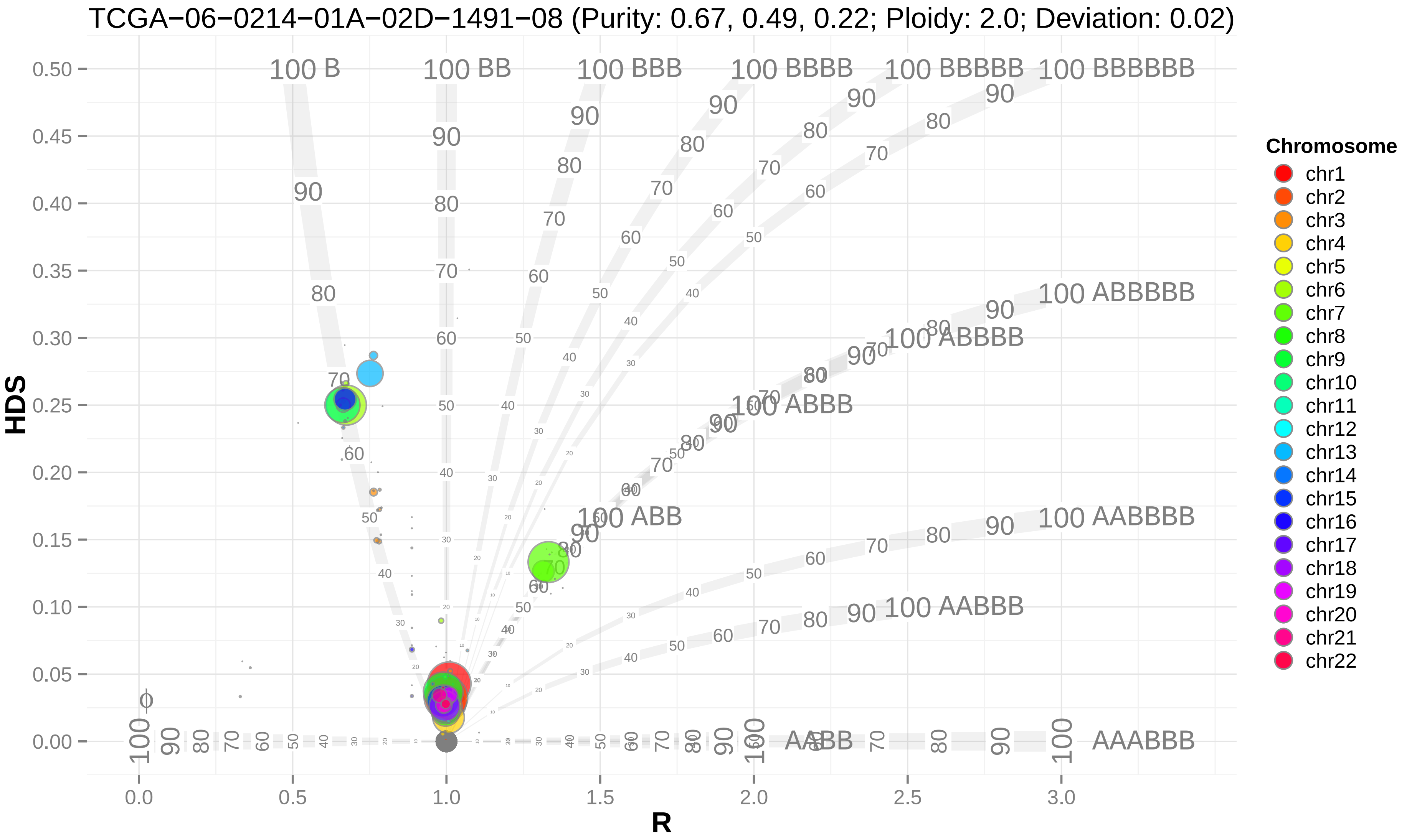
TCGA−06−0214−01A−02D−1491−08 (Purity: 0.67, 0.49, 0.22; Ploidy: 2.0; Deviation: 0.02)
HDS
R
Chromosome
chr1
chr2
chr3
chr4
chr5
chr6
chr7
chr8
chr9
chr10
chr11
chr12
chr13
chr14
chr15
chr16
chr17
chr18
chr19
chr20
chr21
chr22
100 B
100 BB
100 BBB
100 BBBB
100 BBBBB
100 BBBBBB
100 ABBBBB
100 ABBBB
100 ABBB
100 ABB
100 AABBBB
100 AABBB
AABB
AAABBB

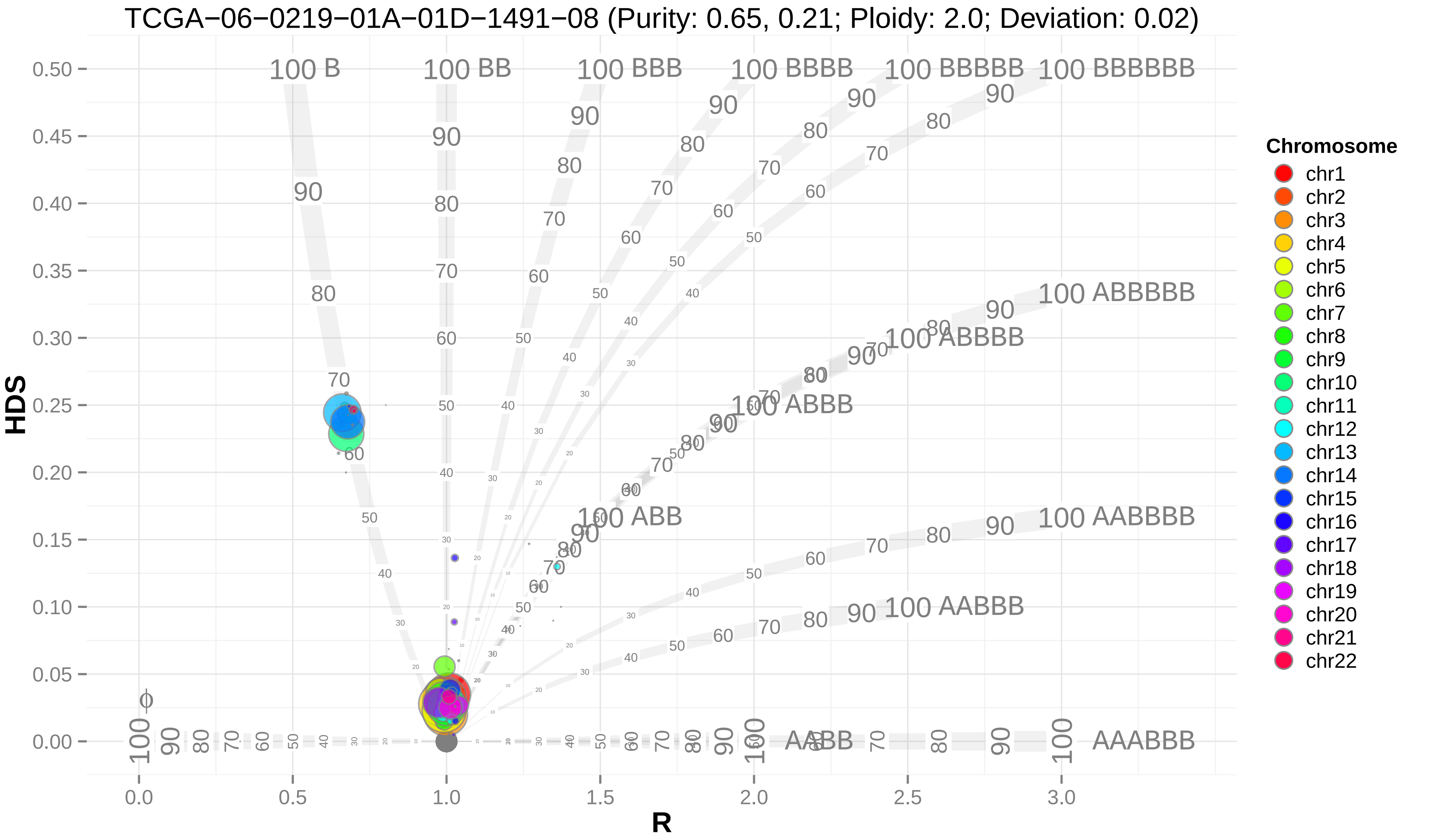
TCGA−06−0219−01A−01D−1491−08 (Purity: 0.65, 0.21; Ploidy: 2.0; Deviation: 0.02)
HDS
R
Chromosome
chr1
chr2
chr3
chr4
chr5
chr6
chr7
chr8
chr9
chr10
chr11
chr12
chr13
chr14
chr15
chr16
chr17
chr18
chr19
chr20
chr21
chr22
100 B
100 BB
100 BBB
100 BBBB
100 BBBBB
100 BBBBBB
100 ABBBBB
100 ABBBB
100 ABBB
100 ABB
100 AABBBB
100 AABBB
AABB
AAABBB

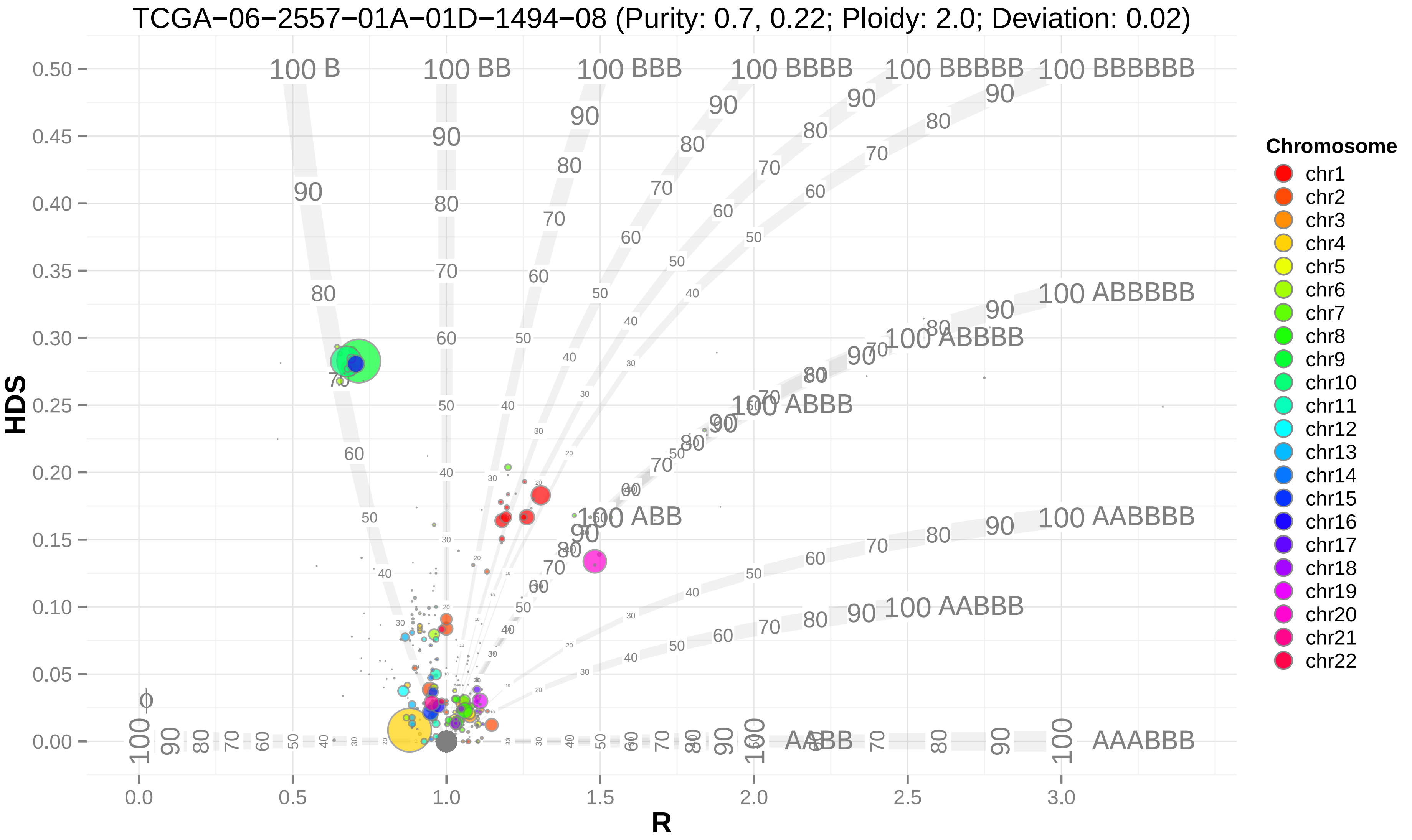
TCGA−06−2557−01A−01D−1494−08 (Purity: 0.7, 0.22; Ploidy: 2.0; Deviation: 0.02)
HDS
R
Chromosome
chr1
chr2
chr3
chr4
chr5
chr6
chr7
chr8
chr9
chr10
chr11
chr12
chr13
chr14
chr15
chr16
chr17
chr18
chr19
chr20
chr21
chr22
100 B
100 BB
100 BBB
100 BBBB
100 BBBBB
100 BBBBBB
100 ABBBBB
100 ABBBB
100 ABBB
100 ABB
100 AABBBB
100 AABBB
AABB
AAABBB

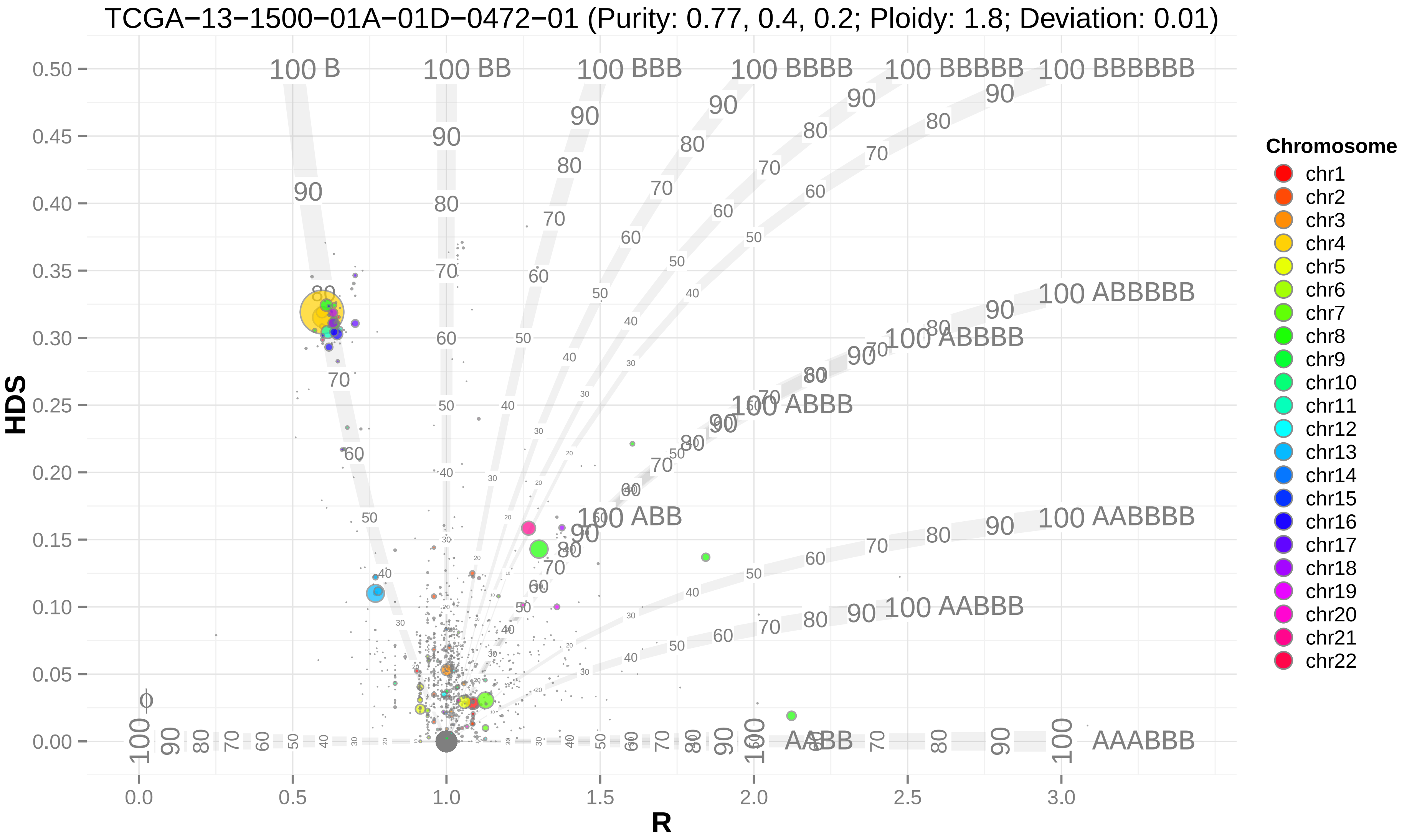
TCGA−13−1500−01A−01D−0472−01 (Purity: 0.77, 0.4, 0.2; Ploidy: 1.8; Deviation: 0.01)
HDS
R
Chromosome
chr1
chr2
chr3
chr4
chr5
chr6
chr7
chr8
chr9
chr10
chr11
chr12
chr13
chr14
chr15
chr16
chr17
chr18
chr19
chr20
chr21
chr22
100 B
100 BB
100 BBB
100 BBBB
100 BBBBB
100 BBBBBB
100 ABBBBB
100 ABBBB
100 ABBB
100 ABB
100 AABBBB
100 AABBB
AABB
AAABBB

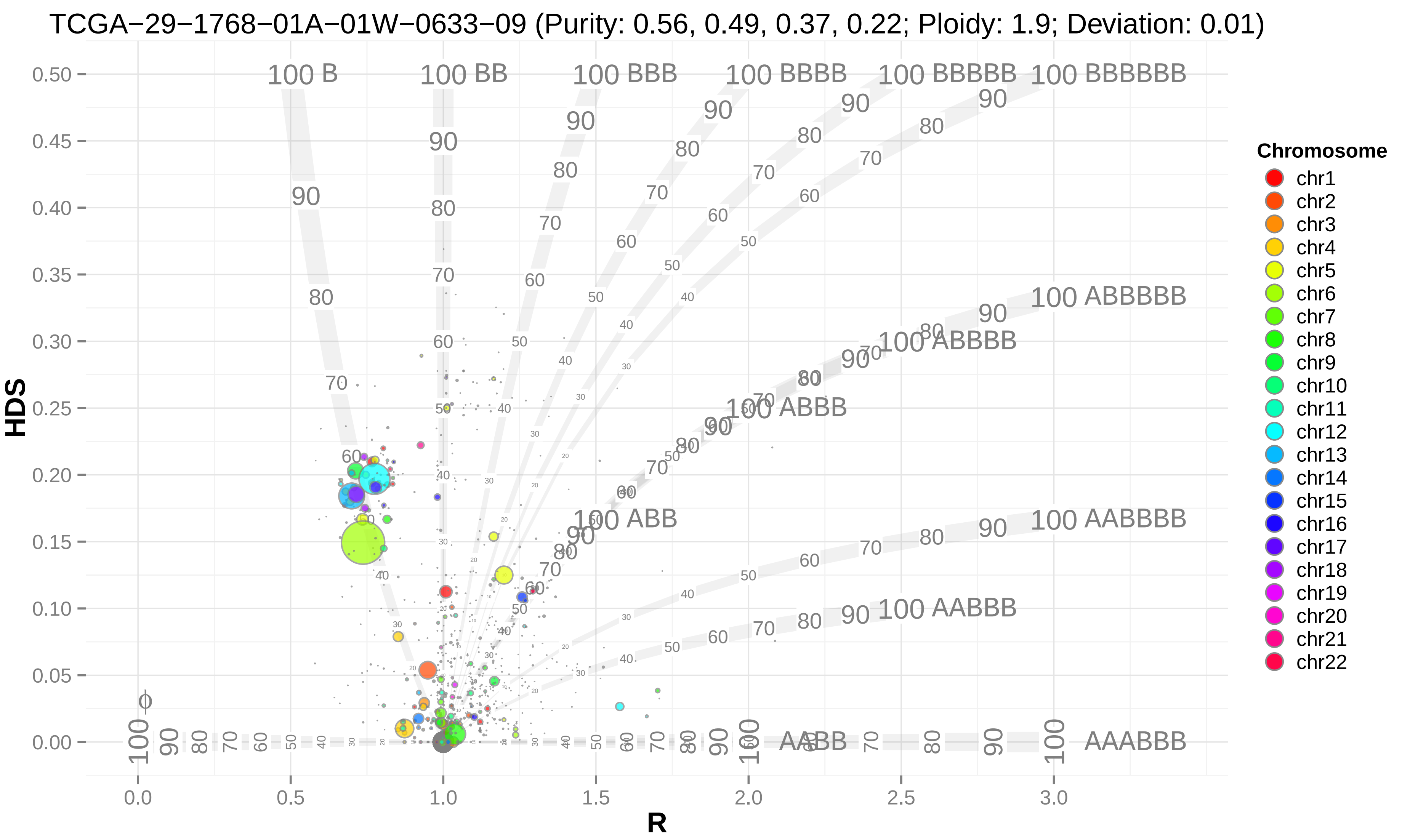
TCGA−29−1768−01A−01W−0633−09 (Purity: 0.56, 0.49, 0.37, 0.22; Ploidy: 1.9; Deviation: 0.01)
HDS
R
100 B
100 BB
100 BBB
100 BBBB
100 BBBBB
100 BBBBBB
100 ABBBBB
100 ABBBB
100 ABBB
100 ABB
100 AABBBB
100 AABBB
AABB
AAABBB
Chromosome
chr1
chr2
chr3
chr4
chr5
chr6
chr7
chr8
chr9
chr10
chr11
chr12
chr13
chr14
chr15
chr16
chr17
chr18
chr19
chr20
chr21
chr22

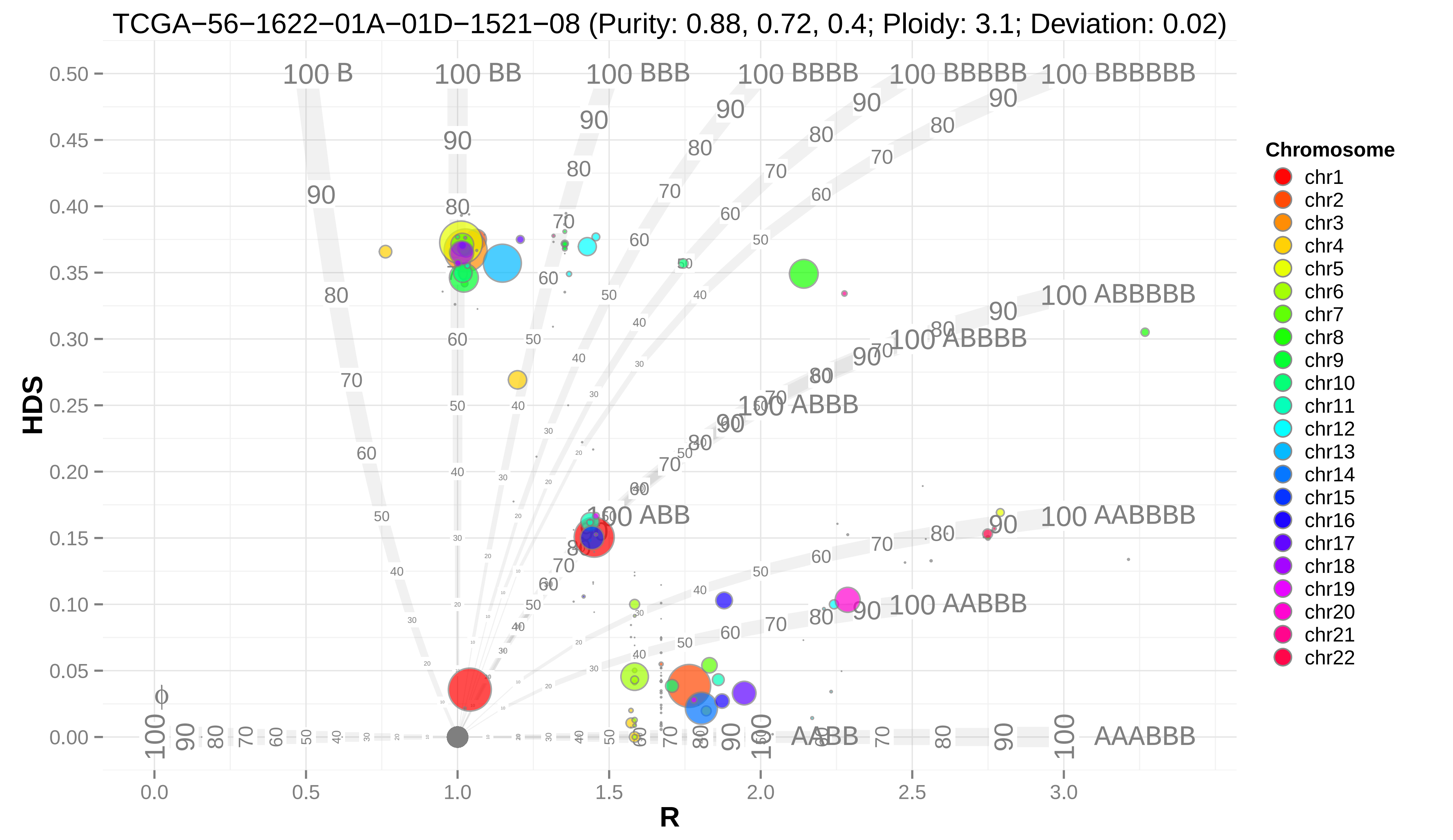
TCGA−56−1622−01A−01D−1521−08 (Purity: 0.88, 0.72, 0.4; Ploidy: 3.1; Deviation: 0.02)
HDS
R
Chromosome
chr1
chr2
chr3
chr4
chr5
chr6
chr7
chr8
chr9
chr10
chr11
chr12
chr13
chr14
chr15
chr16
chr17
chr18
chr19
chr20
chr21
chr22
100 B
100 BB
100 BBB
100 BBBB
100 BBBBB
100 BBBBBB
100 ABBBBB
100 ABBBB
100 ABBB
100 ABB
100 AABBBB
100 AABBB
AABB
AAABBB

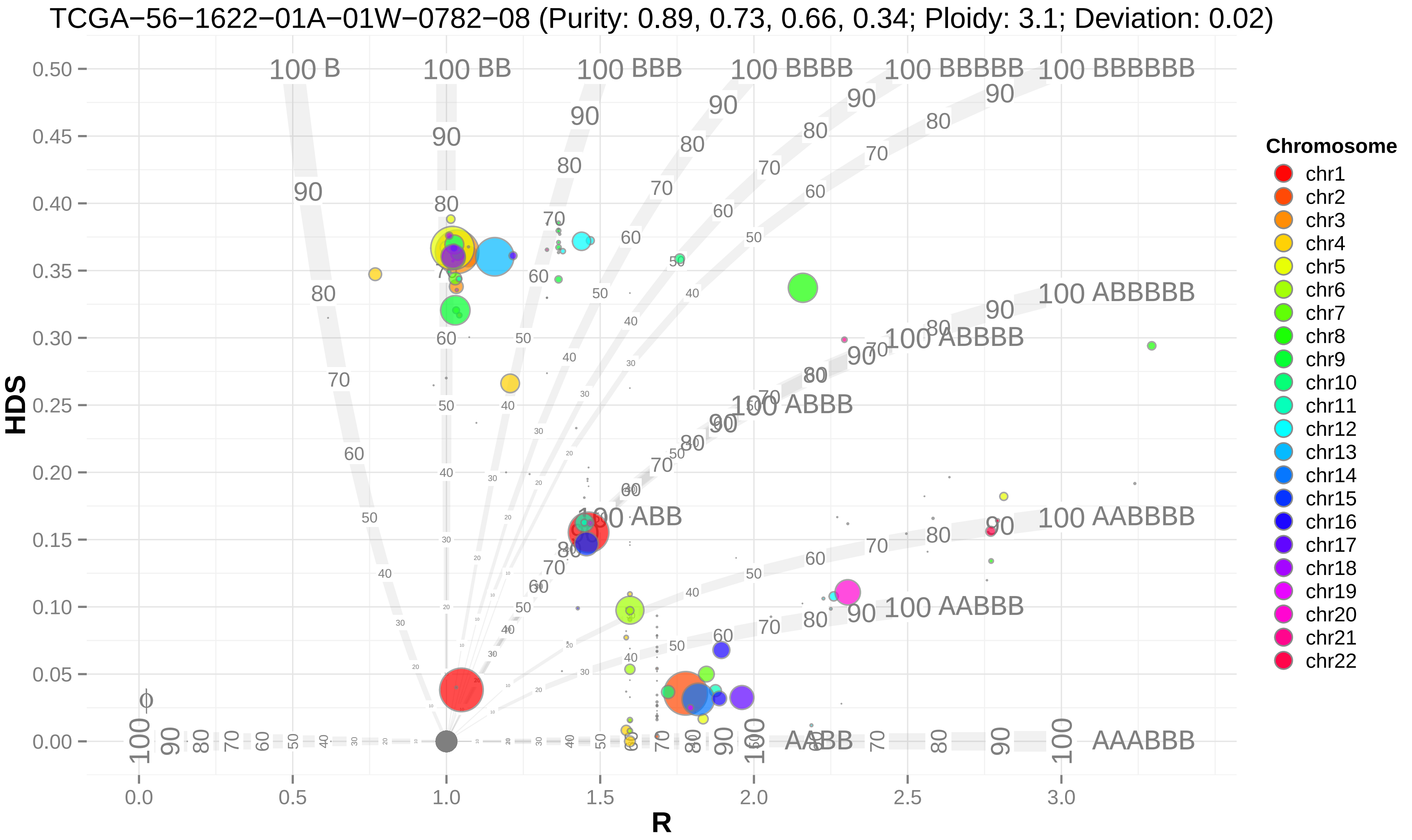
TCGA−56−1622−01A−01W−0782−08 (Purity: 0.89, 0.73, 0.66, 0.34; Ploidy: 3.1; Deviation: 0.02)
HDS
R
Chromosome
chr1
chr2
chr3
chr4
chr5
chr6
chr7
chr8
chr9
chr10
chr11
chr12
chr13
chr14
chr15
chr16
chr17
chr18
chr19
chr20
chr21
chr22
100 B
100 BB
100 BBB
100 BBBB
100 BBBBB
100 BBBBBB
100 ABBBBB
100 ABBBB
100 ABBB
100 ABB
100 AABBBB
100 AABBB
AABB
AAABBB

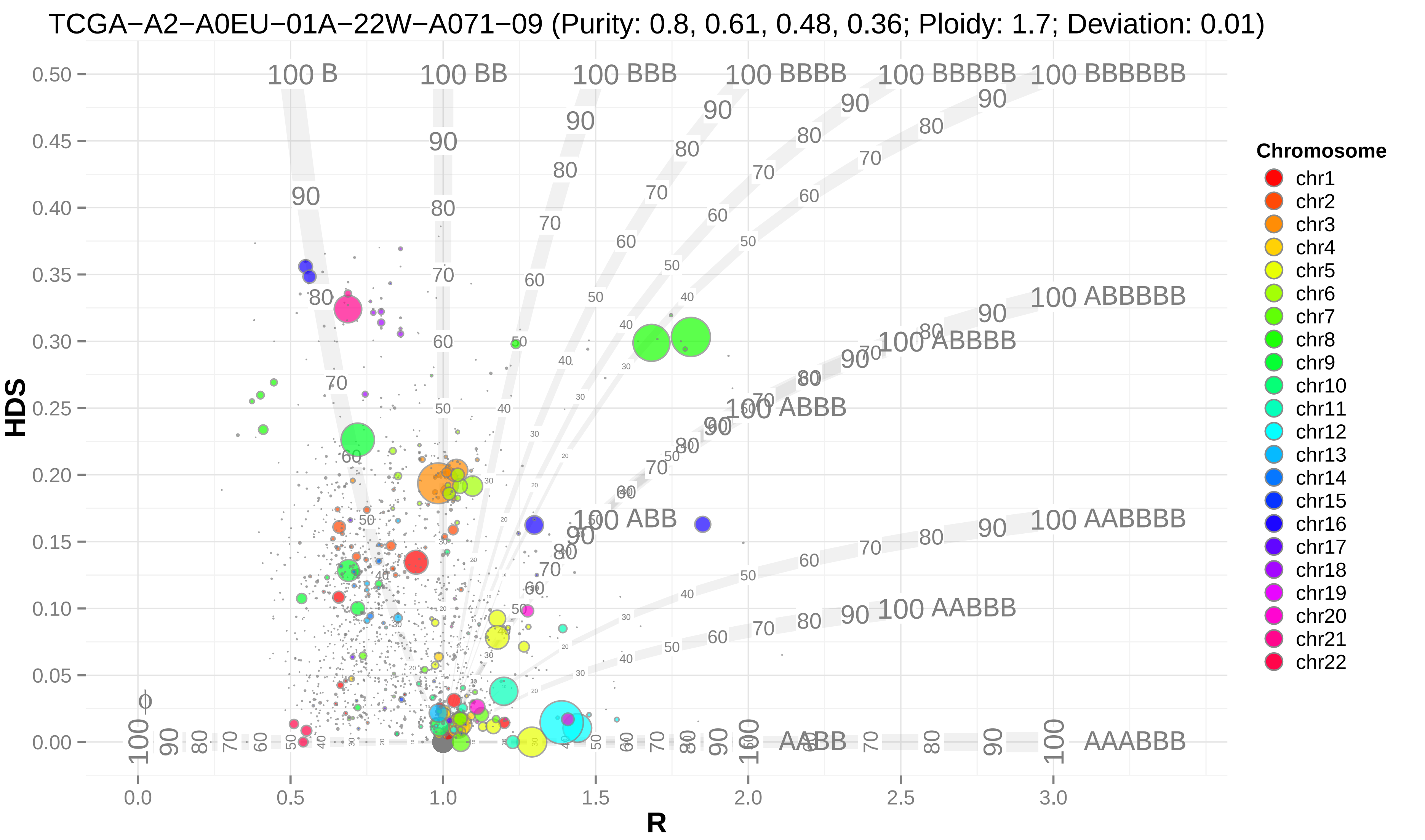

TCGA−A2−A0EU−01A−22W−A071−09 (Purity: 0.8, 0.61, 0.48, 0.36; Ploidy: 1.7; Deviation: 0.01)
HDS
R
100 B
100 BB
100 BBB
100 BBBB
100 BBBBB
100 BBBBBB
100 ABBBBB
100 ABBBB
100 ABBB
100 ABB
100 AABBBB
100 AABBB
AABB
AAABBB
Chromosome
chr1
chr2
chr3
chr4
chr5
chr6
chr7
chr8
chr9
chr10
chr11
chr12
chr13
chr14
chr15
chr16
chr17
chr18
chr19
chr20
chr21
chr22

TCGA−AO−A0JF−01A−11W−A071−09 (Purity: 0.59, 0.39; Ploidy: 2.1; Deviation: 0.00)

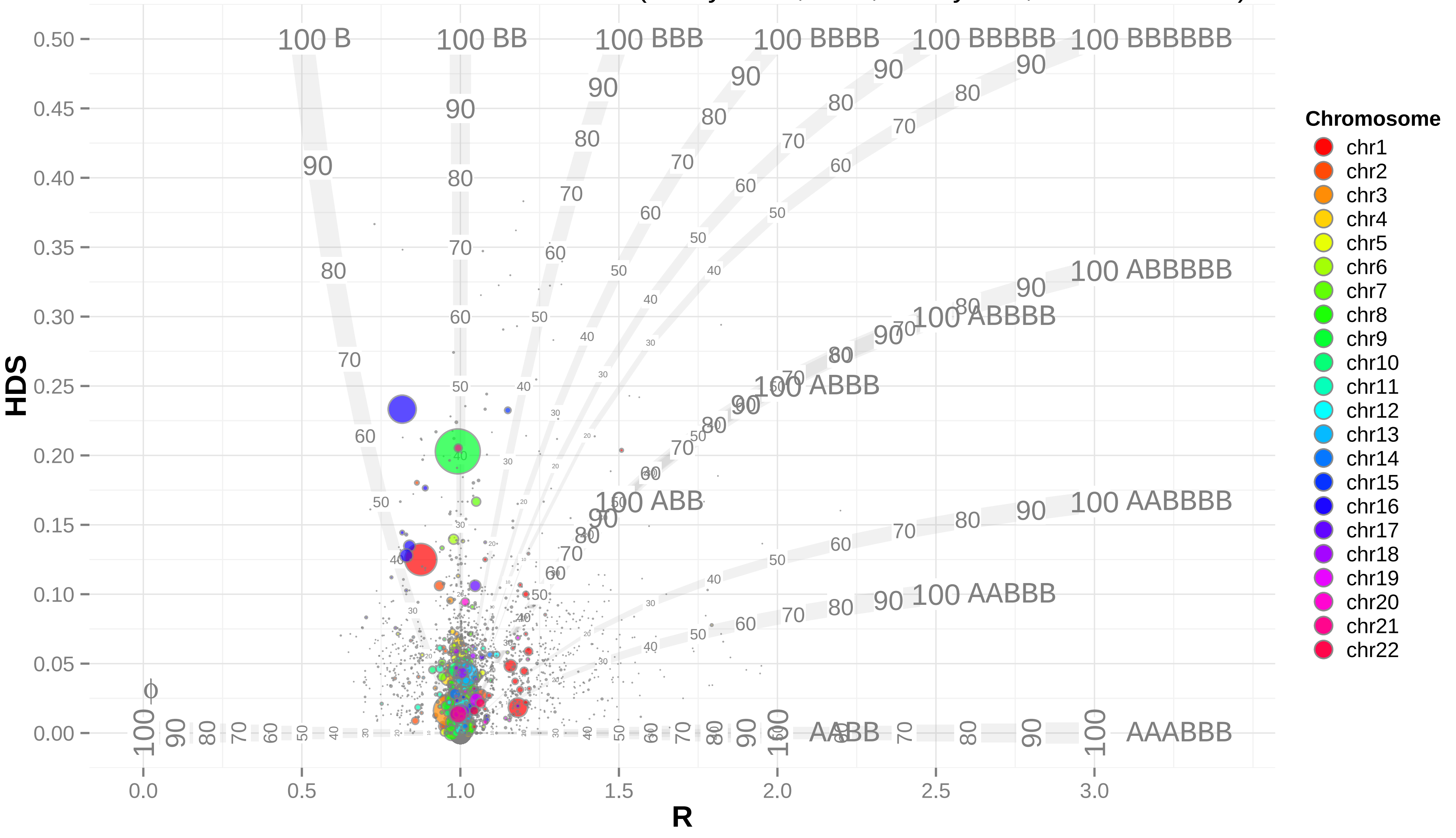

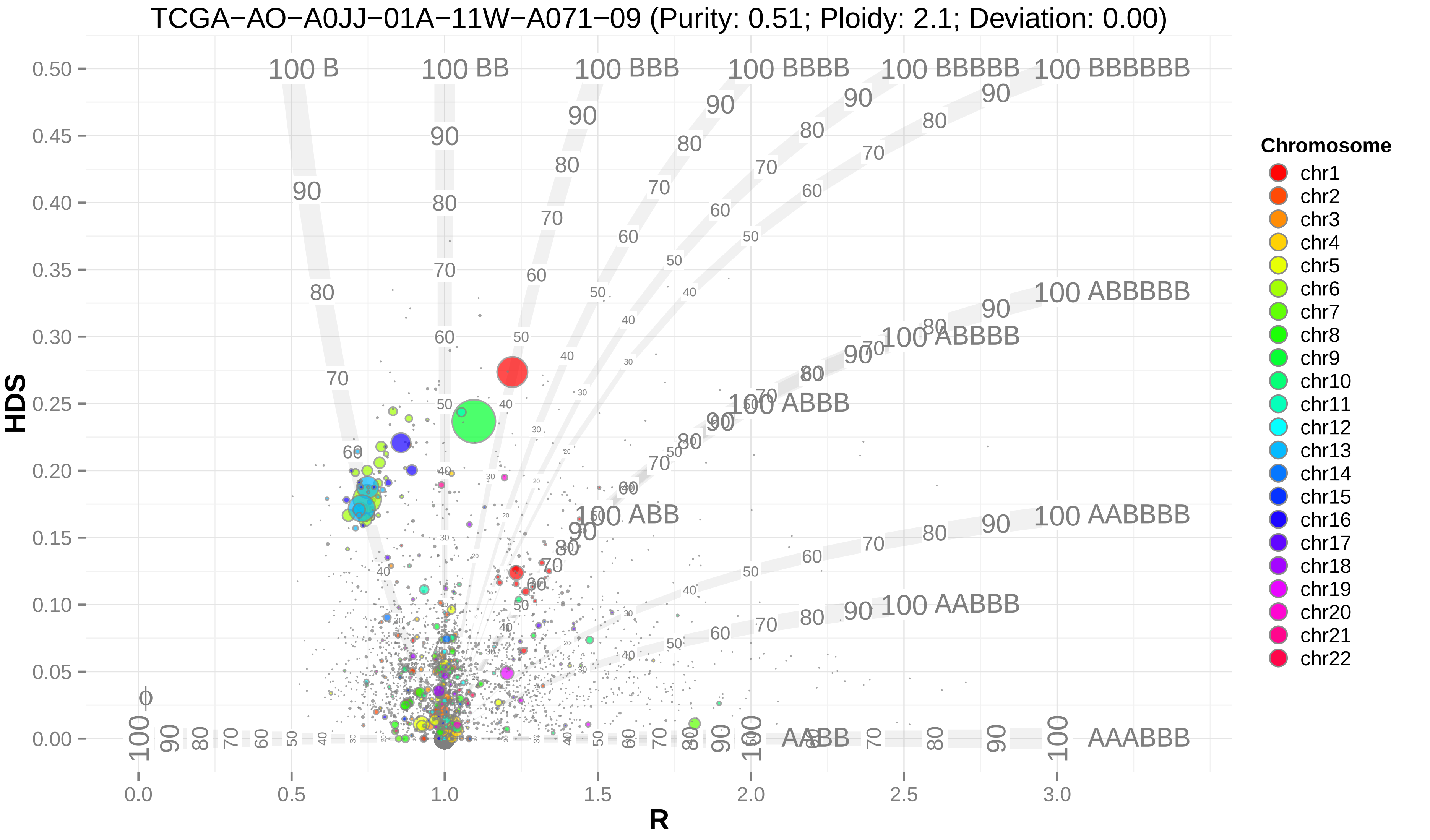
TCGA−AO−A0JJ−01A−11W−A071−09 (Purity: 0.51; Ploidy: 2.1; Deviation: 0.00)
HDS
R
0.00
0.05
0.10
0.15
0.20
0.25
0.30
0.35
0.40
0.45
0.50
0.0
0.5
1.0
1.5
2.0
2.5
3.0
100 B
100 BB
100 BBB
100 BBBB
100 BBBBB
100 BBBBBB
100 ABBBBB
100 ABBBB
100 ABBB
100 ABB
100 AABBBB
100 AABBB
AABB
AAABBB
ϕ
Chromosome
chr1
chr2
chr3
chr4
chr5
chr6
chr7
chr8
chr9
chr10
chr11
chr12
chr13
chr14
chr15
chr16
chr17
chr18
chr19
chr20
chr21
chr22

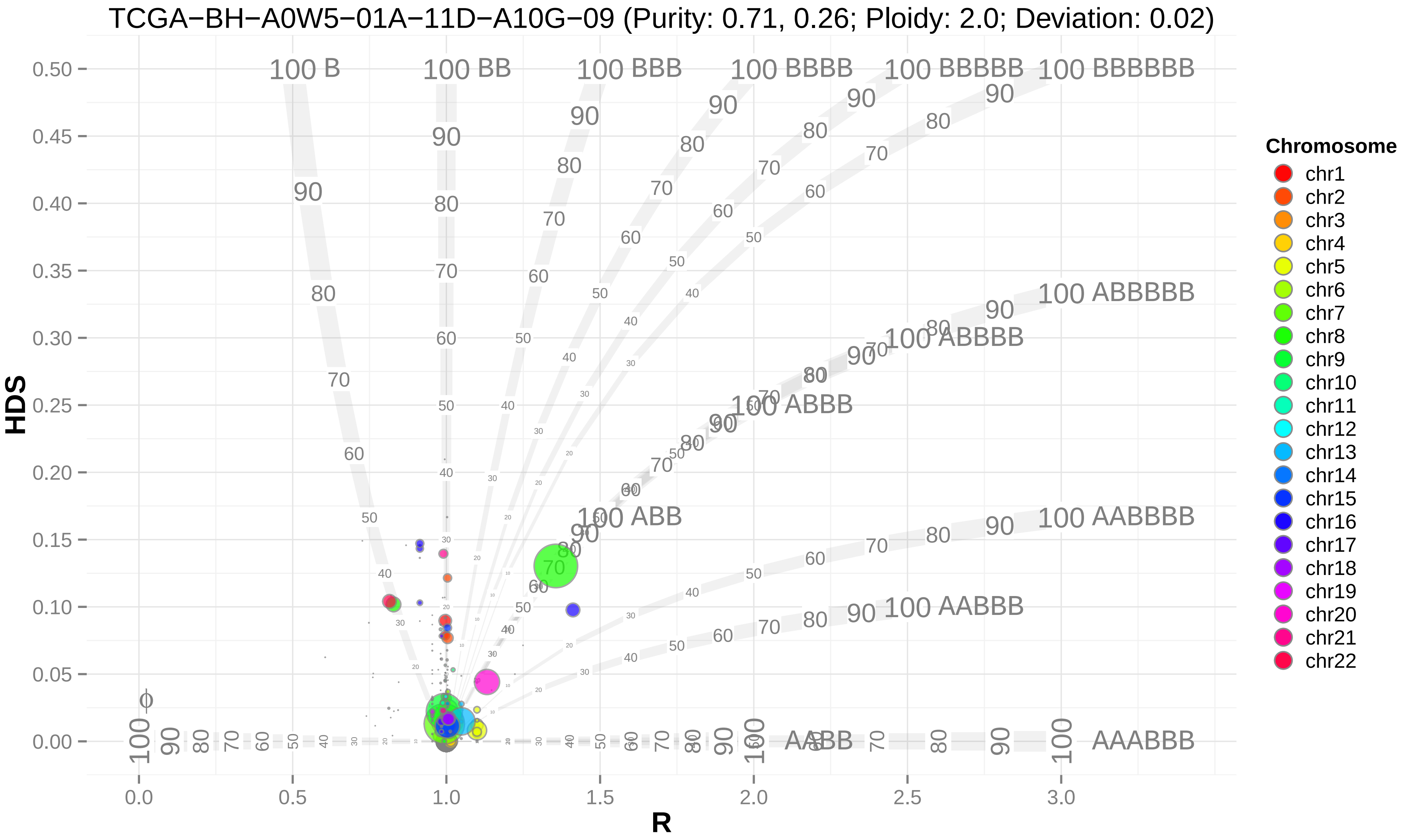

TCGA−BH−A0W5−01A−11D−A10G−09 (Purity: 0.71, 0.26; Ploidy: 2.0; Deviation: 0.02)
HDS
R
Chromosome
chr1
chr2
chr3
chr4
chr5
chr6
chr7
chr8
chr9
chr10
chr11
chr12
chr13
chr14
chr15
chr16
chr17
chr18
chr19
chr20
chr21
chr22
100 B
100 BB
100 BBB
100 BBBB
100 BBBBB
100 BBBBBB
100 ABBBBB
100 ABBBB
100 ABBB
100 ABB
100 AABBBB
100 AABBB
AABB
AAABBB

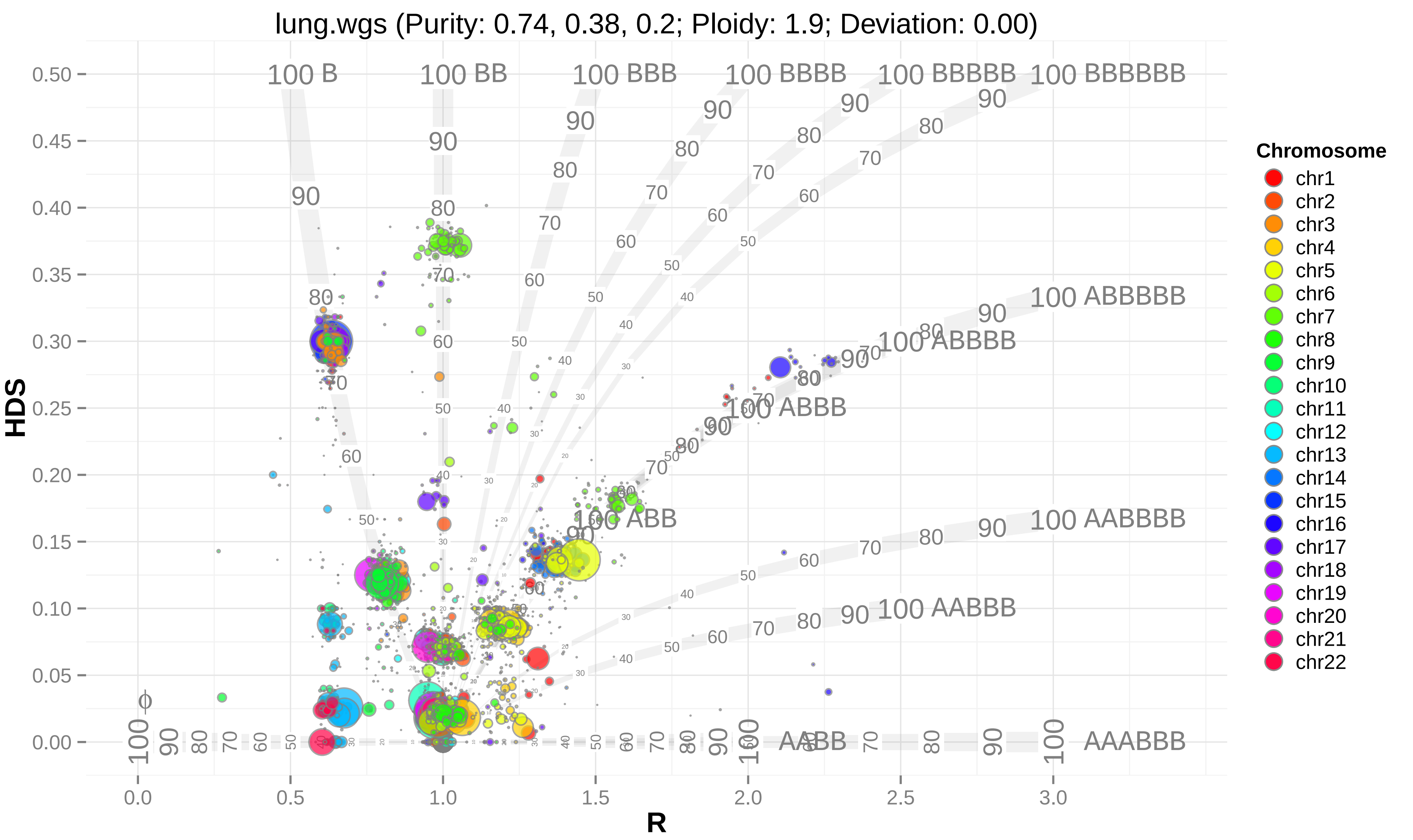

lung.wgs (Purity: 0.74, 0.38, 0.2; Ploidy: 1.9; Deviation: 0.00)
HDS
R
Chromosome
chr1
chr2
chr3
chr4
chr5
chr6
chr7
chr8
chr9
chr10
chr11
chr12
chr13
chr14
chr15
chr16
chr17
chr18
chr19
chr20
chr21
chr22
100 B
100 BB
100 BBB
100 BBBB
100 BBBBB
100 BBBBBB
100 ABBBBB
100 ABBBB
100 ABBB
100 ABB
100 AABBBB
100 AABBB
AABB
AAABBB

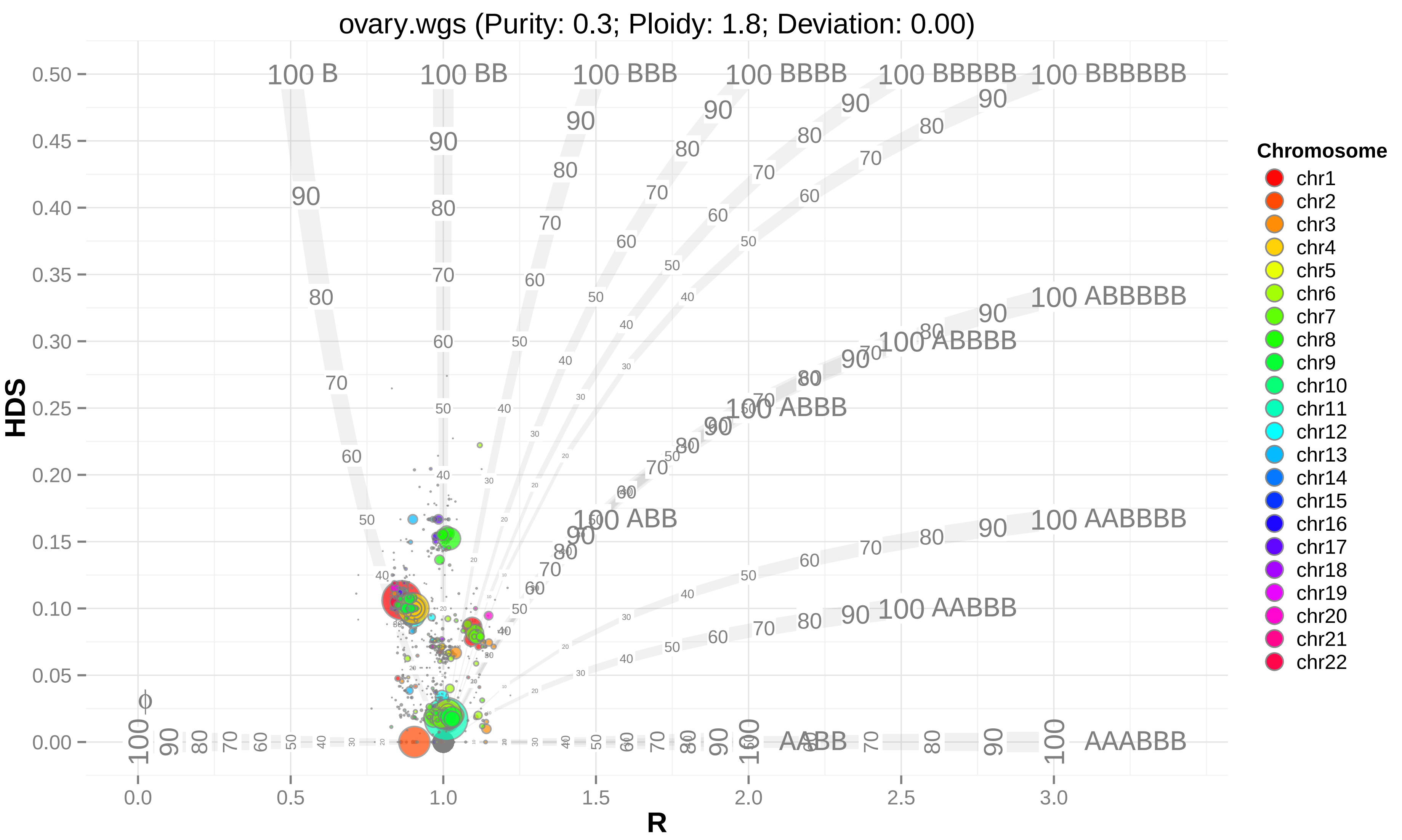
ovary.wgs (Purity: 0.3; Ploidy: 1.8; Deviation: 0.00)
HDS
R
100 B
100 BB
100 BBB
100 BBBB
100 BBBBB
100 BBBBBB
100 ABBBBB
100 ABBBB
100 ABBB
100 ABB
100 AABBBB
100 AABBB
AABB
AAABBB
Chromosome
chr1
chr2
chr3
chr4
chr5
chr6
chr7
chr8
chr9
chr10
chr11
chr12
chr13
chr14
chr15
chr16
chr17
chr18
chr19
chr20
chr21
chr22

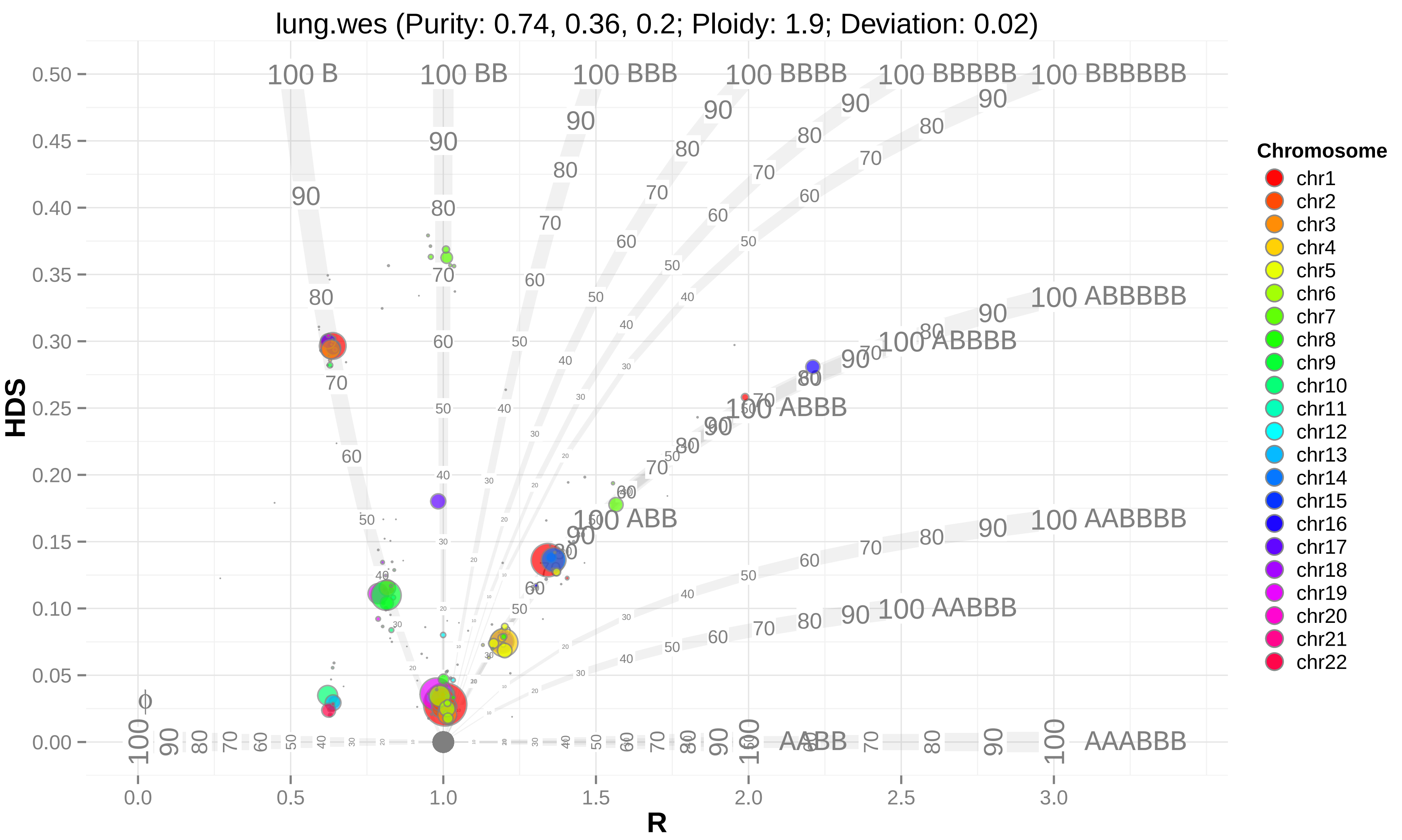
lung.wes (Purity: 0.74, 0.36, 0.2; Ploidy: 1.9; Deviation: 0.02)
HDS
R
Chromosome
chr1
chr2
chr3
chr4
chr5
chr6
chr7
chr8
chr9
chr10
chr11
chr12
chr13
chr14
chr15
chr16
chr17
chr18
chr19
chr20
chr21
chr22
100 B
100 BB
100 BBB
100 BBBB
100 BBBBB
100 BBBBBB
100 ABBBBB
100 ABBBB
100 ABBB
100 ABB
100 AABBBB
100 AABBB
AABB
AAABBB
ϕ

ovary.wes (Purity: 0.2; Ploidy: 2.0; Deviation: 0.03)

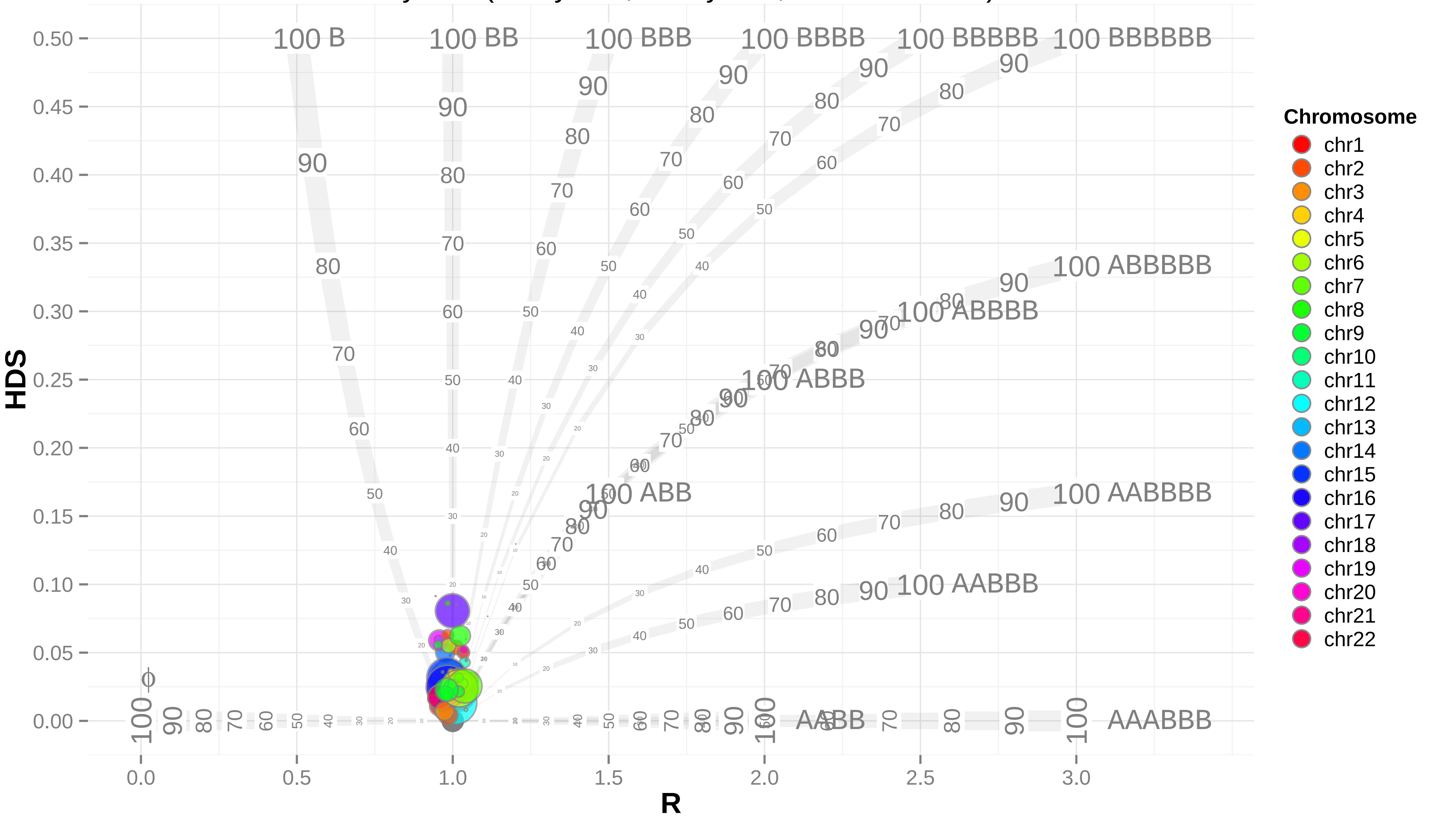

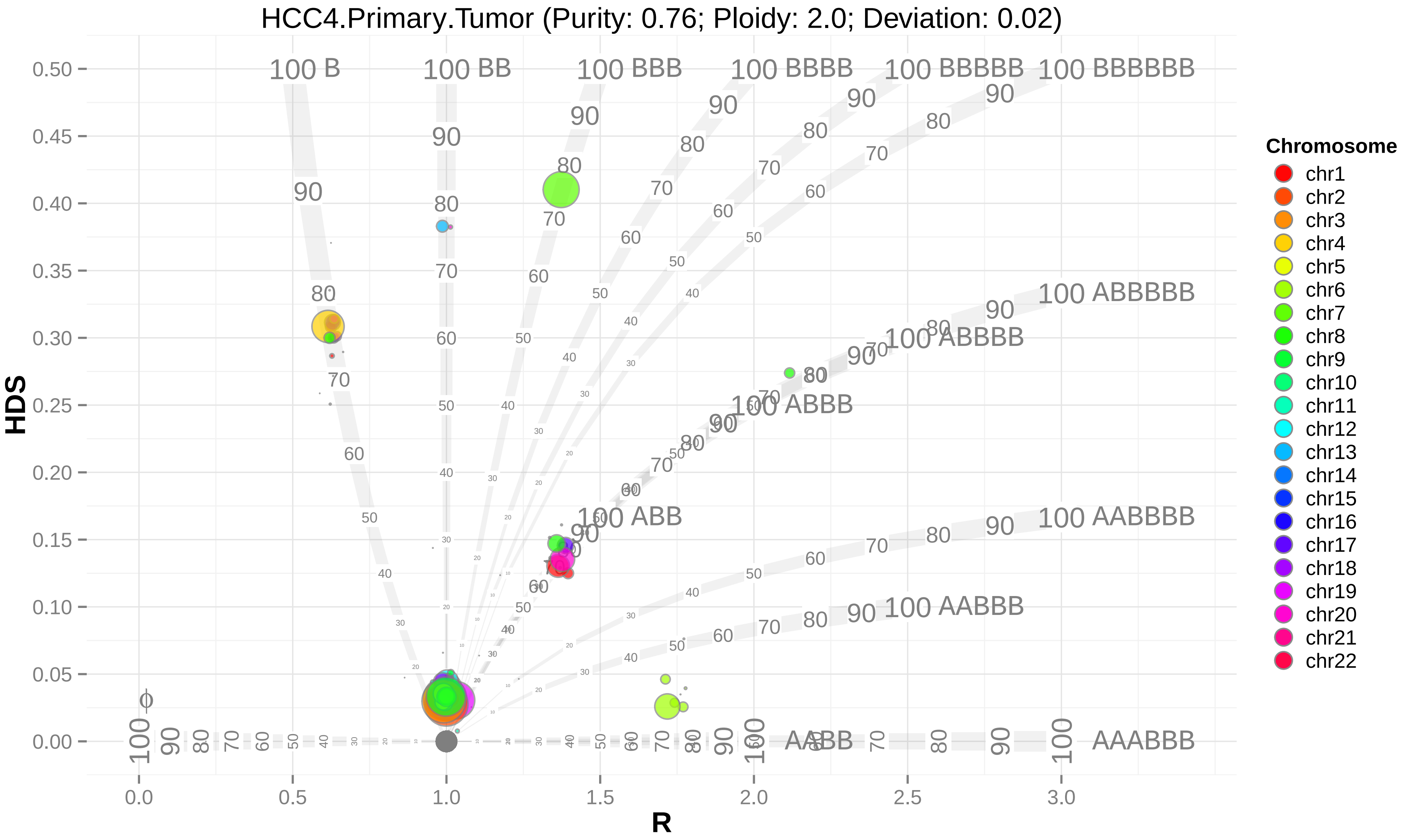
HCC4.Primary.Tumor (Purity: 0.76; Ploidy: 2.0; Deviation: 0.02)
HDS
R
Chromosome
chr1
chr2
chr3
chr4
chr5
chr6
chr7
chr8
chr9
chr10
chr11
chr12
chr13
chr14
chr15
chr16
chr17
chr18
chr19
chr20
chr21
chr22
100 B
100 BB
100 BBB
100 BBBB
100 BBBBB
100 BBBBBB
100 ABBBBB
100 ABBBB
100 ABBB
100 ABB
100 AABBBB
100 AABBB
AABB
AAABBB
ϕ

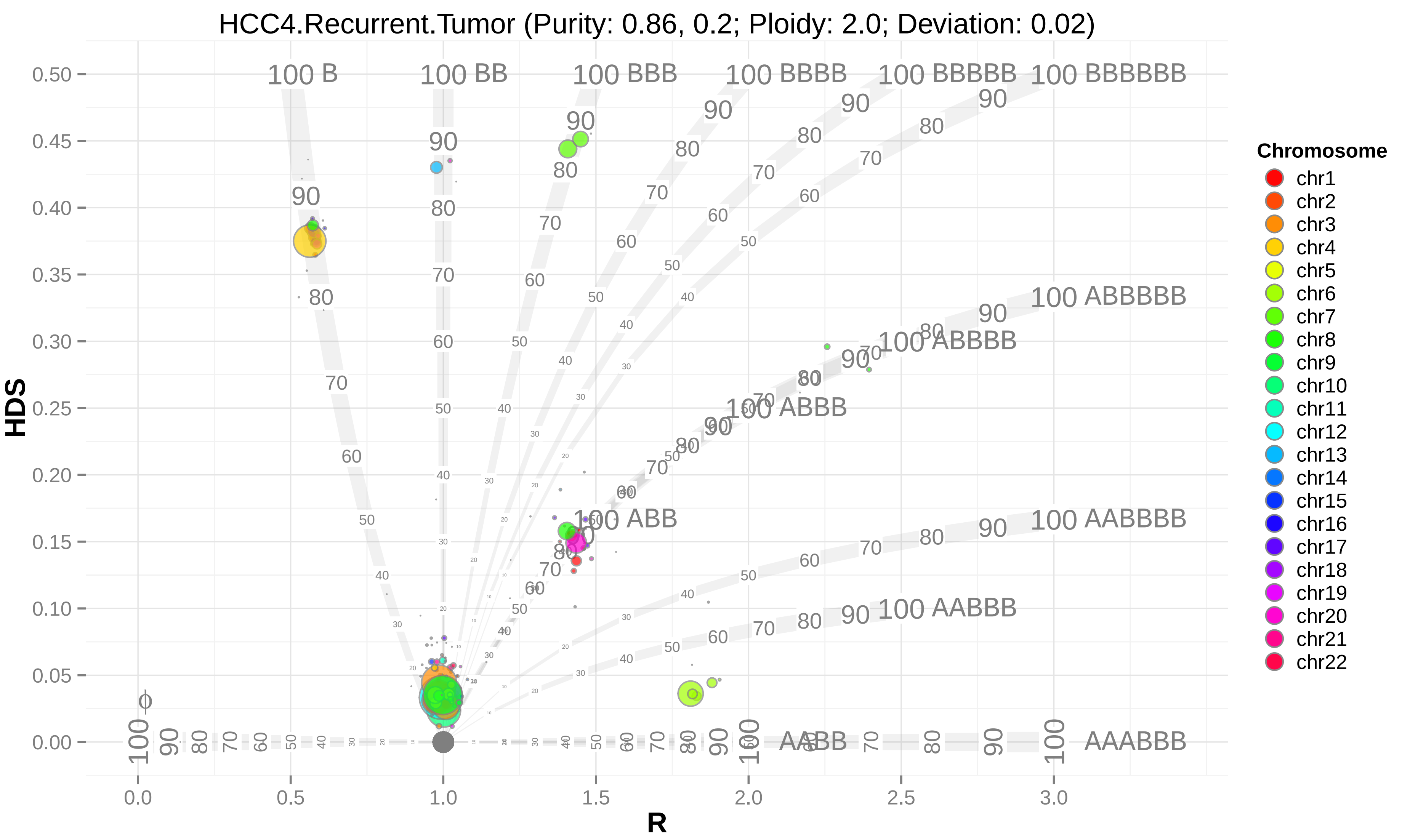
HCC4.Recurrent.Tumor (Purity: 0.86, 0.2; Ploidy: 2.0; Deviation: 0.02)
HDS
R
100 B
100 BB
100 BBB
100 BBBB
100 BBBBB
100 BBBBBB
100 ABBBBB
100 ABBBB
100 ABBB
100 ABB
100 AABBBB
100 AABBB
AABB
AAABBB
Chromosome
chr1
chr2
chr3
chr4
chr5
chr6
chr7
chr8
chr9
chr10
chr11
chr12
chr13
chr14
chr15
chr16
chr17
chr18
chr19
chr20
chr21
chr22

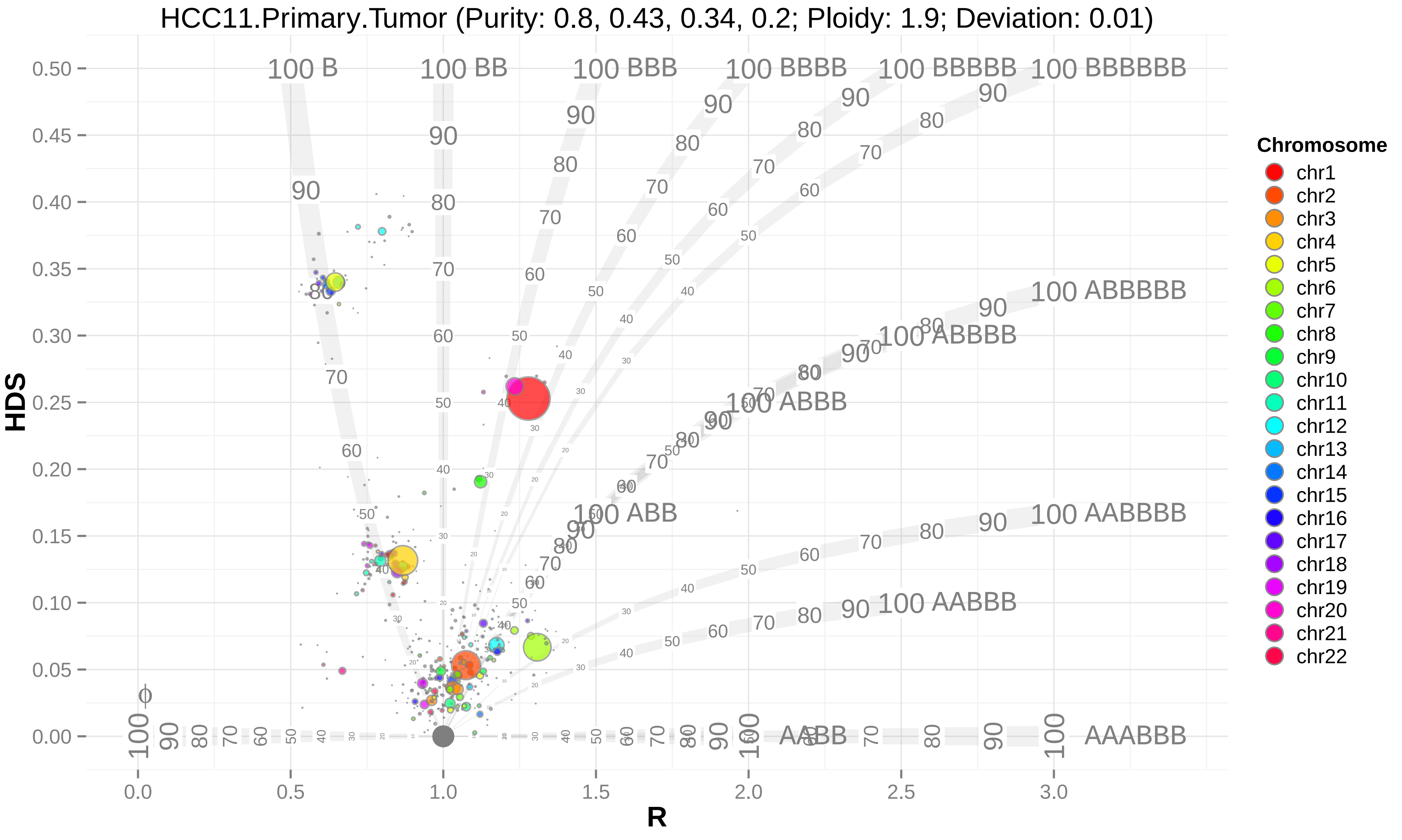
HCC11.Primary.Tumor (Purity: 0.8, 0.43, 0.34, 0.2; Ploidy: 1.9; Deviation: 0.01)
HDS
R
Chromosome
chr1
chr2
chr3
chr4
chr5
chr6
chr7
chr8
chr9
chr10
chr11
chr12
chr13
chr14
chr15
chr16
chr17
chr18
chr19
chr20
chr21
chr22
100 B
100 BB
100 BBB
100 BBBB
100 BBBBB
100 BBBBBB
100 ABBBBB
100 ABBBB
100 ABBB
100 ABB
100 AABBBB
100 AABBB
AABB
AAABBB

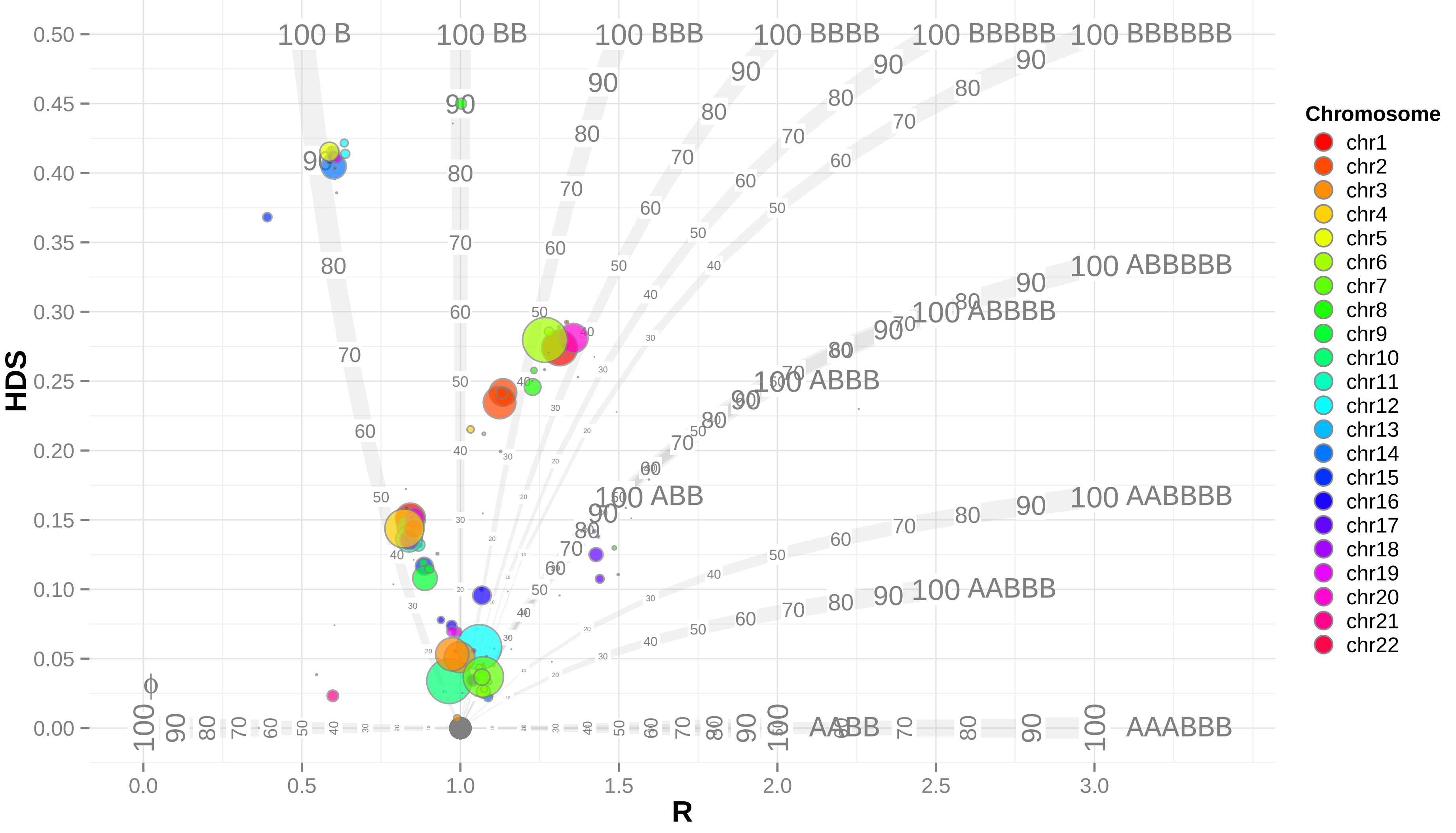

HCC11.Recurrent.Tumor (Purity: 0.9, 0.43, 0.35, 0.2; Ploidy: 2.0; Deviation: 0.02)
